# Exploring the link between dietary inflammatory index and sleep disorders: Insights from NHANES and Mendelian randomization approach

**DOI:** 10.1097/MD.0000000000043170

**Published:** 2025-07-04

**Authors:** Junjie Jiang, Shan Huang, Wei Yao, Yi Yuan, Tao Huang, Zhongfang Xia

**Affiliations:** a Department of Otolaryngology, Wuhan Children’s Hospital, Tongji Medical College, Huazhong University of Science & Technology, Wuhan, PR China.

**Keywords:** dietary inflammatory index, genome-wide association studies, Mendelian randomization, National Health and Nutrition Examination Survey, sleep disorders

## Abstract

Dietary health and sleep disorders are significant global public health concerns. This study aims to explore the potential relationship between the dietary inflammatory index (DII) and sleep disorders, providing insights into how dietary factors may influence sleep quality. We utilized epidemiological data from 24,870 participants in the National Health and Nutrition Examination Survey from 2007 to 2018, employing logistic regression and restricted cubic spline (RCS) analysis to assess the relationship between DII and sleep disorders. Additionally, Mendelian randomization (MR) analysis was conducted to investigate the causal relationships of individual DII components with sleep disorders. Our MR analysis revealed that genetically determined selenium has a protective effect against sleep apnea (odds ratio [OR] = 0.92, 95% confidence intervals [CI] 0.86–0.98, *P* = .007), while genetically determined vitamin C and monounsaturated fatty acids also demonstrated protective effects against sleep–wake disorders (OR = 0.57, 95% CI 0.33–0.97, *P* = .039; OR = 0.08, 95% CI 0.01–0.74, *P* = .026). These findings support the potential role of an anti-inflammatory diet in improving sleep health. Our results also showed that multivariable logistic regression revealed that for each unit increase in DII, the risk of sleep disorders increased by 12.9% (*P* < .001). After adjusting for age, sex, race, education, household income, hypertension, and diabetes, the risk still increased by 11.2%. Restricted cubic spline analysis indicated a significant nonlinear relationship between DII and sleep disorders. Our study suggests that a higher DII significantly increases the risk of sleep disorders. An anti-inflammatory diet may provide a protective effect, highlighting the importance of dietary adjustments in preventive strategies for sleep-related health issues.

## 1. Introduction

Sleep disorders are a category of diseases characterized by disruptions in normal sleep patterns, manifesting as difficulties in falling asleep, maintaining sleep, or excessive daytime sleepiness. These disorders not only severely impair daily functioning and quality of life but are also frequently accompanied by comorbid conditions such as anxiety, depression, and cognitive impairment. Moreover, sleep disorders impose a significant economic burden on society. According to a report by the Chinese Sleep Research Society, more than one-quarter of the population is affected by sleep disorders, while this proportion approaches one-third globally. With technological advancements and drastic changes in lifestyle, such as the widespread use of electronic devices and night shifts, the prevalence of sleep disorders continues to rise.^[1]^ The American Academy of Sleep Medicine and the Sleep Research Society further pointed out that sleep disorders are closely associated with various health issues, including cardiovascular diseases, mental health problems, immune system disorders, cancer, pain, and increased mortality.^[2,3]^ However, despite the significant challenge that sleep health poses in the field of public health, it has not received adequate attention. Furthermore, even after decades of specialized research, our understanding of human sleep and its regulatory mechanisms remains limited.

Diet is an important controllable factor for improving sleep duration and quality,^[4]^ with inflammatory components in the diet playing a crucial role in regulating systemic inflammation and influencing sleep quality.^[5,6]^ The dietary inflammatory index (DII) is used to assess the overall inflammatory potential of the diet and is associated with systemic inflammatory markers. It was developed by Cavicchia et al,^[7]^ and later updated by Shivappa et al.^[8]^ Research indicates that inappropriate dietary patterns are closely associated with elevated inflammatory markers. These inflammation levels can affect health by influencing sleep quality. Pro-inflammatory foods, such as refined carbohydrates, saturated fats, sugary beverages, fried foods, alcohol, and ω-6 fatty acids, have been shown to significantly increase systemic inflammatory biomarkers, including C-reactive protein, interleukin (IL)-6, and IL-10.^[9,10]^ Conversely, anti-inflammatory foods, such as fish, yogurt, rice, fruits, leafy greens, and nuts, have been demonstrated to reduce these inflammatory markers.^[11,12]^ Therefore, following an anti-inflammatory diet to modulate systemic inflammation may be a promising approach to improving sleep health. However, although some observational studies have explored the association between dietary inflammation and sleep problems, robust evidence establishing a causal link through inflammatory mechanisms remains limited, and the findings are inconsistent across populations.

Previous studies examining the relationship between the DII and sleep outcomes have yielded inconsistent results. Some reported that higher DII scores are associated with poorer sleep quality and increased risk of sleep disturbances,^[13]^ while others found no significant association. These inconsistencies may be due to confounding variables or population-specific factors, highlighting the need for more rigorous causal investigation.^[14]^

The main objective of this study is to explore the relationship between the DII and sleep disorders, specifically to investigate whether higher DII scores are associated with an increased risk of sleep disorders. Our hypothesis is that higher DII scores are positively correlated with the risk of sleep disorders, and that certain nutrients may have protective effects against sleep disorders.

To achieve this goal, we employed 2 complementary methodologies: an observational analysis using data from the National Health and Nutrition Examination Survey (NHANES), and a causal inference approach using Mendelian randomization (MR). This combined approach enables the identification of observational associations and the assessment of potential causal relationships, thereby enhancing the robustness of the study.

## 2. Materials and methods

### 2.1. Study design

We performed a cross-sectional study using NHANES data to explore the relationship between DII and sleep disorders. The dataset comprised 6 consecutive NHANES cycles, drawn from a representative U.S. population to evaluate health status and disease burden, sourced from the National Center for Health Statistics. Additionally, a two-sample MR analysis was employed to investigate the causal relationships between specific DII components and different subtypes of sleep disorders, including sleep apnoea–hypopnea syndrome, sleep–wake disturbances, insomnia, and hypersomnia.

### 2.2. Cross‑sectional study by NHANES

**Figure 1.**
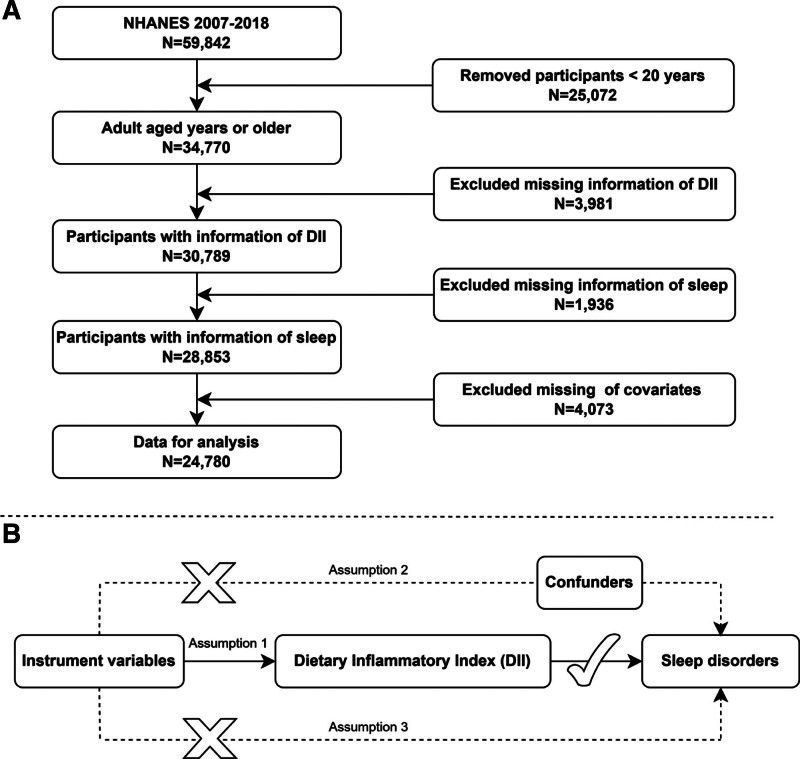
(A) Flow chart showing the selection process of eligible National Health and Nutrition Examination Survey (NHANES) participants included in this study. (B) The research is based on the following 3 hypotheses: (1) The instrumental variable is strongly correlated with the dietary inflammatory index (DII); (2) The instrumental variable is not related to confounding factors; (3) The instrumental variable is not directly associated with sleep disorders, and its effect on sleep disorders is only reflected through the DII.

This cross-sectional study used NHANES data. Excluding ineligible participants, 24,780 individuals were included in the final analysis. The detailed screening process is shown in Figure 1A.

Sleep disorders were the primary outcome of the study. Information on sleep disturbances was gathered from participants’ self-reported responses to the question: “In the past 2 weeks, how often have you experienced trouble sleeping or excessive sleep?” Participants who indicated “more than half the days” or “nearly every day” were classified as having a sleep disorder, while those who answered “several days” or “not at all” were not.^[15]^

The DII is calculated by adding up the scores assigned to each dietary component within a 24-hour period, which includes evaluations for both pro-inflammatory and anti-inflammatory foods. Initially, *Z*-scores are generated by deducting the global daily average intake from individual intakes and normalizing these values using the standard deviation.The scores are transformed into percentiles, doubled, and reduced by one to form a symmetrical arrangement. Each of these adjusted scores is multiplied by its specific “overall inflammatory effect score” to calculate the DII. Summing these values provides the aggregate “overall DII score.” In this analysis, the scores were derived using 28 specific nutrients, including macronutrients such as carbohydrates, proteins, and total fats, along with alcohol, fiber, cholesterol, saturated fatty acids (SFAs), monounsaturated fatty acids (MUFAs), and polyunsaturated fatty acids, omega-3 and omega-6 fatty acids, niacin, and a range of vitamins (A, B1, B2, B6, B12, C, D, and E), as well as minerals like iron, magnesium, zinc, selenium, and other compounds like folic acid, β-carotene, caffeine, and energy. The validity of the DII scoring method is maintained even if fewer than 30 nutrients are used.^[8]^

To mitigate bias, we collected various covariates, encompassing demographic details like age, gender, race, educational attainment, marital status, and annual household income. We also organized sleep-associated risk factors—namely, body mass index, smoking habits, alcohol use, hypertension, and diabetes—into a table. The criteria used to diagnose these factors are detailed in Table S1, Supplemental Digital Content, https://links.lww.com/MD/P364.

### 2.3. MR analysis

Figure 1B illustrates the study design and key assumptions of MR. We examined the summary genome-wide association studies (GWAS) for 28 nutrients related to DII and their links to sleep disorders, as outlined in Table S2, Supplemental Digital Content, https://links.lww.com/MD/P364. Genetic instruments included vitamins A, B1, B2, B6, B12, C, D, E, and nicotinamide, along with minerals like iron, magnesium, zinc, and selenium. Additional components were folic acid, β-carotene, caffeine, and energy, as well as key macronutrients: carbohydrates, protein, total fat, alcohol, fiber, and cholesterol. Furthermore, different types of fatty acids were included, such as SFAs, MUFAs, polyunsaturated fatty acids, and omega-3 and omega-6 fatty acids.

As β-carotene in circulation mainly exists in the form of carotene, we used GWAS data on circulating carotenoids as a proxy.^[16]^ Likewise, since riboflavin is a key part of vitamin B2, its GWAS data was used as a proxy for vitamin B2.^[17]^ After eliminating linkage disequilibrium (distance > 10,000 kb, *r*² < 0.001), the primary single nucleotide polymorphisms associated with each component of DII were identified in the GWAS data as instrumental variables (IVs, *P* < 5 × 10⁻⁶).

Sleep disorders, considered the outcome measure, encompass various conditions such as obstructive sleep apnea,^[18]^ sleep–wake disorders,^[19]^ insomnia,^[20]^ and hypersomnia.^[21]^ To further understand these conditions, subgroup analyses were conducted for different types of sleep disorders.

### 2.4. Statistical analysis

Given the NHANES complex multi-stage sampling framework, statistical analyses utilized sample weights to adjust for differences in selection probabilities. Each participant completed the first day’s dietary recall, and the corresponding “first-day dietary sample weight” was calculated based on the “least common denominator” formula (1/6 * wtdrd1). Continuous variables were shown as weighted means, and categorical variables were presented as counts (weighted %). The relationship between DII and sleep disorders was assessed using univariate logistic regression, with odds ratios (OR) and 95% confidence intervals (CI) to quantify the strength of the association. A restricted cubic spline (RCS) model was applied to further explore this relationship, and significance was determined with a *P*-value of <.05.

The main analytical method of this study employed the inverse-variance weighted (IVW) regression analysis with random effects. IVW aggregates estimates from genetic variants into a weighted average, with weights inversely proportional to their variance. This method assumes the validity of all genetic variants, thereby maximizing the statistical power of MR analysis, but remains vulnerable to pleiotropy bias.^[22]^ To verify the robustness of IVW estimates and reduce the risk of bias, we also conducted weighted median, MR-Egger, simple mode, and weighted mode analyses. These methods account for selection bias and acknowledge genetic heterogeneity.^[23]^ To further explore the causal effects of individual nutrients within DII on subgroups of sleep disorders, we conducted sensitivity analyses using Cochran IVW *Q* statistic to detect heterogeneity, MR-Egger regression intercept tests to assess horizontal pleiotropy,^[23]^ and MR-PRESSO for outlier identification.^[24]^ Results are expressed as ORs, representing the risk of sleep disorders per unit change in the DII, with 95% CIs. The *F*-statistic (*F* > 10) evaluated the IV strength. Additionally, a “leave-one-out” analysis identified heterogeneous IVs by sequentially excluding each genetic instrument. A *P*-value < .05 was deemed statistically significant. All analyses were performed using R software (version 4.4.0).

## 3. Result

### 3.1. Cross‑sectional study by NHANES

This section presents the observational analysis based on the NHANES dataset, which explores the association between the DII and sleep disorders. The study involved 24,780 NHANES participants, aged 20 or older, representing over 200 million U.S. community-dwelling residents. All participants provided complete data on DII and sleep conditions. Among them, 3994 (13.69%) had sleep disorders. Weighted baseline characteristics comparing participants with and without sleep disorders are shown in Table 1, highlighting significant differences in age, gender, race, education, marital status, household income to poverty ratio, smoking status, alcohol consumption, body mass index, diabetes, hypertension, and DII.

**Table 1:** Weighted baseline characteristics of participants.

	[ALL]	Sleep disorder	NC	*P*-overall
	N = 24,780	N = 3994	N = 20,786	
Age				<.001
20–40	8678 (35.0%)	1272 (31.8%)	7406 (35.6%)	
41–60	8347 (33.7%)	1470 (36.8%)	6877 (33.1%)	
61–80	7755 (31.3%)	1252 (31.3%)	6503 (31.3%)	
Gender				<.001
Male	12,260 (49.5%)	1672 (41.9%)	10,588 (50.9%)	
Female	12,520 (50.5%)	2322 (58.1%)	10,198 (49.1%)	
Education				<.001
Below high school	5564 (22.5%)	1123 (28.1%)	4441 (21.4%)	
High school or above	19,216 (77.5%)	2871 (71.9%)	16,345 (78.6%)	
Race				<.001
Mexican American	3641 (14.7%)	480 (12.0%)	3161 (15.2%)	
Non-Hispanic White	10,958 (44.2%)	1961 (49.1%)	8997 (43.3%)	
Non-Hispanic Black	5033 (20.3%)	795 (19.9%)	4238 (20.4%)	
Other	5148 (20.8%)	758 (19.0%)	4390 (21.1%)	
Marital				<.001
Yes	14,824 (59.8%)	2027 (50.8%)	12,797 (61.6%)	
No	9956 (40.2%)	1967 (49.2%)	7989 (38.4%)	
Obesity				<.001
<25	6920 (27.9%)	961 (24.1%)	5959 (28.7%)	
25–29	8122 (32.8%)	1146 (28.7%)	6976 (33.6%)	
≥30	9738 (39.3%)	1887 (47.2%)	7851 (37.8%)	
PIR				<.001
Poor	7729 (31.2%)	1682 (42.1%)	6047 (29.1%)	
Not poor	17,051 (68.8%)	2312 (57.9%)	14,739 (70.9%)	
Drink				.013
Yes	18,723 (75.6%)	3080 (77.1%)	15,643 (75.3%)	
No	6057 (24.4%)	914 (22.9%)	5143 (24.7%)	
Smoke				<.001
Yes	11,189 (45.2%)	2253 (56.4%)	8936 (43.0%)	
No	13,591 (54.8%)	1741 (43.6%)	11,850 (57.0%)	
DM				<.001
Yes	4500 (18.2%)	917 (23.0%)	3583 (17.2%)	
No	20,280 (81.8%)	3077 (77.0%)	17,203 (82.8%)	
Hyptension				<.001
Yes	10,687 (43.1%)	1992 (49.9%)	8695 (41.8%)	
No	14,093 (56.9%)	2002 (50.1%)	12,091 (58.2%)	
DII	1.53 (1.90)	1.83 (1.88)	1.47 (1.89)	<.001

DII = dietary inflammatory index.

**Figure 2.**
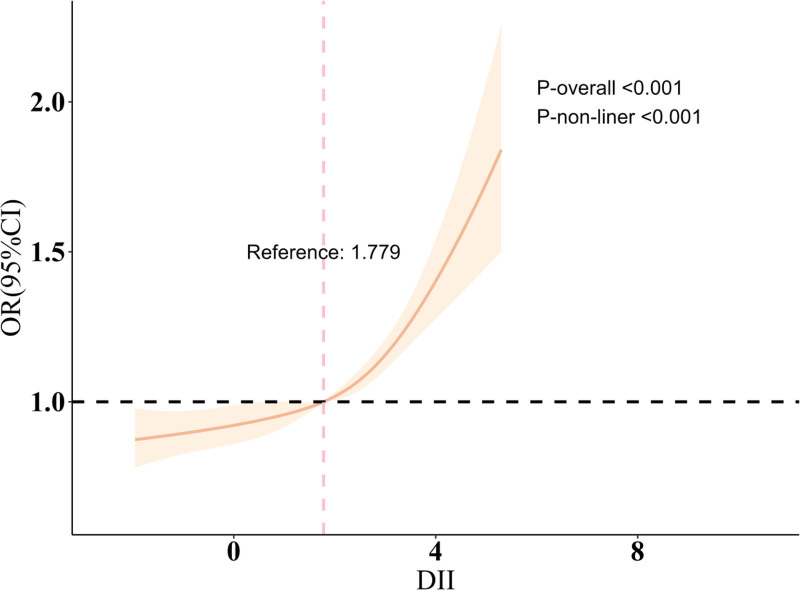
Restricted cubic spline (RCS) plot illustrating the nonlinear association between the dietary inflammatory index (DII) and the odds ratio (OR) for sleep disorders. The model was fully adjusted for all covariates. The solid line represents the estimated OR, and the shaded area indicates the 95% confidence interval. The vertical dashed line marks the reference DII value (1.779), beyond which the risk of sleep disorders increases sharply, suggesting a potential threshold effect. *P*-values indicate a significant overall and nonlinear association (*P*-overall < .001; *P*-nonlinear < .001).

In this observational analysis, we constructed 3 weighted models to examine the association between DII and sleep disorders (Table 2). The preliminary model showed a 22.6% increase in sleep disorder risk for each unit rise in DII (OR = 1.226, 95% CI: 1.166–1.289, *P* < .001). After adjusting for gender, age, and race in model 2, the risk increased by 20.3% (OR = 1.203, 95% CI: 1.144–1.266, *P* < .001). In the fully adjusted model, each unit increase in DII was linked to a 12.9% rise in sleep disorder risk (OR = 1.129, 95% CI: 1.079–1.182, *P* < .001). A similar dose–response was found across DII quartiles, with the highest quartile (Q4) showing a significantly greater risk than the lowest quartile (Q1) (OR = 1.423, 95% CI: 1.228–1.648, *P* < .001). These results suggest that higher DII values, reflecting increased dietary inflammation, are linked to greater sleep disorder risk. However, since this is an observational study, no causal inferences can be drawn. Trend tests confirmed that sleep disorder risk rose with higher DII levels (trend *P* < .001). Further analysis using a RCS model revealed a nonlinear association between DII and sleep disorders (*P*-overall < .001; *P*-nonlinear < .001). As shown in Figure 2, when the DII value is below approximately 1.779, the risk of sleep disorders remains relatively stable; however, once the DII exceeds this reference value, the odds ratio for sleep disorders increases sharply, suggesting a nonlinear threshold effect. This finding indicates that moderate to high levels of dietary inflammation may significantly increase the risk of sleep disorders, whereas lower levels of inflammation may have a limited impact. Additionally, the widening of confidence intervals at the extreme ends of the DII range suggests lower estimation precision in those regions.

**Table 2:** Association of DII and sleep disorder.

	Model 1		Model 2		Model 3	
	OR (95% CI)	*P*	OR (95% CI)	*P*	OR (95% CI)	*P*
DII	1.226 (1.166, 1.289)	<.001	1.203 (1.144, 1.266)	<.001	1.129 (1.079, 1.182)	<.001
Q1	Ref		Ref		Ref	
Q2	1.140 (0.964, 1.349)	.128	1.121 (0.949, 1.324)	.188	1.066 (0.901, 1.261)	.461
Q3	1.401 (1.212, 1.619)	<.001	1.351 (1.174, 1.555)	<.001	1.215 (1.059, 1.395)	.006
Q4	1.821 (1.559, 2.128)	<.001	1.718 (1.481, 1.992)	<.001	1.423 (1.228, 1.648)	<.001
*P* for trend		<.001		<.001		<.001

Model 1: no covariates were adjusted; Model 2: age, sex, and race were adjusted; Model 3: model 2 plus additional adjustment for the married/live with partner status, education level, poverty income ratios, smoking status, alcohol use, BMI, hypertension, and diabetes were adjusted.

DII = dietary inflammatory index, OR = odds ratio, ref = reference, 95% CI = 95% confidence interval.

*P* < .05 was considered statistically significant.

### 3.2. MR analysis

In contrast to the observational analysis in Section 3.1, this section explores the causal relationship between specific nutrients in the DII and sleep disorders using MR. MR utilizes genetic variants as IVs to examine causal effects, which helps mitigate confounding and reverse causality, common limitations in observational studies.

**Figure 3.**
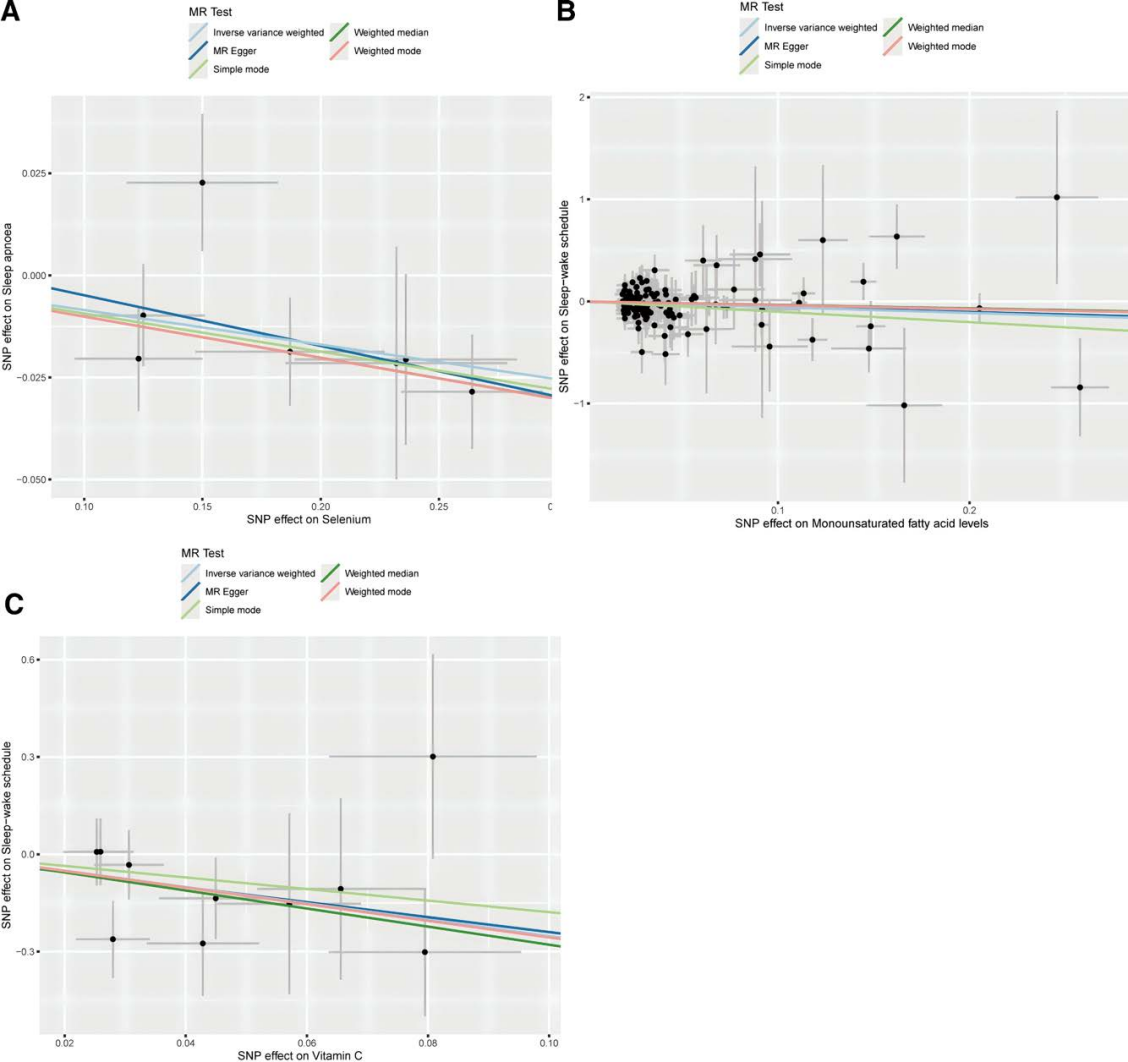
Presents scatter plots from Mendelian randomization (MR) analyses, assessing the impact of various nutrients on sleep-related conditions: (A) Effect of selenium on obstructive sleep apnea. (B) Effect of monounsaturated fatty acids on sleep–wake. (C) Effect of vitamin C on sleep–wake.

**Figure 4.**
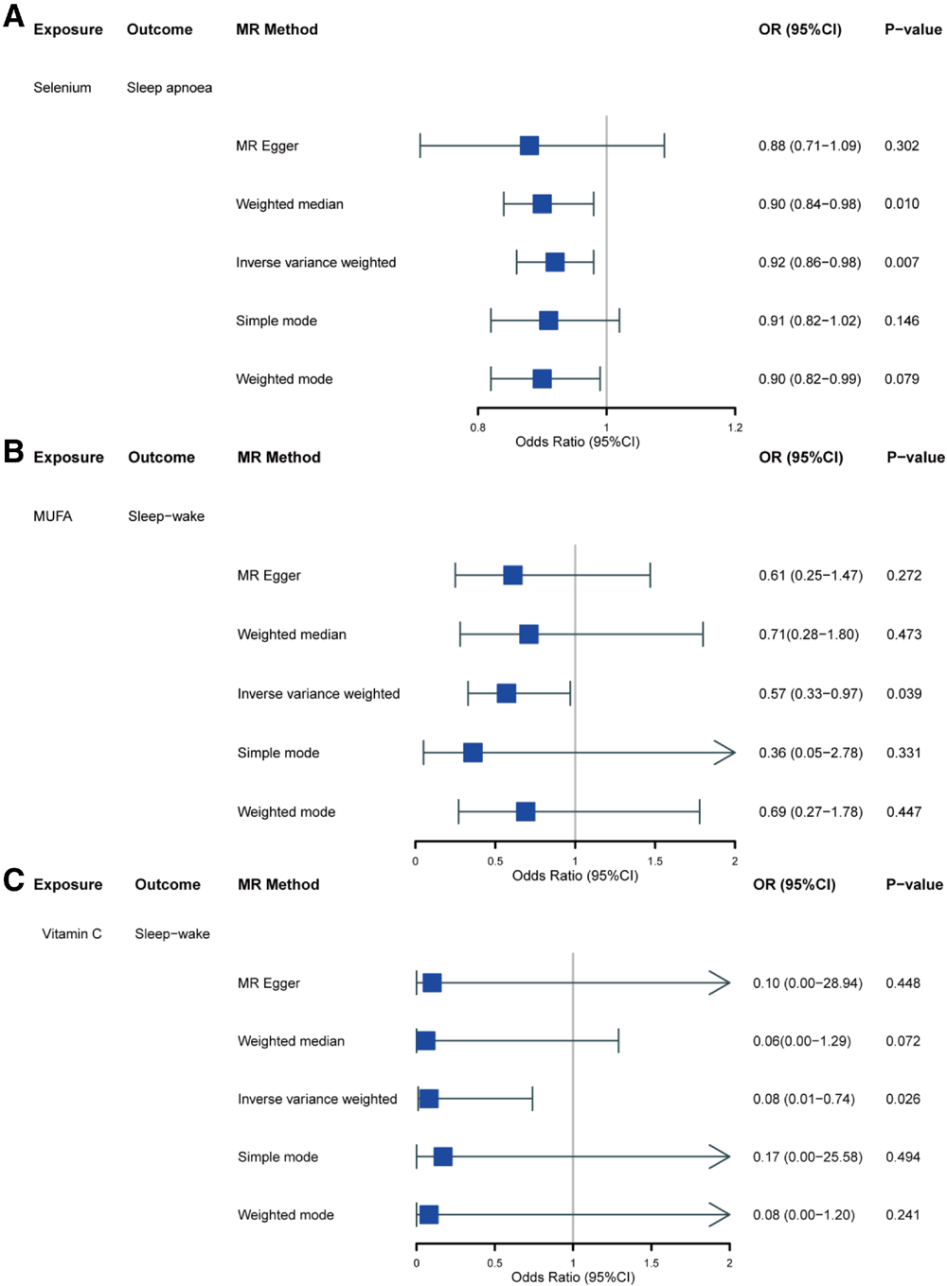
Forest plot for the causal effect of DII on the risk of sleep disorder derived from inverse-variance weighted. (A) Forest plot for the effect of selenium on sleep apnea. (B) Forest plot for the effect of MUFA on sleep–wake. (C) Forest plot for the effect of vitamin C on sleep–wake. OR = odds ratio, CI = confidence interval, MUFA = monounsaturated fatty acids.

Table S3, Supplemental Digital Content, https://links.lww.com/MD/P364 provides the IV summary for each nutrient in the DII. Each IV analyzed in this study exhibited *F*-statistics exceeding 10, indicating no significant weak instrument bias. Omega-3 and omega-6 fatty acids were excluded, with 26 nutrients analyzed. Initial IVW analysis suggested that genetically determined selenium is associated with a protective role in reducing sleep apnea (OR = 0.92, 95% CI: 0.86–0.98, *P* = .007). Similarly, genetically determined vitamin C and MUFAs demonstrated protective effects against sleep–wake disturbances (OR = 0.57, 95% CI: 0.33–0.97, *P* = .039) and (OR = 0.08, 95% CI: 0.01–0.74, *P* = .026), respectively (Figs. 3 and 4). Nevertheless, there was insufficient evidence to confirm causal links between DII components and other subgroups of sleep disorders, such as insomnia and hypersomnia. These results suggest that while certain nutrients, such as selenium, vitamin C, and MUFAs, appear to have causal effects on sleep disorders, further research is needed to confirm these relationships in other sleep disorder subtypes. To assess the robustness of these findings, we employed additional MR methods. The weighted median analysis, which is more robust to pleiotropy, corroborated the protective effect of selenium against sleep apnea (OR = 0.90, 95% CI: 0.84–0.98, *P* = .010). These findings were consistent across different MR methods, supporting the causal reliability of these relationships. Overall, the uniform trend across various MR methods further supports the causal relationship between selenium, vitamin C, and MUFAs and sleep disorders.

### 3.3. Sensitivity analysis

This study evaluated the effects of nutrients within DII on 4 sleep disorder subtypes. First, we applied the Cochran *Q* test to assess result heterogeneity, finding significant variations in the effects of protein, polyunsaturated fatty acids, alcoholic drinks, and iron on sleep apnoea. Additionally, vitamin B12 showed significant heterogeneity in relation to sleep–wake schedule, zinc to insomnia, and both zinc and vitamin C to hypersomnia; detailed data are provided in Table S4, Supplemental Digital Content, https://links.lww.com/MD/P364. Second, to evaluate potential pleiotropy bias, we utilized MR-Egger intercept and MR-PRESSO global tests. The MR-Egger intercept test results indicated no statistically significant difference from zero when the exposure was zero, providing evidence for the absence of pleiotropy in Table S5, Supplemental Digital Content, https://links.lww.com/MD/P364. Similarly, the MR-PRESSO global test results (all *P*-values > .05) supported the conclusion of no pleiotropy. Lastly, through leave-one-out sensitivity analysis, we observed that excluding any single single nucleotide polymorphism did not alter the risk estimates, further confirming the robustness of the study results.

Overall, the observational analysis based on NHANES data suggests a significant association between higher DII scores and increased risk of sleep disorders. However, the MR analysis further supports a causal relationship between specific nutrients, such as selenium, vitamin C, and MUFAs, and protection against sleep disorders. This combined approach allows us to assess both correlations and causal effects, providing a more comprehensive understanding of the role of diet in sleep health.

## 4. Discussion

This study employed a cross-sectional study and MR design to explore the link between the DII and sleep disorders, utilizing data from the 2007 to 2018 NHANES survey. We examined the association between inflammatory diets and sleep disorders, finding that those having with the highest DII were at a significantly greater risk of sleep disorders compared to those with lower DII. Trend analysis further confirmed that the probability of sleep disorders rose markedly with higher DII levels (trend *P* < .001). Subsequently, We performed additional adjustments using the RCS model and identified signs of a nonlinear association. Previous studies have shown that dietary patterns directly affect sleep quality and the occurrence of sleep disorders, independent of body weight and composition.^[25]^ As a crucial tool for assessing the potential inflammatory effects of diets, DII has been applied in numerous studies.^[7,8]^

There is increasing evidence in adult populations suggesting an connection between DII and sleep disorders. For example, a previous study demonstrated that DII scores correlated with sleep disorders and sleep duration among 30,121 U.S. adults, reinforcing the crucial impact of diet in the development of sleep disorders.^[26]^ Another study involving 249 Iranian students found a positive correlation between pro-inflammatory diets and impaired sleep quality, with the risk of poor sleep quality significantly increasing across higher DII quartiles.^[27]^ Similarly, Italian researchers led by Godos et al found through a survey based on the Pittsburgh Sleep Quality Index and food frequency questionnaire that elevated DII scores correlated with a reduced chance of achieving better sleep quality. Specifically, pro-inflammatory diets were linked to increased wake time after sleep onset and poorer sleep quality as measured by the Pittsburgh Sleep Quality Index.^[28]^ Additionally, a series of systematic reviews revealed a notable positive correlation between increased DII scores and sleep disorders.^[13]^ These findings are consistent with our results, emphasizing the important role of diet in the occurrence of sleep disorders. The inflammatory potential of diet represents a key area for both preventing and managing sleep disorders. Although the exact mechanisms remain unclear, current research suggests a cross-sectional association between pro-inflammatory diets and sleep disorders. A review on sleep and immune regulation describes the role of inflammatory peptides in the regulation of sleep homeostasis.^[5]^ Inflammatory cytokines such as IL-1 and pathogen-associated molecular patterns (e.g, lipopolysaccharides) influence multiple brain regions responsible for sleep regulation, such as the nucleus of the solitary tract and various hypothalamic nuclei, through the vagus nerve, thereby affecting sleep.^[29]^ Macrophage-like cells in the humoral pathway respond to pathogen-associated molecular patterns by expressing Toll-like receptors, producing inflammatory cytokines that can enter the brain through mechanisms such as volume diffusion, subsequently affecting sleep.^[30]^ Dynamic regulation of the blood–brain barrier (BBB): The BBB regulates the transport of immunomodulatory molecules through active transport mechanisms that are controlled by sleep and circadian rhythms. Infections and aging can alter BBB function, further altering inflammatory cytokine levels in the brain, which impacts sleep patterns.^[31]^ Activated immune cells (e.g., monocytes) can migrate into the brain’s vasculature and parenchyma. Inflammatory signals prompt microglia and astrocytes to produce chemokines, attracting more immune cells into the brain, which may alter sleep patterns.^[32]^ These interacting mechanisms explain the complex relationship between inflammatory states and sleep disorders, providing biological support for the research.

To delve deeper into the link between each nutrient in DII and sleep disorders, we explored the causal relationships between 26 nutrients and 4 subgroups of sleep disorders using the GWAS database. The IVW results showed that selenium has a protective effect on sleep apnea, while vitamin C and MUFAs have protective effects on sleep–wake disorders. These findings further validate the therapeutic potential of anti-inflammatory diets for sleep disorders. In an observational study, Grandner et al found that selenium and vitamin C intake were lower in people with sleep disorders than in those with normal sleep.^[33]^ This is consistent with our study’s conclusions. Selenium is an essential trace element with anti-inflammatory and antioxidant functions and is a core component of anti-inflammatory diets. Studies have shown that selenium can modulate inflammatory markers, decrease pro-inflammatory cytokines, and alleviate oxidative stress and inflammation, which are associated with sleep disorders. One study indicated that intravenous selenium administration can lower IL-1β and IL-6, key pro-inflammatory cytokines, attenuate inflammation, and significantly improve inflammatory conditions in patients.^[34]^ Another study suggested that selenium supplementation could improve sleep quality and reduce sleep problems caused by oxidative stress and inflammation.^[35]^ Moreover, selenium may impact the endocrine system, particularly hormones related to sleep such as melatonin and cortisol. By regulating these hormone levels, selenium may help synchronize circadian rhythms and improve sleep.^[36]^ Similarly, vitamin C is a crucial water-soluble nutrient primarily sourced from fruits and vegetables. It is widely recognized for its potent anti-inflammatory properties. Vitamin C significantly reduces the production of pro-inflammatory cytokines and free radicals, thereby effectively lowering inflammation and oxidative stress.^[37]^ Additionally, vitamin C could be vital in promoting restorative sleep.^[38]^ In sleep regulation, vitamin C acts as a key modulator in synthesizing neurotransmitters like dopamine and serotonin, which are crucial for mood and sleep patterns. Supplementing with vitamin C may have antidepressant effects, improve mood, and thereby alleviate insomnia.^[39]^ As the roles of selenium and vitamin C in improving sleep are increasingly recognized, MUFAs also play a similar role in a healthy diet. As an essential component of anti-inflammatory diets, MUFAs are thought to support sleep health by modulating the body’s inflammatory response. MUFAs exert anti-inflammatory effects on various organs such as the eyes, skin, lungs, liver, and gut by activating neutrophils.^[40]^ A 15-week study comparing mice fed diets high in MUFAs versus SFAs found that mice consuming MUFAs had elevated anti-inflammatory markers and reduced pro-inflammatory markers. This suggests that a MUFA-rich diet enhances anti-inflammatory responses and reduces inflammation.^[41]^ The combined effects of these 3 nutrients showed significant inflammation reduction and improvement in sleep quality, confirming the potential of anti-inflammatory diets in preventing and treating sleep disorders.

The relationship between inflammation and sleep disorders involves complex interactions of various biological mechanisms. From neurotransmission adjustments to cellular and molecular-level interactions, the inflammatory state affects neurotransmitter activity, modulates BBB function, and induces changes in immune cells in the central nervous system, collectively contributing to alterations in sleep patterns. These mechanisms not only demonstrate the direct link between inflammation and sleep quality and duration but also highlight the critical role of inflammation regulation in the prevention and treatment of sleep disorders. A deeper understanding of these interactions can inform targeted interventions to improve sleep quality in individuals affected by inflammation.

The main strength of this analysis lies in observational methods of data from the 2007 to 2018 NHANES, combined with MR methods. Our study utilized a large sample size and assessed various factors through multivariable regression models, effectively adjusting for multiple confounders and enhancing the statistical strength for evaluation the causal relationship between DII and sleep disorders. Additionally, the MR technique helps mitigate biases from unmeasured confounders and reverse causality. Notably, both methods produced highly consistent results, further strengthening the credibility of our conclusions.

However, this study has several limitations. First, the diagnosis of sleep disorders was based on self-reported data, which may lead to an underestimation of the true prevalence of these conditions due to recall bias or social desirability bias. Self-reports are inherently prone to inaccuracies as participants may not accurately recall their sleep disturbances. The DII assessments were based on retrospective 24-hour dietary recalls, which may not fully reflect long-term dietary habits or account for changes in diet over time. Furthermore, the majority of our data came from European and American populations, which limits the generalizability of the results to other ethnic groups and regions. Finally, while the MR analysis can provide strong evidence for causal inference, it is still subject to certain assumptions and biases. These include the potential for pleiotropy, where the instrumental variables may have effects on sleep disorders through pathways other than the DII, and population stratification, which may affect the external validity of the results.

## 5. Conclusion

In summary, this study reveals a nonlinear association between the DII and sleep disorders, supported by both observational data and MR analysis. While our findings suggest that a pro-inflammatory diet may increase the risk of sleep disorders and that certain nutrients such as selenium, vitamin C, and MUFAs may have protective effects, these results should be interpreted with caution.

To further validate the observed associations and clarify the causal pathways, future research should prioritize well-designed longitudinal studies and randomized controlled trials. These approaches are essential to assess the temporal relationships and evaluate the efficacy of anti-inflammatory dietary interventions in diverse populations.

Although our findings offer promising insights, the potential clinical implications of an anti-inflammatory diet in improving sleep health remain preliminary. More robust evidence is needed before making concrete dietary recommendations in clinical practice.

## Acknowledgments

We would like to express our sincere gratitude to the NHANES and IEU OpenGWAS databases for providing the invaluable data platforms. We also extend our thanks to the authors of the data, as well as the developers of the R software and associated packages, whose contributions were essential for the Mendelian randomization analysis presented in this article.

## Author contributions

**Conceptualization:** Junjie Jiang.

**Data curation:** Junjie Jiang.

**Formal analysis:** Junjie Jiang.

**Funding acquisition:** Junjie Jiang.

**Investigation:** Junjie Jiang, Shan Huang, Wei Yao.

**Methodology:** Junjie Jiang, Yi Yuan.

**Project administration:** Junjie Jiang.

**Resources:** Junjie Jiang.

**Software:** Junjie Jiang.

**Supervision:** Junjie Jiang, Tao Huang, Zhongfang Xia.

**Validation:** Junjie Jiang.

**Visualization:** Junjie Jiang.

**Writing – original draft:** Junjie Jiang.

**Writing – review & editing:** Junjie Jiang.

## Supplementary Material


