# Supplementary material for: Exploring the link between dietary inflammatory index and sleep disorders: Insights from NHANES and Mendelian randomization approach
Source: Medicine (Baltimore). 2025 Jul 4;104(27):e43170. doi: 10.1097/MD.0000000000043170 (PMC12237396; doi:10.1097/MD.0000000000043170)
Supplement: Supplementary file 1 [file medi-104-e43170-s001.docx]

**Table S1** Diagnostic criteria of covariates in NHANES

| Variable | Diagnostic criteria |
| --- | --- |
| Age | The oldest age in the population is just 80 years old, and the age of education is required to be greater than or equal to 20  years old, so 20-80 is divided into three age groups, namely 20-40, 41-60, and 61-80. |
| Gender | This variable was subdivided into two groups: female and male. |
| Race | Populations or ethnic groups are categorized into four distinct segments: Mexican Americans, non-Hispanic whites, non  Hispanic blacks, and other races. |
| Marital status: | In categorizing marital status, the amalgamation of marriage and cohabitation into a single category is contrasted with the  distinct classification of being single,divorced, widowed, or separated. |
| Annual household income | The poverty-income ratio was delineated as≥1.3 for non-poverty status and<1.3 for poverty status. Each participant had  three to four consecutive blood pressure levels taken. |
| Education level | Educational attainment is categorized as either incomplete or complete with respect to upper secondary education. |
| Body mass index (BMI) | Obesity was defned as a BMI≥30 according to Centers for Disease Control and Prevention (CDC) guidelines. |
| Smoke status | The assessment of smoking status is based on the question: "Smoked at least 100 cigarettes in life?" Respondents who  answer "yes" are considered smokers. Otherwise, they are considered non-smokers. |
| Alcohol consumption | Alcohol status was ascertained based on participants’ responses to the query, which inquired whether they had  consumed 12 or more alcoholic drinks in the preceding year. Participants who responded afrmatively were  classifed as alcohol users, and conversely for those who answered negatively |
| Diabetes mellitus(DM) | The diagnostic criteria for diabetes are: the doctor told you that you have diabetes, or glycohemoglobin HbA1c (%) greater  than 6.5, or use of diabetes medication or insulin. Participants who responded "yes" were classified as having diabetes. |
| Hypertension | Each participant had three to four consecutive blood pressure levels taken. Systolic (SBP) and diastolic blood pressure  (DBP) values were computed as the average of all extant readings. Hypertension was identified when SBP was ≥140  mmHg and DBP was ≥90 mmHg. |

**Table S2** GWAS Summary Information for the Dietary Inflammatory Index (DII) and Its Association with Sleep Disorders

| Trait | Build | Year | Author | Sample size | Dataset | Population |
| --- | --- | --- | --- | --- | --- | --- |
| Carbohydrate | HG19/GRCh37 | 2018 | Ben Elsworth | 64,979 | ukb-b-7244 | European |
| Serum total protein level | HG19/GRCh37 | 2021 | Barton AR | 400,482 | ebi-a-GCST90025995 | European |
| Total fatty acid levels | HG19/GRCh37 | 2022 | Richardson TG | 115,006 | ebi-a-GCST90092987 | European |
| Carotene | HG19/GRCh37 | 2018 | Ben Elsworth | 64,979 | ukb-b-16202 | European |
| Folate | HG19/GRCh37 | 2018 | Ben Elsworth | 64,979 | ukb-b-11349 | European |
| Iron | HG19/GRCh37 | 2018 | Ben Elsworth | 64,979 | ukb-b-20447 | European |
| Magnesium | HG19/GRCh37 | 2018 | Ben Elsworth | 64,979 | ukb-b-7372 | European |
| Selenium | HG19/GRCh37 | 2013 | Evans | 2,874 | ieu-a-1075 | European |
| Vitamin A(Retinol) | HG19/GRCh37 | 2018 | Ben Elsworth | 62,991 | ukb-b-17406 | European |
| Vitamin B1 | HG19/GRCh37 | 2018 | Sun BB | 3,301 | prot-a-3084 | European |
| Vitamin B2(Riboflavin) | HG19/GRCh37 | 2018 | Sun BB | 3,301 | prot-a-2529 | European |
| Vitamin B3(Nicotinamide riboside) | HG19/GRCh37 | 2021 | Panyard DJ | 291 | ebi-a-GCST90026238 | European |
| Vitamin B6 | HG19/GRCh37 | 2018 | Ben Elsworth | 64,979 | ukb-b-7864 | European |
| Vitamin B12 | HG19/GRCh37 | 2018 | Ben Elsworth | 64,979 | ukb-b-19524 | European |
| Vitamin C | HG19/GRCh37 | 2018 | Ben Elsworth | 64,979 | ukb-b-19390 | European |
| Vitamin D | HG19/GRCh37 | 2018 | Ben Elsworth | 64,979 | ukb-b-18593 | European |
| Vitamin E | HG19/GRCh37 | 2018 | Ben Elsworth | 64,979 | ukb-b-6888 | European |
| Zinc | HG19/GRCh37 | 2013 | Evans | 2,603 | ieu-a-1079 | European |
| Saturated fatty acid levels | HG19/GRCh37 | 2022 | Richardson TG | 115,006 | ebi-a-GCST90092980 | European |
| Polyunsaturated fatty acid levels | HG19/GRCh37 | 2022 | Richardson TG | 115,006 | ebi-a-GCST90092939 | European |
| Monounsaturated fatty acid levels | HG19/GRCh37 | 2022 | Richardson TG | 115,006 | ebi-a-GCST90092928 | European |
| Englyst dietary fibre | HG19/GRCh37 | 2018 | Ben Elsworth | 64,979 | ukb-b-19085 | European |
| Total cholesterol levels | HG19/GRCh37 | 2021 | Barton AR | 437,878 | ebi-a-GCST90025953 | European |
| Omega-3 fatty acids | HG19/GRCh37 | 2020 | Borges CM | 114,999 | met-d-Omega_3 | European |
| Omega-6 fatty acids | HG19/GRCh37 | 2020 | Borges CM | 114,999 | met-d-Omega_6 | European |
| Caffeine levels | HG19/GRCh37 | 2021 | Panyard DJ | 291 | ebi-a-GCST90026134 | European |
| Energy | HG19/GRCh37 | 2018 | Ben Elsworth | 64,979 | ukb-b-7323 | European |
| Alcoholic drinks | HG19/GRCh37 | 2019 | Liu, M | 335,394 | ieu-b-73 | European |
| Sleep apnoea | HG19/GRCh37 | 2021 | NA | 16,761 case/201,194 control | finn-b-G6_SLEEPAPNO | European |
| Sleep wake | HG19/GRCh37 | 2021 | NA | 190 case/216,164 control | finn-b-F5_SLEEPWAKE | European |
| Hypersomnia | HG19/GRCh37 | 2021 | NA | 176 case/216,164 control | finn-b-F5_HYPERSOMNIA | European |
| Insomnia | HG19/GRCh37 | 2021 | NA | 1,691 case/216,164 control | finn-b-F5_INSOMNIA | European |

**Table S3** Genetic instrumental variables for the Dietary Inflammatory Index (DII)

| Trait | Chr | SNP | Effect allele | Other allele | Eaf | Beta | SE | *P* | F |
| --- | --- | --- | --- | --- | --- | --- | --- | --- | --- |
| Carbohydrate | 2 | rs112137399 | T | C | 0.089 | 0.050 | 0.010 | 0.000 | 26.293 |
| Carbohydrate | 2 | rs11693885 | A | G | 0.445 | -0.025 | 0.005 | 0.000 | 21.395 |
| Carbohydrate | 2 | rs546217 | C | A | 0.430 | 0.026 | 0.005 | 0.000 | 22.062 |
| Carbohydrate | 3 | rs12715219 | T | C | 0.546 | -0.025 | 0.005 | 0.000 | 21.983 |
| Carbohydrate | 3 | rs148088494 | G | T | 0.014 | -0.117 | 0.024 | 0.000 | 22.860 |
| Carbohydrate | 4 | rs2647238 | C | T | 0.587 | -0.025 | 0.005 | 0.000 | 20.908 |
| Carbohydrate | 4 | rs58370602 | T | C | 0.015 | 0.106 | 0.022 | 0.000 | 22.358 |
| Carbohydrate | 4 | rs11132733 | C | T | 0.816 | -0.032 | 0.007 | 0.000 | 21.538 |
| Carbohydrate | 5 | rs35237101 | G | A | 0.042 | 0.070 | 0.014 | 0.000 | 25.720 |
| Carbohydrate | 5 | rs62347998 | T | C | 0.042 | 0.069 | 0.014 | 0.000 | 24.908 |
| Carbohydrate | 6 | rs9399996 | C | G | 0.160 | 0.036 | 0.007 | 0.000 | 24.446 |
| Carbohydrate | 7 | rs7803193 | G | C | 0.072 | 0.049 | 0.011 | 0.000 | 21.808 |
| Carbohydrate | 9 | rs68133983 | C | G | 0.066 | 0.050 | 0.011 | 0.000 | 21.261 |
| Carbohydrate | 11 | rs11224098 | G | A | 0.144 | -0.037 | 0.008 | 0.000 | 23.343 |
| Carbohydrate | 12 | rs59150700 | A | G | 0.019 | -0.093 | 0.020 | 0.000 | 21.506 |
| Carbohydrate | 14 | rs3185777 | C | T | 0.711 | 0.027 | 0.006 | 0.000 | 21.425 |
| Carbohydrate | 17 | rs6416839 | C | A | 0.346 | -0.027 | 0.006 | 0.000 | 23.193 |
| Carbohydrate | 20 | rs6089697 | A | G | 0.466 | -0.025 | 0.005 | 0.000 | 21.020 |
| Carbohydrate | 21 | rs13052873 | A | G | 0.223 | -0.032 | 0.006 | 0.000 | 24.186 |
| Protein | 1 | rs12132507 | G | A | 0.426 | -0.010 | 0.002 | 0.000 | 21.556 |
| Protein | 1 | rs10802190 | T | A | 0.104 | -0.016 | 0.003 | 0.000 | 20.502 |
| Protein | 1 | rs165316 | G | A | 0.197 | -0.017 | 0.003 | 0.000 | 43.908 |
| Protein | 1 | rs75484590 | A | C | 0.030 | 0.031 | 0.006 | 0.000 | 24.162 |
| Protein | 1 | rs4987358 | T | G | 0.270 | 0.015 | 0.002 | 0.000 | 39.949 |
| Protein | 1 | rs1805415 | C | T | 0.843 | -0.014 | 0.003 | 0.000 | 22.355 |
| Protein | 1 | rs12239046 | C | T | 0.627 | 0.011 | 0.002 | 0.000 | 23.452 |
| Protein | 1 | rs17849502 | T | G | 0.052 | 0.033 | 0.005 | 0.000 | 45.492 |
| Protein | 1 | rs9430347 | C | T | 0.790 | 0.018 | 0.003 | 0.000 | 42.592 |
| Protein | 1 | rs7535528 | A | G | 0.367 | 0.014 | 0.002 | 0.000 | 39.408 |
| Protein | 1 | rs6656611 | C | T | 0.603 | 0.012 | 0.002 | 0.000 | 33.343 |
| Protein | 1 | rs12128213 | G | A | 0.506 | -0.012 | 0.002 | 0.000 | 32.425 |
| Protein | 1 | rs2745953 | T | A | 0.291 | 0.017 | 0.002 | 0.000 | 50.967 |
| Protein | 1 | rs12130314 | T | G | 0.276 | -0.013 | 0.002 | 0.000 | 28.383 |
| Protein | 1 | rs2244588 | A | G | 0.911 | 0.031 | 0.004 | 0.000 | 69.980 |
| Protein | 1 | rs4970834 | T | C | 0.186 | -0.023 | 0.003 | 0.000 | 68.927 |
| Protein | 1 | rs484959 | C | T | 0.536 | -0.020 | 0.002 | 0.000 | 89.505 |
| Protein | 1 | rs11204744 | G | A | 0.256 | 0.022 | 0.002 | 0.000 | 80.608 |
| Protein | 1 | rs75657969 | A | G | 0.054 | -0.024 | 0.005 | 0.000 | 25.279 |
| Protein | 1 | rs3170660 | C | T | 0.605 | 0.012 | 0.002 | 0.000 | 28.528 |
| Protein | 1 | rs1276300 | A | G | 0.727 | 0.013 | 0.002 | 0.000 | 29.578 |
| Protein | 1 | rs7532966 | C | T | 0.499 | 0.009 | 0.002 | 0.000 | 20.285 |
| Protein | 1 | rs1046934 | C | A | 0.342 | 0.011 | 0.002 | 0.000 | 25.004 |
| Protein | 1 | rs16826069 | G | A | 0.212 | -0.022 | 0.003 | 0.000 | 70.069 |
| Protein | 1 | rs55781203 | G | A | 0.325 | -0.013 | 0.002 | 0.000 | 31.571 |
| Protein | 1 | rs61804211 | T | G | 0.105 | -0.127 | 0.003 | 0.000 | 1372.075 |
| Protein | 1 | rs3737623 | C | T | 0.392 | -0.017 | 0.002 | 0.000 | 65.405 |
| Protein | 1 | rs4131826 | C | T | 0.364 | 0.017 | 0.002 | 0.000 | 57.912 |
| Protein | 1 | rs1689800 | G | A | 0.359 | -0.018 | 0.002 | 0.000 | 67.455 |
| Protein | 1 | rs1065489 | T | G | 0.171 | -0.021 | 0.003 | 0.000 | 56.487 |
| Protein | 1 | rs61830291 | C | A | 0.096 | 0.029 | 0.004 | 0.000 | 65.646 |
| Protein | 1 | rs2991974 | G | A | 0.430 | 0.013 | 0.002 | 0.000 | 35.604 |
| Protein | 1 | rs4846567 | T | G | 0.297 | -0.019 | 0.002 | 0.000 | 70.240 |
| Protein | 1 | rs11249164 | A | C | 0.387 | 0.012 | 0.002 | 0.000 | 32.637 |
| Protein | 1 | rs10903122 | G | A | 0.502 | -0.034 | 0.002 | 0.000 | 257.731 |
| Protein | 1 | rs1194587 | T | C | 0.456 | -0.021 | 0.002 | 0.000 | 98.633 |
| Protein | 1 | rs10798269 | G | A | 0.638 | 0.010 | 0.002 | 0.000 | 21.704 |
| Protein | 1 | rs2275603 | G | A | 0.208 | -0.037 | 0.003 | 0.000 | 203.108 |
| Protein | 1 | rs6683067 | C | T | 0.166 | -0.015 | 0.003 | 0.000 | 30.011 |
| Protein | 2 | rs11553951 | T | C | 0.281 | 0.011 | 0.002 | 0.000 | 21.477 |
| Protein | 2 | rs2307358 | C | T | 0.160 | -0.023 | 0.003 | 0.000 | 60.512 |
| Protein | 2 | rs13385731 | C | T | 0.071 | -0.037 | 0.004 | 0.000 | 79.702 |
| Protein | 2 | rs4321376 | T | C | 0.215 | 0.013 | 0.003 | 0.000 | 27.078 |
| Protein | 2 | rs62128989 | G | A | 0.506 | -0.010 | 0.002 | 0.000 | 21.288 |
| Protein | 2 | rs72820479 | G | A | 0.175 | -0.018 | 0.003 | 0.000 | 41.671 |
| Protein | 2 | rs58941251 | T | C | 0.108 | 0.018 | 0.003 | 0.000 | 29.427 |
| Protein | 2 | rs10198628 | G | A | 0.532 | 0.014 | 0.002 | 0.000 | 41.783 |
| Protein | 2 | rs2972143 | G | A | 0.647 | 0.016 | 0.002 | 0.000 | 51.011 |
| Protein | 2 | rs12991516 | A | G | 0.475 | 0.010 | 0.002 | 0.000 | 21.283 |
| Protein | 2 | rs10865035 | G | A | 0.527 | -0.027 | 0.002 | 0.000 | 164.063 |
| Protein | 2 | rs2256422 | T | C | 0.327 | -0.026 | 0.002 | 0.000 | 130.517 |
| Protein | 2 | rs8207 | G | A | 0.265 | 0.013 | 0.002 | 0.000 | 30.826 |
| Protein | 2 | rs2954959 | G | A | 0.282 | -0.011 | 0.002 | 0.000 | 20.937 |
| Protein | 2 | rs1260326 | C | T | 0.598 | -0.044 | 0.002 | 0.000 | 414.069 |
| Protein | 2 | rs11124671 | A | G | 0.839 | 0.021 | 0.003 | 0.000 | 51.706 |
| Protein | 2 | rs116447416 | G | A | 0.035 | -0.028 | 0.006 | 0.000 | 23.283 |
| Protein | 2 | rs7595905 | T | C | 0.560 | -0.010 | 0.002 | 0.000 | 23.762 |
| Protein | 2 | rs1509495 | T | C | 0.655 | -0.023 | 0.002 | 0.000 | 108.667 |
| Protein | 2 | rs2252662 | C | T | 0.610 | -0.018 | 0.002 | 0.000 | 72.486 |
| Protein | 2 | rs3087243 | A | G | 0.451 | -0.021 | 0.002 | 0.000 | 97.807 |
| Protein | 2 | rs56390510 | C | G | 0.176 | -0.015 | 0.003 | 0.000 | 28.709 |
| Protein | 2 | rs1047891 | A | C | 0.313 | -0.024 | 0.002 | 0.000 | 108.185 |
| Protein | 2 | rs2002444 | G | A | 0.147 | -0.015 | 0.003 | 0.000 | 23.486 |
| Protein | 2 | rs9333568 | G | C | 0.049 | -0.026 | 0.005 | 0.000 | 28.844 |
| Protein | 2 | rs1050951 | A | G | 0.123 | -0.019 | 0.003 | 0.000 | 34.999 |
| Protein | 2 | rs1834748 | C | T | 0.540 | 0.017 | 0.002 | 0.000 | 64.520 |
| Protein | 2 | rs6722472 | C | G | 0.168 | -0.022 | 0.003 | 0.000 | 64.356 |
| Protein | 2 | rs72907747 | A | G | 0.076 | 0.017 | 0.004 | 0.000 | 18.221 |
| Protein | 2 | rs9333289 | C | T | 0.277 | 0.011 | 0.002 | 0.000 | 20.310 |
| Protein | 2 | rs56265609 | C | T | 0.028 | 0.030 | 0.006 | 0.000 | 21.819 |
| Protein | 2 | rs11126986 | G | A | 0.516 | 0.020 | 0.002 | 0.000 | 87.867 |
| Protein | 2 | rs1128249 | T | G | 0.392 | -0.016 | 0.002 | 0.000 | 57.466 |
| Protein | 3 | rs11712226 | C | T | 0.381 | -0.012 | 0.002 | 0.000 | 28.353 |
| Protein | 3 | rs13085674 | A | G | 0.318 | 0.013 | 0.002 | 0.000 | 33.914 |
| Protein | 3 | rs11709020 | T | C | 0.460 | -0.013 | 0.002 | 0.000 | 38.652 |
| Protein | 3 | rs9869047 | A | G | 0.231 | -0.014 | 0.003 | 0.000 | 32.006 |
| Protein | 3 | rs1915092 | A | T | 0.435 | -0.011 | 0.002 | 0.000 | 25.109 |
| Protein | 3 | rs13077912 | G | A | 0.146 | -0.016 | 0.003 | 0.000 | 29.748 |
| Protein | 3 | rs61345811 | T | C | 0.261 | 0.016 | 0.002 | 0.000 | 46.068 |
| Protein | 3 | rs9866046 | A | G | 0.075 | 0.019 | 0.004 | 0.000 | 21.788 |
| Protein | 3 | rs9819371 | T | C | 0.065 | -0.054 | 0.004 | 0.000 | 161.214 |
| Protein | 3 | rs34330560 | A | G | 0.674 | -0.010 | 0.002 | 0.000 | 20.490 |
| Protein | 3 | rs2334230 | T | G | 0.433 | 0.011 | 0.002 | 0.000 | 25.730 |
| Protein | 3 | rs2371108 | T | G | 0.387 | 0.012 | 0.002 | 0.000 | 32.415 |
| Protein | 3 | rs12486526 | G | A | 0.221 | 0.013 | 0.003 | 0.000 | 22.812 |
| Protein | 3 | rs7635636 | A | C | 0.210 | -0.014 | 0.003 | 0.000 | 29.167 |
| Protein | 3 | rs7629936 | A | G | 0.244 | 0.017 | 0.002 | 0.000 | 46.495 |
| Protein | 3 | rs9845762 | T | C | 0.709 | 0.015 | 0.002 | 0.000 | 44.116 |
| Protein | 3 | rs12632656 | G | A | 0.280 | -0.013 | 0.002 | 0.000 | 28.808 |
| Protein | 3 | rs17036326 | G | A | 0.121 | -0.041 | 0.003 | 0.000 | 161.425 |
| Protein | 3 | rs62258127 | A | G | 0.373 | -0.030 | 0.002 | 0.000 | 190.627 |
| Protein | 3 | rs9843148 | G | A | 0.711 | -0.011 | 0.002 | 0.000 | 20.954 |
| Protein | 3 | rs5001409 | C | A | 0.389 | -0.025 | 0.002 | 0.000 | 129.592 |
| Protein | 3 | rs7652761 | T | C | 0.193 | 0.015 | 0.003 | 0.000 | 32.763 |
| Protein | 3 | rs10936600 | T | A | 0.243 | 0.018 | 0.002 | 0.000 | 49.752 |
| Protein | 3 | rs900400 | C | T | 0.400 | 0.024 | 0.002 | 0.000 | 125.945 |
| Protein | 3 | rs17266090 | G | A | 0.439 | -0.013 | 0.002 | 0.000 | 34.846 |
| Protein | 3 | rs571391 | A | G | 0.653 | -0.014 | 0.002 | 0.000 | 39.667 |
| Protein | 4 | rs5743618 | A | C | 0.231 | -0.019 | 0.003 | 0.000 | 56.244 |
| Protein | 4 | rs16844401 | A | G | 0.065 | 0.048 | 0.004 | 0.000 | 122.405 |
| Protein | 4 | rs149914551 | A | C | 0.035 | -0.049 | 0.006 | 0.000 | 69.605 |
| Protein | 4 | rs114303452 | G | A | 0.012 | 0.073 | 0.010 | 0.000 | 56.558 |
| Protein | 4 | rs6823013 | T | C | 0.111 | 0.017 | 0.003 | 0.000 | 25.704 |
| Protein | 4 | rs28590233 | T | C | 0.197 | 0.016 | 0.003 | 0.000 | 37.663 |
| Protein | 4 | rs11737560 | T | C | 0.124 | -0.029 | 0.003 | 0.000 | 83.583 |
| Protein | 4 | rs73245721 | T | C | 0.031 | 0.035 | 0.006 | 0.000 | 32.669 |
| Protein | 4 | rs6847288 | C | T | 0.868 | -0.017 | 0.003 | 0.000 | 28.296 |
| Protein | 4 | rs6839825 | G | A | 0.635 | 0.014 | 0.002 | 0.000 | 39.909 |
| Protein | 4 | rs17063777 | A | G | 0.057 | -0.026 | 0.005 | 0.000 | 32.884 |
| Protein | 4 | rs4610379 | C | T | 0.308 | -0.011 | 0.002 | 0.000 | 21.312 |
| Protein | 4 | rs3804404 | A | G | 0.737 | -0.016 | 0.002 | 0.000 | 41.737 |
| Protein | 4 | rs71620317 | C | G | 0.398 | -0.018 | 0.002 | 0.000 | 71.418 |
| Protein | 4 | rs7676237 | A | G | 0.337 | 0.012 | 0.002 | 0.000 | 30.036 |
| Protein | 4 | rs56371872 | T | C | 0.026 | -0.034 | 0.007 | 0.000 | 26.999 |
| Protein | 4 | rs1585213 | T | C | 0.399 | -0.030 | 0.002 | 0.000 | 196.445 |
| Protein | 4 | rs6840938 | A | G | 0.424 | -0.016 | 0.002 | 0.000 | 57.599 |
| Protein | 4 | rs77849807 | G | A | 0.016 | 0.117 | 0.009 | 0.000 | 189.166 |
| Protein | 4 | rs111739466 | A | G | 0.048 | 0.039 | 0.005 | 0.000 | 64.147 |
| Protein | 4 | rs4031150 | G | A | 0.732 | -0.012 | 0.002 | 0.000 | 26.626 |
| Protein | 4 | rs2271807 | G | A | 0.083 | 0.018 | 0.004 | 0.000 | 21.180 |
| Protein | 4 | rs79595315 | C | G | 0.036 | -0.025 | 0.006 | 0.000 | 20.090 |
| Protein | 4 | rs77704934 | G | A | 0.156 | -0.015 | 0.003 | 0.000 | 26.779 |
| Protein | 4 | rs10516487 | A | G | 0.311 | -0.023 | 0.002 | 0.000 | 103.594 |
| Protein | 4 | rs13114759 | A | G | 0.224 | -0.017 | 0.003 | 0.000 | 41.537 |
| Protein | 4 | rs185354376 | A | G | 0.031 | 0.030 | 0.006 | 0.000 | 22.710 |
| Protein | 4 | rs76553165 | T | G | 0.102 | -0.018 | 0.003 | 0.000 | 25.216 |
| Protein | 4 | rs4691380 | T | C | 0.325 | -0.013 | 0.002 | 0.000 | 31.919 |
| Protein | 5 | rs7720261 | T | G | 0.772 | 0.015 | 0.003 | 0.000 | 36.032 |
| Protein | 5 | rs3805433 | G | C | 0.273 | 0.013 | 0.002 | 0.000 | 27.817 |
| Protein | 5 | rs116010824 | T | G | 0.027 | 0.038 | 0.006 | 0.000 | 34.972 |
| Protein | 5 | rs10069690 | T | C | 0.258 | -0.021 | 0.002 | 0.000 | 72.571 |
| Protein | 5 | rs4496694 | T | C | 0.629 | 0.012 | 0.002 | 0.000 | 31.711 |
| Protein | 5 | rs34592828 | A | G | 0.045 | -0.053 | 0.005 | 0.000 | 105.490 |
| Protein | 5 | rs9314162 | A | C | 0.266 | 0.029 | 0.002 | 0.000 | 142.295 |
| Protein | 5 | rs34651 | T | C | 0.916 | 0.024 | 0.004 | 0.000 | 39.507 |
| Protein | 5 | rs6890853 | A | G | 0.280 | -0.013 | 0.002 | 0.000 | 28.662 |
| Protein | 5 | rs158607 | A | T | 0.201 | -0.012 | 0.003 | 0.000 | 21.379 |
| Protein | 5 | rs245055 | G | A | 0.267 | 0.014 | 0.002 | 0.000 | 32.536 |
| Protein | 5 | rs67199213 | G | A | 0.261 | 0.014 | 0.002 | 0.000 | 32.713 |
| Protein | 5 | rs4705762 | T | C | 0.511 | -0.011 | 0.002 | 0.000 | 28.556 |
| Protein | 5 | rs2303719 | G | T | 0.703 | 0.012 | 0.002 | 0.000 | 28.155 |
| Protein | 5 | rs778582 | T | C | 0.289 | -0.014 | 0.002 | 0.000 | 37.442 |
| Protein | 5 | rs3909469 | G | A | 0.256 | 0.019 | 0.002 | 0.000 | 61.610 |
| Protein | 5 | rs34183525 | G | C | 0.346 | -0.010 | 0.002 | 0.000 | 18.156 |
| Protein | 5 | rs1499280 | A | C | 0.916 | 0.022 | 0.004 | 0.000 | 32.714 |
| Protein | 5 | rs27300 | T | C | 0.535 | -0.010 | 0.002 | 0.000 | 23.221 |
| Protein | 5 | rs10900855 | T | C | 0.223 | -0.013 | 0.003 | 0.000 | 26.340 |
| Protein | 5 | rs36694 | A | C | 0.742 | 0.019 | 0.002 | 0.000 | 60.261 |
| Protein | 5 | rs12516449 | A | G | 0.671 | 0.011 | 0.002 | 0.000 | 25.422 |
| Protein | 5 | rs6859219 | A | C | 0.205 | -0.038 | 0.003 | 0.000 | 211.244 |
| Protein | 5 | rs11954893 | G | A | 0.204 | 0.015 | 0.003 | 0.000 | 31.809 |
| Protein | 6 | rs62441844 | T | C | 0.205 | 0.015 | 0.003 | 0.000 | 31.814 |
| Protein | 6 | rs3093023 | A | G | 0.433 | 0.016 | 0.002 | 0.000 | 53.272 |
| Protein | 6 | rs12195797 | A | G | 0.039 | 0.041 | 0.005 | 0.000 | 58.145 |
| Protein | 6 | rs3812195 | G | A | 0.356 | -0.013 | 0.002 | 0.000 | 34.595 |
| Protein | 6 | rs79441819 | T | G | 0.020 | -0.040 | 0.007 | 0.000 | 28.112 |
| Protein | 6 | rs7768393 | A | G | 0.166 | 0.015 | 0.003 | 0.000 | 28.411 |
| Protein | 6 | rs3757114 | C | A | 0.474 | -0.014 | 0.002 | 0.000 | 42.531 |
| Protein | 6 | rs72815053 | C | A | 0.175 | -0.014 | 0.003 | 0.000 | 24.152 |
| Protein | 6 | rs3129858 | A | G | 0.303 | -0.034 | 0.003 | 0.000 | 97.349 |
| Protein | 6 | rs29001568 | A | G | 0.474 | -0.054 | 0.003 | 0.000 | 265.197 |
| Protein | 6 | rs7767987 | C | T | 0.342 | 0.012 | 0.002 | 0.000 | 28.368 |
| Protein | 6 | rs2281389 | G | A | 0.171 | 0.031 | 0.003 | 0.000 | 111.144 |
| Protein | 6 | rs727979 | T | C | 0.164 | 0.023 | 0.003 | 0.000 | 62.090 |
| Protein | 6 | rs55772024 | A | G | 0.244 | -0.017 | 0.002 | 0.000 | 48.291 |
| Protein | 6 | rs7759938 | T | C | 0.678 | -0.012 | 0.002 | 0.000 | 25.879 |
| Protein | 6 | rs1800562 | A | G | 0.077 | -0.045 | 0.004 | 0.000 | 110.744 |
| Protein | 6 | rs1033180 | T | C | 0.072 | -0.045 | 0.004 | 0.000 | 118.985 |
| Protein | 6 | rs55666429 | A | G | 0.586 | -0.023 | 0.002 | 0.000 | 115.241 |
| Protein | 6 | rs10499197 | G | T | 0.031 | 0.049 | 0.006 | 0.000 | 64.680 |
| Protein | 6 | rs3756772 | T | C | 0.400 | 0.021 | 0.002 | 0.000 | 95.174 |
| Protein | 6 | rs1847472 | A | C | 0.353 | 0.027 | 0.002 | 0.000 | 146.390 |
| Protein | 6 | rs6927569 | C | T | 0.523 | -0.009 | 0.002 | 0.000 | 19.985 |
| Protein | 6 | rs9320820 | T | G | 0.640 | 0.013 | 0.002 | 0.000 | 36.306 |
| Protein | 6 | rs78950083 | C | T | 0.018 | -0.058 | 0.011 | 0.000 | 28.330 |
| Protein | 6 | rs17710008 | A | G | 0.183 | 0.013 | 0.003 | 0.000 | 22.768 |
| Protein | 6 | rs490010 | G | A | 0.505 | -0.014 | 0.002 | 0.000 | 46.184 |
| Protein | 6 | rs12209143 | A | G | 0.146 | 0.016 | 0.003 | 0.000 | 29.951 |
| Protein | 7 | rs157934 | C | T | 0.305 | -0.020 | 0.002 | 0.000 | 72.146 |
| Protein | 7 | rs7782699 | T | C | 0.115 | 0.026 | 0.003 | 0.000 | 60.477 |
| Protein | 7 | rs4726163 | T | C | 0.481 | 0.010 | 0.002 | 0.000 | 23.348 |
| Protein | 7 | rs2906176 | C | T | 0.644 | 0.015 | 0.002 | 0.000 | 48.677 |
| Protein | 7 | rs34058374 | A | G | 0.514 | -0.014 | 0.002 | 0.000 | 46.200 |
| Protein | 7 | rs4722771 | A | G | 0.441 | -0.010 | 0.002 | 0.000 | 24.279 |
| Protein | 7 | rs386956 | T | C | 0.641 | -0.012 | 0.002 | 0.000 | 27.730 |
| Protein | 7 | rs55714084 | A | C | 0.340 | -0.011 | 0.002 | 0.000 | 22.355 |
| Protein | 7 | rs1049742 | T | C | 0.079 | 0.024 | 0.004 | 0.000 | 35.455 |
| Protein | 7 | rs849335 | C | T | 0.657 | -0.012 | 0.002 | 0.000 | 27.514 |
| Protein | 7 | rs2855726 | A | G | 0.330 | 0.015 | 0.002 | 0.000 | 44.915 |
| Protein | 7 | rs38249 | G | A | 0.744 | -0.016 | 0.002 | 0.000 | 42.524 |
| Protein | 7 | rs38832 | T | C | 0.831 | -0.014 | 0.003 | 0.000 | 25.477 |
| Protein | 7 | rs221794 | C | T | 0.837 | -0.016 | 0.003 | 0.000 | 30.076 |
| Protein | 7 | rs4291144 | A | T | 0.192 | 0.016 | 0.003 | 0.000 | 35.991 |
| Protein | 7 | rs6968865 | T | A | 0.628 | 0.013 | 0.002 | 0.000 | 36.065 |
| Protein | 7 | rs66768935 | G | A | 0.438 | -0.011 | 0.002 | 0.000 | 27.718 |
| Protein | 7 | rs17551629 | G | A | 0.064 | 0.022 | 0.004 | 0.000 | 24.876 |
| Protein | 7 | rs4917014 | G | T | 0.319 | -0.027 | 0.002 | 0.000 | 138.221 |
| Protein | 7 | rs10951010 | A | G | 0.224 | 0.013 | 0.003 | 0.000 | 27.385 |
| Protein | 7 | rs6852 | A | C | 0.519 | 0.010 | 0.002 | 0.000 | 20.326 |
| Protein | 7 | rs6464102 | G | T | 0.636 | 0.013 | 0.002 | 0.000 | 37.439 |
| Protein | 7 | rs339054 | G | T | 0.505 | -0.012 | 0.002 | 0.000 | 31.200 |
| Protein | 7 | rs2004640 | T | G | 0.514 | 0.010 | 0.002 | 0.000 | 24.100 |
| Protein | 7 | rs77713358 | T | G | 0.037 | -0.034 | 0.006 | 0.000 | 36.224 |
| Protein | 8 | rs11777360 | C | A | 0.162 | 0.014 | 0.003 | 0.000 | 24.388 |
| Protein | 8 | rs2720659 | A | G | 0.343 | -0.015 | 0.002 | 0.000 | 47.840 |
| Protein | 8 | rs3824107 | A | G | 0.236 | -0.017 | 0.002 | 0.000 | 46.315 |
| Protein | 8 | rs1347320 | G | A | 0.633 | 0.013 | 0.002 | 0.000 | 36.170 |
| Protein | 8 | rs13259910 | T | C | 0.150 | -0.018 | 0.003 | 0.000 | 36.037 |
| Protein | 8 | rs1023767 | A | G | 0.235 | 0.020 | 0.002 | 0.000 | 62.726 |
| Protein | 8 | rs16887244 | G | A | 0.251 | 0.016 | 0.002 | 0.000 | 43.874 |
| Protein | 8 | rs6984883 | G | C | 0.583 | 0.019 | 0.002 | 0.000 | 79.064 |
| Protein | 8 | rs6470368 | T | C | 0.293 | -0.013 | 0.002 | 0.000 | 33.459 |
| Protein | 8 | rs2875973 | T | C | 0.371 | 0.018 | 0.002 | 0.000 | 66.587 |
| Protein | 8 | rs4341142 | C | T | 0.156 | -0.016 | 0.003 | 0.000 | 31.491 |
| Protein | 8 | rs6557616 | C | G | 0.786 | 0.016 | 0.003 | 0.000 | 37.583 |
| Protein | 8 | rs4873265 | A | G | 0.949 | 0.029 | 0.005 | 0.000 | 36.926 |
| Protein | 8 | rs60714144 | T | C | 0.400 | 0.013 | 0.002 | 0.000 | 37.856 |
| Protein | 8 | rs55978020 | A | C | 0.208 | 0.018 | 0.003 | 0.000 | 40.651 |
| Protein | 8 | rs4841132 | G | A | 0.909 | 0.057 | 0.004 | 0.000 | 210.605 |
| Protein | 8 | rs2280104 | C | T | 0.647 | -0.016 | 0.002 | 0.000 | 53.026 |
| Protein | 8 | rs2942194 | G | A | 0.281 | 0.010 | 0.002 | 0.000 | 18.133 |
| Protein | 8 | rs76457973 | T | C | 0.026 | 0.032 | 0.007 | 0.000 | 24.024 |
| Protein | 8 | rs10097731 | T | G | 0.146 | 0.022 | 0.003 | 0.000 | 54.208 |
| Protein | 9 | rs3793524 | G | C | 0.344 | -0.015 | 0.002 | 0.000 | 44.245 |
| Protein | 9 | rs697449 | T | G | 0.188 | -0.016 | 0.003 | 0.000 | 35.921 |
| Protein | 9 | rs290989 | C | T | 0.470 | -0.018 | 0.002 | 0.000 | 75.456 |
| Protein | 9 | rs10975962 | C | A | 0.452 | 0.010 | 0.002 | 0.000 | 21.788 |
| Protein | 9 | rs10811675 | A | G | 0.461 | -0.011 | 0.002 | 0.000 | 24.927 |
| Protein | 9 | rs7874594 | G | T | 0.304 | -0.010 | 0.002 | 0.000 | 20.368 |
| Protein | 9 | rs2871401 | C | A | 0.487 | -0.014 | 0.002 | 0.000 | 45.666 |
| Protein | 9 | rs296894 | T | G | 0.197 | 0.027 | 0.003 | 0.000 | 100.808 |
| Protein | 9 | rs643434 | A | G | 0.341 | 0.018 | 0.002 | 0.000 | 65.197 |
| Protein | 9 | rs17810013 | G | A | 0.179 | -0.017 | 0.003 | 0.000 | 38.948 |
| Protein | 9 | rs66477686 | C | T | 0.259 | -0.022 | 0.002 | 0.000 | 81.657 |
| Protein | 9 | rs6475582 | T | C | 0.099 | -0.022 | 0.004 | 0.000 | 38.965 |
| Protein | 9 | rs11557154 | T | C | 0.127 | -0.023 | 0.003 | 0.000 | 50.056 |
| Protein | 9 | rs12350531 | C | G | 0.678 | 0.012 | 0.002 | 0.000 | 27.674 |
| Protein | 9 | rs7870753 | G | A | 0.221 | -0.013 | 0.003 | 0.000 | 27.618 |
| Protein | 10 | rs56278466 | G | T | 0.662 | 0.028 | 0.002 | 0.000 | 162.404 |
| Protein | 10 | rs1926739 | A | G | 0.486 | -0.015 | 0.002 | 0.000 | 46.764 |
| Protein | 10 | rs2278308 | T | G | 0.513 | -0.013 | 0.002 | 0.000 | 34.896 |
| Protein | 10 | rs12255289 | T | C | 0.229 | 0.014 | 0.003 | 0.000 | 29.889 |
| Protein | 10 | rs2068888 | A | G | 0.451 | -0.017 | 0.002 | 0.000 | 60.227 |
| Protein | 10 | rs11008183 | T | C | 0.603 | -0.010 | 0.002 | 0.000 | 23.516 |
| Protein | 10 | rs6583833 | T | C | 0.510 | 0.010 | 0.002 | 0.000 | 22.190 |
| Protein | 10 | rs4387287 | C | A | 0.842 | 0.016 | 0.003 | 0.000 | 32.013 |
| Protein | 10 | rs10885531 | T | C | 0.497 | 0.010 | 0.002 | 0.000 | 21.059 |
| Protein | 10 | rs3849969 | G | A | 0.704 | 0.017 | 0.002 | 0.000 | 49.197 |
| Protein | 10 | rs10748652 | A | T | 0.571 | -0.012 | 0.002 | 0.000 | 32.457 |
| Protein | 10 | rs3793707 | T | G | 0.212 | -0.013 | 0.003 | 0.000 | 26.490 |
| Protein | 10 | rs1864393 | G | A | 0.175 | 0.016 | 0.003 | 0.000 | 33.975 |
| Protein | 10 | rs12778662 | T | C | 0.086 | 0.032 | 0.004 | 0.000 | 71.727 |
| Protein | 10 | rs196197 | T | A | 0.459 | 0.016 | 0.002 | 0.000 | 53.978 |
| Protein | 10 | rs1848797 | G | A | 0.398 | -0.015 | 0.002 | 0.000 | 49.939 |
| Protein | 11 | rs7104821 | G | A | 0.501 | 0.016 | 0.002 | 0.000 | 60.368 |
| Protein | 11 | rs17120523 | G | A | 0.059 | -0.026 | 0.004 | 0.000 | 34.206 |
| Protein | 11 | rs1313288 | C | T | 0.477 | -0.011 | 0.002 | 0.000 | 26.513 |
| Protein | 11 | rs79499090 | T | G | 0.019 | 0.051 | 0.008 | 0.000 | 43.663 |
| Protein | 11 | rs6590334 | C | T | 0.524 | 0.017 | 0.002 | 0.000 | 62.693 |
| Protein | 11 | rs174533 | A | G | 0.344 | 0.034 | 0.002 | 0.000 | 237.130 |
| Protein | 11 | rs7118017 | G | A | 0.468 | 0.011 | 0.002 | 0.000 | 27.093 |
| Protein | 11 | rs3740688 | T | G | 0.542 | 0.020 | 0.002 | 0.000 | 87.490 |
| Protein | 11 | rs10789825 | G | A | 0.692 | 0.029 | 0.002 | 0.000 | 157.747 |
| Protein | 11 | rs907611 | A | G | 0.315 | -0.012 | 0.002 | 0.000 | 26.265 |
| Protein | 11 | rs34481144 | T | C | 0.492 | 0.013 | 0.002 | 0.000 | 39.182 |
| Protein | 11 | rs1151496 | G | A | 0.272 | -0.020 | 0.002 | 0.000 | 68.471 |
| Protein | 11 | rs11021537 | A | G | 0.039 | -0.033 | 0.005 | 0.000 | 36.202 |
| Protein | 11 | rs4909945 | C | T | 0.688 | 0.016 | 0.002 | 0.000 | 48.307 |
| Protein | 11 | rs76360685 | T | C | 0.524 | -0.012 | 0.002 | 0.000 | 28.902 |
| Protein | 11 | rs636406 | G | C | 0.359 | -0.010 | 0.002 | 0.000 | 20.404 |
| Protein | 11 | rs7941030 | C | T | 0.384 | 0.020 | 0.002 | 0.000 | 87.954 |
| Protein | 11 | rs7115200 | G | T | 0.439 | -0.018 | 0.002 | 0.000 | 68.777 |
| Protein | 11 | rs7935829 | G | A | 0.397 | -0.011 | 0.002 | 0.000 | 26.271 |
| Protein | 11 | rs72903909 | C | T | 0.086 | 0.019 | 0.004 | 0.000 | 24.514 |
| Protein | 12 | rs837499 | G | A | 0.858 | 0.025 | 0.003 | 0.000 | 68.512 |
| Protein | 12 | rs1401881 | G | A | 0.223 | -0.018 | 0.003 | 0.000 | 49.348 |
| Protein | 12 | rs11168250 | T | G | 0.239 | -0.013 | 0.002 | 0.000 | 29.072 |
| Protein | 12 | rs1800973 | A | C | 0.061 | 0.021 | 0.004 | 0.000 | 21.764 |
| Protein | 12 | rs34322 | C | T | 0.523 | -0.014 | 0.002 | 0.000 | 42.346 |
| Protein | 12 | rs187995827 | T | C | 0.022 | -0.037 | 0.007 | 0.000 | 25.328 |
| Protein | 12 | rs4963756 | A | G | 0.265 | 0.018 | 0.002 | 0.000 | 55.867 |
| Protein | 12 | rs11181073 | G | A | 0.407 | -0.010 | 0.002 | 0.000 | 21.819 |
| Protein | 12 | rs2229357 | A | G | 0.240 | -0.015 | 0.002 | 0.000 | 36.215 |
| Protein | 12 | rs35961650 | G | C | 0.085 | -0.019 | 0.004 | 0.000 | 25.921 |
| Protein | 12 | rs12820562 | C | T | 0.104 | 0.018 | 0.003 | 0.000 | 26.648 |
| Protein | 12 | rs7310149 | T | G | 0.835 | -0.015 | 0.003 | 0.000 | 28.254 |
| Protein | 12 | rs36004829 | A | G | 0.034 | 0.028 | 0.006 | 0.000 | 22.764 |
| Protein | 12 | rs76191003 | T | C | 0.068 | 0.023 | 0.004 | 0.000 | 29.351 |
| Protein | 12 | rs2293446 | A | G | 0.381 | -0.015 | 0.002 | 0.000 | 48.014 |
| Protein | 12 | rs3184504 | C | T | 0.515 | -0.019 | 0.002 | 0.000 | 80.325 |
| Protein | 12 | rs4149081 | A | G | 0.162 | -0.015 | 0.003 | 0.000 | 28.400 |
| Protein | 12 | rs2289957 | A | T | 0.530 | -0.009 | 0.002 | 0.000 | 18.290 |
| Protein | 12 | rs76785029 | T | C | 0.083 | -0.020 | 0.004 | 0.000 | 27.316 |
| Protein | 12 | rs1196883 | C | T | 0.566 | -0.015 | 0.002 | 0.000 | 48.588 |
| Protein | 12 | rs6539083 | G | A | 0.498 | 0.011 | 0.002 | 0.000 | 25.326 |
| Protein | 12 | rs1471839 | T | C | 0.580 | 0.010 | 0.002 | 0.000 | 21.358 |
| Protein | 13 | rs56031211 | A | G | 0.182 | -0.017 | 0.003 | 0.000 | 40.483 |
| Protein | 13 | rs35383320 | A | G | 0.052 | -0.022 | 0.005 | 0.000 | 21.451 |
| Protein | 13 | rs9599885 | A | G | 0.306 | -0.016 | 0.002 | 0.000 | 47.911 |
| Protein | 13 | rs2329164 | A | G | 0.254 | 0.013 | 0.002 | 0.000 | 26.556 |
| Protein | 13 | rs7996996 | A | G | 0.386 | 0.014 | 0.002 | 0.000 | 41.366 |
| Protein | 13 | rs9600993 | C | A | 0.113 | 0.016 | 0.003 | 0.000 | 23.404 |
| Protein | 13 | rs12874404 | G | A | 0.054 | 0.054 | 0.005 | 0.000 | 132.584 |
| Protein | 13 | rs79490353 | C | T | 0.025 | 0.076 | 0.007 | 0.000 | 126.784 |
| Protein | 13 | rs2776959 | T | C | 0.837 | 0.015 | 0.003 | 0.000 | 28.773 |
| Protein | 13 | rs9521732 | A | C | 0.383 | -0.019 | 0.002 | 0.000 | 76.810 |
| Protein | 13 | rs1317573 | T | C | 0.604 | -0.010 | 0.002 | 0.000 | 21.457 |
| Protein | 13 | rs3742321 | C | T | 0.224 | -0.013 | 0.003 | 0.000 | 26.208 |
| Protein | 14 | rs66597987 | C | T | 0.169 | -0.017 | 0.003 | 0.000 | 36.165 |
| Protein | 14 | rs45581733 | A | G | 0.038 | 0.043 | 0.006 | 0.000 | 56.090 |
| Protein | 14 | rs8017377 | A | G | 0.470 | 0.017 | 0.002 | 0.000 | 61.097 |
| Protein | 14 | rs72731554 | A | T | 0.193 | 0.014 | 0.003 | 0.000 | 27.145 |
| Protein | 14 | rs28929474 | T | C | 0.020 | 0.147 | 0.008 | 0.000 | 368.548 |
| Protein | 14 | rs8904 | A | G | 0.362 | 0.017 | 0.002 | 0.000 | 57.486 |
| Protein | 14 | rs4983425 | A | G | 0.964 | -0.033 | 0.006 | 0.000 | 32.827 |
| Protein | 14 | rs112597211 | C | T | 0.137 | -0.023 | 0.003 | 0.000 | 54.791 |
| Protein | 14 | rs2015407 | G | A | 0.650 | -0.046 | 0.002 | 0.000 | 431.448 |
| Protein | 14 | rs3210043 | A | C | 0.161 | -0.021 | 0.003 | 0.000 | 50.905 |
| Protein | 14 | rs12881869 | T | C | 0.069 | -0.032 | 0.004 | 0.000 | 57.052 |
| Protein | 14 | rs72713373 | C | T | 0.058 | -0.040 | 0.005 | 0.000 | 76.408 |
| Protein | 14 | rs17620990 | C | T | 0.287 | -0.017 | 0.002 | 0.000 | 55.267 |
| Protein | 15 | rs11630901 | C | T | 0.186 | -0.013 | 0.003 | 0.000 | 22.808 |
| Protein | 15 | rs74010640 | G | A | 0.231 | -0.015 | 0.003 | 0.000 | 33.282 |
| Protein | 15 | rs339969 | A | C | 0.614 | 0.022 | 0.002 | 0.000 | 103.307 |
| Protein | 15 | rs10152591 | C | A | 0.103 | -0.031 | 0.003 | 0.000 | 78.421 |
| Protein | 15 | rs12909292 | T | G | 0.318 | 0.012 | 0.002 | 0.000 | 29.751 |
| Protein | 15 | rs7495616 | G | C | 0.752 | -0.013 | 0.002 | 0.000 | 25.895 |
| Protein | 15 | rs11630691 | C | T | 0.264 | 0.013 | 0.002 | 0.000 | 29.810 |
| Protein | 15 | rs2046356 | A | G | 0.886 | -0.019 | 0.003 | 0.000 | 32.067 |
| Protein | 15 | rs16973688 | T | C | 0.213 | -0.024 | 0.003 | 0.000 | 88.272 |
| Protein | 15 | rs55707100 | T | C | 0.026 | 0.101 | 0.007 | 0.000 | 226.612 |
| Protein | 15 | rs6496717 | T | C | 0.760 | 0.015 | 0.002 | 0.000 | 36.766 |
| Protein | 16 | rs3812987 | A | C | 0.226 | -0.018 | 0.003 | 0.000 | 47.134 |
| Protein | 16 | rs2965827 | A | C | 0.538 | 0.017 | 0.002 | 0.000 | 67.705 |
| Protein | 16 | rs1800718 | C | A | 0.210 | -0.015 | 0.003 | 0.000 | 33.358 |
| Protein | 16 | rs7200786 | G | A | 0.548 | 0.017 | 0.002 | 0.000 | 61.727 |
| Protein | 16 | rs4985155 | G | A | 0.319 | 0.014 | 0.002 | 0.000 | 38.175 |
| Protein | 16 | rs7187776 | G | A | 0.398 | 0.016 | 0.002 | 0.000 | 53.281 |
| Protein | 16 | rs56059702 | T | G | 0.570 | 0.011 | 0.002 | 0.000 | 27.583 |
| Protein | 16 | rs11548656 | G | A | 0.036 | 0.032 | 0.006 | 0.000 | 30.585 |
| Protein | 16 | rs6565176 | C | T | 0.513 | 0.010 | 0.002 | 0.000 | 23.082 |
| Protein | 16 | rs16952251 | G | A | 0.314 | -0.013 | 0.002 | 0.000 | 30.991 |
| Protein | 16 | rs7203742 | T | C | 0.129 | -0.023 | 0.003 | 0.000 | 51.070 |
| Protein | 16 | rs9922891 | A | G | 0.073 | 0.021 | 0.004 | 0.000 | 26.518 |
| Protein | 16 | rs2581318 | T | G | 0.611 | 0.010 | 0.002 | 0.000 | 20.961 |
| Protein | 16 | rs7200879 | G | A | 0.241 | 0.021 | 0.002 | 0.000 | 73.672 |
| Protein | 17 | rs878829 | C | T | 0.130 | -0.019 | 0.003 | 0.000 | 37.887 |
| Protein | 17 | rs2232486 | C | T | 0.744 | -0.023 | 0.002 | 0.000 | 86.625 |
| Protein | 17 | rs41444548 | G | C | 0.075 | 0.047 | 0.004 | 0.000 | 137.357 |
| Protein | 17 | rs112876941 | T | A | 0.033 | 0.058 | 0.006 | 0.000 | 95.611 |
| Protein | 17 | rs11368 | G | A | 0.432 | 0.030 | 0.002 | 0.000 | 195.160 |
| Protein | 17 | rs77542162 | G | A | 0.023 | -0.097 | 0.007 | 0.000 | 178.587 |
| Protein | 17 | rs11078597 | C | T | 0.186 | 0.040 | 0.003 | 0.000 | 220.908 |
| Protein | 17 | rs3744015 | C | T | 0.327 | -0.016 | 0.002 | 0.000 | 50.742 |
| Protein | 17 | rs871014 | C | T | 0.496 | -0.012 | 0.002 | 0.000 | 33.743 |
| Protein | 17 | rs3803800 | G | A | 0.781 | -0.044 | 0.003 | 0.000 | 272.505 |
| Protein | 17 | rs11658072 | T | C | 0.626 | 0.017 | 0.002 | 0.000 | 55.717 |
| Protein | 17 | rs34562254 | A | G | 0.099 | 0.112 | 0.004 | 0.000 | 991.695 |
| Protein | 17 | rs4309436 | C | T | 0.316 | 0.024 | 0.002 | 0.000 | 108.075 |
| Protein | 17 | rs7498 | A | G | 0.235 | 0.012 | 0.003 | 0.000 | 21.486 |
| Protein | 17 | rs75242405 | T | C | 0.225 | -0.035 | 0.003 | 0.000 | 191.185 |
| Protein | 18 | rs55861534 | A | G | 0.218 | 0.013 | 0.003 | 0.000 | 24.319 |
| Protein | 18 | rs2406196 | C | T | 0.553 | 0.010 | 0.002 | 0.000 | 23.953 |
| Protein | 18 | rs1652376 | T | G | 0.463 | -0.011 | 0.002 | 0.000 | 24.408 |
| Protein | 18 | rs273726 | C | T | 0.448 | -0.010 | 0.002 | 0.000 | 21.778 |
| Protein | 18 | rs9953900 | T | C | 0.687 | 0.017 | 0.002 | 0.000 | 58.718 |
| Protein | 18 | rs28444870 | T | G | 0.285 | 0.016 | 0.002 | 0.000 | 45.903 |
| Protein | 18 | rs55711819 | C | A | 0.184 | -0.015 | 0.003 | 0.000 | 29.528 |
| Protein | 18 | rs117521417 | T | C | 0.014 | -0.045 | 0.009 | 0.000 | 24.786 |
| Protein | 18 | rs17070779 | C | T | 0.128 | 0.040 | 0.003 | 0.000 | 154.662 |
| Protein | 18 | rs74847855 | G | A | 0.037 | 0.029 | 0.006 | 0.000 | 27.034 |
| Protein | 19 | rs731839 | A | G | 0.665 | -0.016 | 0.002 | 0.000 | 53.898 |
| Protein | 19 | rs58895965 | A | C | 0.173 | 0.050 | 0.003 | 0.000 | 310.190 |
| Protein | 19 | rs12975589 | C | A | 0.437 | 0.011 | 0.002 | 0.000 | 26.588 |
| Protein | 19 | rs36084354 | A | G | 0.091 | -0.037 | 0.004 | 0.000 | 99.478 |
| Protein | 19 | rs10412176 | C | T | 0.799 | -0.014 | 0.003 | 0.000 | 29.016 |
| Protein | 19 | rs503279 | C | T | 0.534 | 0.035 | 0.002 | 0.000 | 273.949 |
| Protein | 19 | rs1982075 | G | C | 0.651 | -0.019 | 0.002 | 0.000 | 69.994 |
| Protein | 19 | rs2157849 | A | G | 0.319 | -0.015 | 0.002 | 0.000 | 45.826 |
| Protein | 19 | rs74438527 | A | G | 0.027 | 0.057 | 0.006 | 0.000 | 78.627 |
| Protein | 19 | rs141826060 | A | C | 0.081 | 0.019 | 0.004 | 0.000 | 24.744 |
| Protein | 19 | rs3745477 | G | T | 0.716 | -0.016 | 0.002 | 0.000 | 39.641 |
| Protein | 19 | rs8105687 | G | A | 0.673 | 0.014 | 0.002 | 0.000 | 36.936 |
| Protein | 19 | rs10419198 | T | C | 0.252 | -0.073 | 0.002 | 0.000 | 893.314 |
| Protein | 19 | rs3745318 | C | T | 0.749 | -0.013 | 0.002 | 0.000 | 27.068 |
| Protein | 19 | rs752878 | T | G | 0.167 | -0.013 | 0.003 | 0.000 | 19.908 |
| Protein | 19 | rs10409301 | C | T | 0.944 | 0.025 | 0.005 | 0.000 | 29.266 |
| Protein | 19 | rs10853751 | A | G | 0.600 | -0.014 | 0.002 | 0.000 | 43.742 |
| Protein | 20 | rs41278240 | T | C | 0.113 | 0.031 | 0.003 | 0.000 | 86.327 |
| Protein | 20 | rs2618610 | T | C | 0.052 | 0.023 | 0.005 | 0.000 | 22.374 |
| Protein | 20 | rs2427536 | A | G | 0.914 | 0.029 | 0.004 | 0.000 | 54.976 |
| Protein | 20 | rs73117925 | T | C | 0.065 | -0.020 | 0.004 | 0.000 | 21.867 |
| Protein | 20 | rs13043825 | T | C | 0.270 | -0.013 | 0.002 | 0.000 | 28.084 |
| Protein | 20 | rs184691263 | C | G | 0.198 | 0.019 | 0.003 | 0.000 | 48.536 |
| Protein | 20 | rs6124298 | A | G | 0.294 | 0.012 | 0.002 | 0.000 | 25.498 |
| Protein | 20 | rs76500013 | T | G | 0.067 | -0.026 | 0.004 | 0.000 | 35.704 |
| Protein | 20 | rs6080761 | A | G | 0.420 | 0.013 | 0.002 | 0.000 | 34.817 |
| Protein | 20 | rs291671 | A | G | 0.892 | 0.024 | 0.003 | 0.000 | 51.396 |
| Protein | 20 | rs2207132 | A | G | 0.034 | 0.035 | 0.006 | 0.000 | 34.711 |
| Protein | 21 | rs2823990 | A | G | 0.678 | 0.011 | 0.002 | 0.000 | 22.784 |
| Protein | 21 | rs2833801 | C | T | 0.420 | 0.011 | 0.002 | 0.000 | 24.010 |
| Protein | 22 | rs62222328 | A | G | 0.254 | -0.016 | 0.002 | 0.000 | 42.351 |
| Protein | 22 | rs4633 | T | C | 0.514 | 0.010 | 0.002 | 0.000 | 22.996 |
| Protein | 22 | rs9616381 | A | G | 0.183 | -0.014 | 0.003 | 0.000 | 25.970 |
| Protein | 22 | rs138950 | A | G | 0.124 | -0.016 | 0.003 | 0.000 | 25.364 |
| Protein | 22 | rs12537 | T | C | 0.361 | -0.024 | 0.002 | 0.000 | 121.735 |
| Protein | 22 | rs132642 | T | A | 0.830 | 0.016 | 0.003 | 0.000 | 33.209 |
| Protein | 22 | rs7287486 | T | G | 0.025 | -0.053 | 0.007 | 0.000 | 60.136 |
| Protein | 22 | rs62228872 | A | G | 0.080 | -0.018 | 0.004 | 0.000 | 21.927 |
| Protein | 22 | rs4822488 | T | G | 0.209 | 0.014 | 0.003 | 0.000 | 29.455 |
| Protein | 22 | rs45587437 | T | C | 0.142 | 0.016 | 0.003 | 0.000 | 27.479 |
| Total fat | 1 | rs187607506 | T | C | 0.245 | -0.023 | 0.005 | 0.000 | 21.738 |
| Total fat | 1 | rs660240 | C | T | 0.785 | 0.041 | 0.005 | 0.000 | 67.246 |
| Total fat | 1 | rs1002687 | A | G | 0.645 | 0.096 | 0.004 | 0.000 | 510.319 |
| Total fat | 1 | rs822928 | C | A | 0.530 | 0.024 | 0.004 | 0.000 | 34.190 |
| Total fat | 1 | rs534417 | G | A | 0.875 | 0.041 | 0.006 | 0.000 | 44.949 |
| Total fat | 1 | rs4846335 | A | C | 0.104 | 0.032 | 0.007 | 0.000 | 22.819 |
| Total fat | 2 | rs35757519 | C | T | 0.447 | 0.021 | 0.004 | 0.000 | 24.902 |
| Total fat | 2 | rs62183700 | C | G | 0.329 | 0.021 | 0.004 | 0.000 | 23.826 |
| Total fat | 2 | rs1877712 | A | G | 0.561 | -0.020 | 0.004 | 0.000 | 24.929 |
| Total fat | 2 | rs1260326 | C | T | 0.604 | -0.106 | 0.004 | 0.000 | 646.832 |
| Total fat | 2 | rs150913811 | G | A | 0.013 | 0.084 | 0.018 | 0.000 | 21.289 |
| Total fat | 2 | rs11895352 | T | C | 0.475 | -0.031 | 0.004 | 0.000 | 59.107 |
| Total fat | 2 | rs60960031 | A | G | 0.403 | -0.028 | 0.004 | 0.000 | 45.805 |
| Total fat | 2 | rs1128249 | T | G | 0.392 | -0.029 | 0.004 | 0.000 | 47.492 |
| Total fat | 2 | rs4299376 | T | G | 0.676 | -0.024 | 0.004 | 0.000 | 29.776 |
| Total fat | 2 | rs4564803 | T | G | 0.228 | -0.062 | 0.005 | 0.000 | 162.312 |
| Total fat | 3 | rs55921103 | T | G | 0.648 | 0.020 | 0.004 | 0.000 | 21.690 |
| Total fat | 3 | rs4683708 | T | C | 0.533 | -0.020 | 0.004 | 0.000 | 22.947 |
| Total fat | 3 | rs900048 | T | C | 0.710 | 0.027 | 0.004 | 0.000 | 35.326 |
| Total fat | 3 | rs11925442 | C | T | 0.124 | 0.029 | 0.006 | 0.000 | 21.622 |
| Total fat | 3 | rs13059141 | A | G | 0.113 | -0.030 | 0.006 | 0.000 | 21.738 |
| Total fat | 3 | rs7610260 | T | A | 0.678 | -0.021 | 0.004 | 0.000 | 23.008 |
| Total fat | 4 | rs1471251 | T | A | 0.397 | 0.026 | 0.004 | 0.000 | 39.845 |
| Total fat | 4 | rs2035816 | G | A | 0.084 | -0.037 | 0.007 | 0.000 | 25.250 |
| Total fat | 4 | rs34670304 | A | G | 0.553 | 0.020 | 0.004 | 0.000 | 23.964 |
| Total fat | 4 | rs4860948 | A | T | 0.244 | 0.028 | 0.005 | 0.000 | 33.985 |
| Total fat | 4 | rs13108218 | G | A | 0.615 | -0.035 | 0.004 | 0.000 | 70.040 |
| Total fat | 4 | rs11940694 | G | A | 0.605 | 0.022 | 0.004 | 0.000 | 27.426 |
| Total fat | 5 | rs3936511 | G | A | 0.192 | 0.034 | 0.005 | 0.000 | 43.930 |
| Total fat | 5 | rs4704834 | G | A | 0.644 | 0.045 | 0.004 | 0.000 | 110.243 |
| Total fat | 5 | rs3846663 | T | C | 0.374 | 0.032 | 0.004 | 0.000 | 58.057 |
| Total fat | 5 | rs35764948 | A | T | 0.055 | 0.043 | 0.009 | 0.000 | 23.642 |
| Total fat | 5 | rs6898411 | A | G | 0.054 | -0.042 | 0.009 | 0.000 | 21.235 |
| Total fat | 6 | rs9388530 | T | A | 0.436 | -0.019 | 0.004 | 0.000 | 21.448 |
| Total fat | 6 | rs28383314 | C | T | 0.623 | 0.043 | 0.004 | 0.000 | 107.129 |
| Total fat | 6 | rs72972355 | C | T | 0.098 | 0.033 | 0.007 | 0.000 | 22.387 |
| Total fat | 6 | rs12055389 | T | C | 0.054 | 0.048 | 0.009 | 0.000 | 27.210 |
| Total fat | 6 | rs9274390 | T | C | 0.145 | -0.035 | 0.007 | 0.000 | 24.771 |
| Total fat | 6 | rs35603463 | C | T | 0.567 | 0.032 | 0.005 | 0.000 | 43.850 |
| Total fat | 6 | rs10948725 | G | C | 0.429 | 0.019 | 0.004 | 0.000 | 20.954 |
| Total fat | 6 | rs12208357 | T | C | 0.070 | 0.054 | 0.008 | 0.000 | 45.724 |
| Total fat | 6 | rs10455872 | G | A | 0.079 | -0.133 | 0.008 | 0.000 | 309.329 |
| Total fat | 6 | rs3869125 | G | A | 0.065 | 0.049 | 0.008 | 0.000 | 35.474 |
| Total fat | 6 | rs117733303 | G | A | 0.019 | -0.241 | 0.015 | 0.000 | 253.978 |
| Total fat | 6 | rs632057 | G | T | 0.628 | -0.028 | 0.004 | 0.000 | 44.267 |
| Total fat | 6 | rs6938550 | A | G | 0.914 | -0.037 | 0.007 | 0.000 | 25.561 |
| Total fat | 7 | rs55747707 | A | G | 0.204 | -0.080 | 0.005 | 0.000 | 247.886 |
| Total fat | 7 | rs4717804 | C | A | 0.930 | -0.042 | 0.008 | 0.000 | 25.119 |
| Total fat | 7 | rs10954732 | A | G | 0.673 | -0.020 | 0.004 | 0.000 | 21.045 |
| Total fat | 7 | rs56001710 | T | A | 0.581 | -0.022 | 0.004 | 0.000 | 25.557 |
| Total fat | 7 | rs2070971 | T | G | 0.137 | 0.028 | 0.006 | 0.000 | 22.929 |
| Total fat | 8 | rs28601761 | G | C | 0.420 | -0.092 | 0.004 | 0.000 | 484.310 |
| Total fat | 8 | rs2326077 | T | C | 0.663 | -0.029 | 0.004 | 0.000 | 46.230 |
| Total fat | 8 | rs8185771 | T | C | 0.956 | 0.046 | 0.010 | 0.000 | 21.260 |
| Total fat | 8 | rs1374300 | C | T | 0.033 | -0.052 | 0.011 | 0.000 | 21.290 |
| Total fat | 8 | rs328 | G | C | 0.100 | -0.096 | 0.007 | 0.000 | 199.503 |
| Total fat | 8 | rs144503444 | C | T | 0.018 | 0.081 | 0.015 | 0.000 | 27.451 |
| Total fat | 8 | rs7831074 | G | C | 0.759 | 0.025 | 0.005 | 0.000 | 23.385 |
| Total fat | 8 | rs2721961 | G | T | 0.281 | -0.028 | 0.005 | 0.000 | 37.735 |
| Total fat | 8 | rs2126259 | C | T | 0.899 | 0.067 | 0.007 | 0.000 | 99.214 |
| Total fat | 9 | rs820503 | A | C | 0.138 | 0.029 | 0.006 | 0.000 | 23.970 |
| Total fat | 9 | rs11789603 | T | C | 0.109 | 0.043 | 0.007 | 0.000 | 42.694 |
| Total fat | 9 | rs4008004 | A | C | 0.222 | 0.029 | 0.005 | 0.000 | 35.736 |
| Total fat | 9 | rs2740488 | C | A | 0.265 | -0.040 | 0.005 | 0.000 | 74.994 |
| Total fat | 9 | rs635634 | T | C | 0.184 | 0.024 | 0.005 | 0.000 | 21.242 |
| Total fat | 10 | rs2478236 | A | G | 0.404 | -0.025 | 0.004 | 0.000 | 34.744 |
| Total fat | 10 | rs1890896 | C | T | 0.527 | -0.020 | 0.004 | 0.000 | 24.849 |
| Total fat | 10 | rs117488242 | G | A | 0.132 | -0.040 | 0.006 | 0.000 | 41.084 |
| Total fat | 10 | rs10884966 | A | G | 0.346 | -0.023 | 0.004 | 0.000 | 28.737 |
| Total fat | 10 | rs112866833 | T | C | 0.290 | 0.022 | 0.005 | 0.000 | 22.704 |
| Total fat | 10 | rs1782652 | A | T | 0.382 | 0.020 | 0.004 | 0.000 | 21.948 |
| Total fat | 10 | rs10761716 | G | C | 0.441 | -0.019 | 0.004 | 0.000 | 21.112 |
| Total fat | 11 | rs102275 | C | T | 0.350 | -0.025 | 0.004 | 0.000 | 35.007 |
| Total fat | 11 | rs12970 | A | G | 0.061 | -0.041 | 0.009 | 0.000 | 22.918 |
| Total fat | 11 | rs141469619 | G | A | 0.010 | 0.193 | 0.022 | 0.000 | 80.359 |
| Total fat | 11 | rs78977265 | A | G | 0.010 | -0.099 | 0.021 | 0.000 | 21.516 |
| Total fat | 11 | rs2970333 | T | C | 0.768 | 0.024 | 0.005 | 0.000 | 25.292 |
| Total fat | 11 | rs1988313 | T | C | 0.690 | -0.027 | 0.005 | 0.000 | 31.420 |
| Total fat | 11 | rs964184 | C | G | 0.867 | -0.178 | 0.006 | 0.000 | 882.573 |
| Total fat | 11 | rs72997616 | A | C | 0.094 | -0.051 | 0.007 | 0.000 | 52.368 |
| Total fat | 12 | rs76895963 | G | T | 0.021 | -0.090 | 0.016 | 0.000 | 32.097 |
| Total fat | 12 | rs67981690 | G | A | 0.129 | 0.028 | 0.006 | 0.000 | 21.108 |
| Total fat | 12 | rs7973253 | G | A | 0.367 | 0.027 | 0.004 | 0.000 | 40.911 |
| Total fat | 12 | rs111283292 | A | G | 0.070 | 0.039 | 0.008 | 0.000 | 23.372 |
| Total fat | 12 | rs7979473 | G | A | 0.613 | -0.029 | 0.004 | 0.000 | 48.410 |
| Total fat | 13 | rs7323466 | C | T | 0.651 | -0.020 | 0.004 | 0.000 | 21.913 |
| Total fat | 13 | rs6602911 | T | C | 0.360 | 0.029 | 0.004 | 0.000 | 47.814 |
| Total fat | 13 | rs149014710 | T | C | 0.431 | -0.022 | 0.005 | 0.000 | 24.828 |
| Total fat | 14 | rs148140473 | T | C | 0.028 | -0.059 | 0.013 | 0.000 | 21.676 |
| Total fat | 14 | rs11620783 | T | C | 0.432 | 0.021 | 0.004 | 0.000 | 26.611 |
| Total fat | 14 | rs12892739 | G | A | 0.360 | -0.020 | 0.004 | 0.000 | 22.252 |
| Total fat | 15 | rs139974673 | C | T | 0.026 | 0.102 | 0.013 | 0.000 | 63.161 |
| Total fat | 15 | rs261290 | C | T | 0.655 | -0.101 | 0.004 | 0.000 | 554.463 |
| Total fat | 15 | rs633695 | G | A | 0.292 | 0.076 | 0.004 | 0.000 | 283.096 |
| Total fat | 15 | rs11247218 | C | T | 0.499 | -0.019 | 0.004 | 0.000 | 21.020 |
| Total fat | 15 | rs11854318 | A | G | 0.275 | -0.038 | 0.005 | 0.000 | 68.907 |
| Total fat | 16 | rs11076175 | G | A | 0.179 | -0.044 | 0.005 | 0.000 | 68.335 |
| Total fat | 16 | rs186956666 | A | C | 0.076 | -0.037 | 0.008 | 0.000 | 21.730 |
| Total fat | 16 | rs2000999 | A | G | 0.189 | 0.031 | 0.005 | 0.000 | 34.467 |
| Total fat | 17 | rs28733475 | G | C | 0.237 | -0.023 | 0.005 | 0.000 | 22.459 |
| Total fat | 17 | rs67919208 | C | T | 0.517 | 0.019 | 0.004 | 0.000 | 22.366 |
| Total fat | 17 | rs62090276 | T | C | 0.147 | -0.026 | 0.006 | 0.000 | 21.050 |
| Total fat | 17 | rs12601919 | G | A | 0.189 | 0.025 | 0.005 | 0.000 | 23.436 |
| Total fat | 17 | rs9910747 | C | A | 0.062 | 0.040 | 0.008 | 0.000 | 22.521 |
| Total fat | 18 | rs77960347 | G | A | 0.013 | 0.218 | 0.018 | 0.000 | 149.470 |
| Total fat | 18 | rs2156552 | T | A | 0.822 | 0.057 | 0.005 | 0.000 | 114.736 |
| Total fat | 19 | rs157592 | C | A | 0.185 | 0.067 | 0.005 | 0.000 | 154.931 |
| Total fat | 19 | rs142385484 | T | C | 0.147 | -0.033 | 0.006 | 0.000 | 32.699 |
| Total fat | 19 | rs56322906 | A | G | 0.035 | -0.093 | 0.011 | 0.000 | 69.915 |
| Total fat | 19 | rs142158911 | A | G | 0.117 | -0.070 | 0.006 | 0.000 | 120.556 |
| Total fat | 19 | rs739320 | C | T | 0.607 | -0.027 | 0.004 | 0.000 | 40.054 |
| Total fat | 19 | rs117188729 | A | T | 0.031 | -0.055 | 0.012 | 0.000 | 21.247 |
| Total fat | 19 | rs58542926 | T | C | 0.074 | -0.140 | 0.008 | 0.000 | 324.507 |
| Total fat | 19 | rs5112 | G | C | 0.534 | 0.075 | 0.004 | 0.000 | 293.412 |
| Total fat | 19 | rs72626214 | T | C | 0.108 | -0.031 | 0.007 | 0.000 | 21.793 |
| Total fat | 20 | rs11907714 | G | A | 0.086 | 0.036 | 0.008 | 0.000 | 22.565 |
| Total fat | 20 | rs1883711 | C | G | 0.031 | 0.088 | 0.012 | 0.000 | 54.044 |
| Total fat | 20 | rs224427 | G | A | 0.211 | -0.025 | 0.005 | 0.000 | 25.403 |
| Total fat | 21 | rs62222988 | C | T | 0.374 | 0.022 | 0.004 | 0.000 | 26.096 |
| Total fat | 22 | rs9616847 | T | A | 0.388 | 0.024 | 0.004 | 0.000 | 32.437 |
| Total fat | 22 | rs5754102 | A | C | 0.183 | -0.027 | 0.005 | 0.000 | 26.268 |
| Carotene | 1 | rs1936052 | T | C | 0.156 | -0.036 | 0.008 | 0.000 | 22.184 |
| Carotene | 1 | rs6660246 | C | A | 0.450 | -0.027 | 0.006 | 0.000 | 23.285 |
| Carotene | 1 | rs12126792 | G | A | 0.012 | -0.135 | 0.028 | 0.000 | 23.138 |
| Carotene | 4 | rs77547747 | C | T | 0.056 | -0.056 | 0.012 | 0.000 | 22.235 |
| Carotene | 5 | rs6596473 | C | G | 0.299 | 0.028 | 0.006 | 0.000 | 21.393 |
| Carotene | 6 | rs62417408 | G | A | 0.038 | -0.069 | 0.015 | 0.000 | 22.241 |
| Carotene | 8 | rs16898247 | A | G | 0.019 | -0.107 | 0.020 | 0.000 | 28.582 |
| Carotene | 9 | rs13295574 | A | G | 0.303 | -0.028 | 0.006 | 0.000 | 21.400 |
| Carotene | 10 | rs3829931 | A | T | 0.973 | 0.083 | 0.018 | 0.000 | 21.810 |
| Carotene | 10 | rs17800766 | C | T | 0.012 | -0.122 | 0.025 | 0.000 | 22.777 |
| Carotene | 10 | rs2998143 | G | A | 0.604 | -0.028 | 0.006 | 0.000 | 22.776 |
| Carotene | 13 | rs4771831 | A | G | 0.352 | -0.027 | 0.006 | 0.000 | 21.184 |
| Carotene | 19 | rs366337 | G | A | 0.937 | 0.054 | 0.011 | 0.000 | 23.481 |
| Carotene | 22 | rs117731008 | A | G | 0.017 | 0.098 | 0.021 | 0.000 | 21.188 |
| Carotene | 22 | rs5760695 | C | T | 0.087 | 0.047 | 0.010 | 0.000 | 21.705 |
| Folate | 2 | rs78074774 | T | C | 0.045 | 0.060 | 0.013 | 0.000 | 20.867 |
| Folate | 2 | rs139588363 | C | T | 0.058 | 0.054 | 0.012 | 0.000 | 21.229 |
| Folate | 3 | rs3772928 | C | T | 0.575 | -0.027 | 0.006 | 0.000 | 24.317 |
| Folate | 7 | rs76630415 | G | T | 0.212 | -0.037 | 0.007 | 0.000 | 31.115 |
| Folate | 8 | rs2449166 | T | C | 0.471 | 0.025 | 0.005 | 0.000 | 21.285 |
| Folate | 10 | rs7074988 | G | A | 0.064 | -0.051 | 0.011 | 0.000 | 21.284 |
| Folate | 16 | rs1502443 | G | C | 0.630 | 0.026 | 0.006 | 0.000 | 21.114 |
| Folate | 17 | rs16956822 | A | G | 0.026 | -0.079 | 0.017 | 0.000 | 21.297 |
| Folate | 18 | rs8085166 | G | A | 0.677 | 0.028 | 0.006 | 0.000 | 22.904 |
| Folate | 18 | rs148031795 | T | C | 0.015 | 0.104 | 0.022 | 0.000 | 21.778 |
| Folate | 20 | rs79975477 | T | C | 0.031 | 0.073 | 0.016 | 0.000 | 21.919 |
| Folate | 22 | rs76802001 | A | G | 0.036 | -0.068 | 0.015 | 0.000 | 21.000 |
| Folate | 22 | rs79748722 | T | C | 0.028 | -0.076 | 0.016 | 0.000 | 21.069 |
| Iron | 1 | rs17257441 | T | C | 0.126 | -0.038 | 0.008 | 0.000 | 20.974 |
| Iron | 2 | rs155599 | C | T | 0.705 | 0.030 | 0.006 | 0.000 | 25.996 |
| Iron | 4 | rs2647238 | C | T | 0.587 | -0.025 | 0.005 | 0.000 | 21.462 |
| Iron | 5 | rs148244439 | T | C | 0.052 | 0.059 | 0.012 | 0.000 | 23.456 |
| Iron | 7 | rs799443 | A | T | 0.669 | -0.030 | 0.006 | 0.000 | 27.512 |
| Iron | 7 | rs56256289 | T | C | 0.187 | 0.033 | 0.007 | 0.000 | 22.292 |
| Iron | 7 | rs6463742 | C | T | 0.484 | 0.026 | 0.006 | 0.000 | 22.124 |
| Iron | 8 | rs9297943 | G | T | 0.642 | 0.027 | 0.006 | 0.000 | 22.213 |
| Iron | 10 | rs1370102 | A | C | 0.431 | -0.025 | 0.005 | 0.000 | 21.338 |
| Iron | 11 | rs118189684 | C | T | 0.055 | 0.059 | 0.012 | 0.000 | 24.152 |
| Iron | 11 | rs114738685 | T | C | 0.056 | 0.055 | 0.012 | 0.000 | 22.270 |
| Iron | 20 | rs116863411 | T | A | 0.018 | -0.095 | 0.020 | 0.000 | 21.497 |
| Magnesium | 1 | rs2745938 | T | G | 0.647 | 0.027 | 0.006 | 0.000 | 22.965 |
| Magnesium | 2 | rs116740989 | A | C | 0.012 | 0.118 | 0.025 | 0.000 | 21.974 |
| Magnesium | 4 | rs114575778 | A | G | 0.010 | -0.145 | 0.027 | 0.000 | 28.000 |
| Magnesium | 5 | rs116028267 | G | C | 0.038 | -0.067 | 0.014 | 0.000 | 21.333 |
| Magnesium | 5 | rs4535437 | G | A | 0.245 | 0.030 | 0.006 | 0.000 | 23.502 |
| Magnesium | 6 | rs114989460 | C | T | 0.018 | 0.096 | 0.021 | 0.000 | 21.002 |
| Magnesium | 7 | rs144862520 | T | C | 0.041 | -0.070 | 0.015 | 0.000 | 20.943 |
| Magnesium | 8 | rs77126457 | A | G | 0.014 | 0.115 | 0.023 | 0.000 | 25.468 |
| Magnesium | 9 | rs76330086 | T | C | 0.015 | -0.102 | 0.022 | 0.000 | 21.084 |
| Magnesium | 9 | rs7022555 | T | C | 0.012 | -0.121 | 0.025 | 0.000 | 22.813 |
| Magnesium | 10 | rs1247081 | T | G | 0.508 | 0.026 | 0.005 | 0.000 | 22.882 |
| Magnesium | 11 | rs116979507 | T | C | 0.039 | 0.064 | 0.014 | 0.000 | 20.985 |
| Magnesium | 11 | rs573905 | G | A | 0.547 | 0.028 | 0.005 | 0.000 | 26.107 |
| Magnesium | 12 | rs147150587 | A | G | 0.010 | 0.128 | 0.028 | 0.000 | 21.561 |
| Magnesium | 13 | rs7339029 | T | G | 0.134 | 0.036 | 0.008 | 0.000 | 20.964 |
| Magnesium | 18 | rs1559583 | C | T | 0.785 | -0.030 | 0.007 | 0.000 | 21.507 |
| Magnesium | 20 | rs111419911 | G | A | 0.310 | -0.029 | 0.006 | 0.000 | 24.805 |
| Vitamin A(Retinol) | 1 | rs12119164 | G | A | 0.742 | 0.030 | 0.006 | 0.000 | 21.550 |
| Vitamin A(Retinol) | 1 | rs692790 | C | T | 0.874 | 0.040 | 0.008 | 0.000 | 23.247 |
| Vitamin A(Retinol) | 2 | rs74977546 | A | G | 0.053 | -0.064 | 0.013 | 0.000 | 25.096 |
| Vitamin A(Retinol) | 3 | rs149577802 | T | C | 0.015 | -0.109 | 0.023 | 0.000 | 21.595 |
| Vitamin A(Retinol) | 10 | rs3213829 | G | T | 0.546 | 0.026 | 0.006 | 0.000 | 21.440 |
| Vitamin A(Retinol) | 14 | rs117669768 | A | G | 0.038 | 0.079 | 0.015 | 0.000 | 28.581 |
| Vitamin A(Retinol) | 16 | rs2126371 | T | C | 0.318 | -0.029 | 0.006 | 0.000 | 23.452 |
| Vitamin A(Retinol) | 16 | rs117219913 | C | T | 0.078 | 0.048 | 0.010 | 0.000 | 21.099 |
| Vitamin A(Retinol) | 20 | rs909570 | A | G | 0.939 | -0.053 | 0.012 | 0.000 | 21.084 |
| Vitamin B1 | 2 | rs35653291 | T | C | 0.053 | -0.277 | 0.060 | 0.000 | 21.028 |
| Vitamin B1 | 2 | rs11678973 | C | G | 0.019 | 0.442 | 0.091 | 0.000 | 23.453 |
| Vitamin B1 | 3 | rs111240651 | A | G | 0.028 | 0.389 | 0.078 | 0.000 | 24.667 |
| Vitamin B1 | 3 | rs13064192 | C | T | 0.243 | 0.144 | 0.030 | 0.000 | 23.122 |
| Vitamin B1 | 4 | rs143171885 | A | C | 0.022 | 0.412 | 0.090 | 0.000 | 21.043 |
| Vitamin B1 | 5 | rs244981 | A | G | 0.017 | -0.512 | 0.106 | 0.000 | 23.142 |
| Vitamin B1 | 8 | rs2047360 | G | A | 0.787 | 0.139 | 0.030 | 0.000 | 20.984 |
| Vitamin B1 | 12 | rs80244203 | C | T | 0.023 | -0.445 | 0.085 | 0.000 | 27.075 |
| Vitamin B1 | 17 | rs72843764 | C | T | 0.019 | -0.475 | 0.093 | 0.000 | 26.365 |
| Vitamin B1 | 19 | rs73527956 | T | C | 0.071 | -0.257 | 0.048 | 0.000 | 28.090 |
| Vitamin B2(Riboflavin) | 6 | rs9455025 | A | G | 0.137 | -0.163 | 0.036 | 0.000 | 20.885 |
| Vitamin B2(Riboflavin) | 6 | rs6930223 | T | G | 0.499 | -0.115 | 0.024 | 0.000 | 22.239 |
| Vitamin B2(Riboflavin) | 6 | rs9345080 | T | A | 0.019 | 0.459 | 0.096 | 0.000 | 23.020 |
| Vitamin B2(Riboflavin) | 8 | rs6993770 | T | A | 0.282 | 0.140 | 0.027 | 0.000 | 25.979 |
| Vitamin B2(Riboflavin) | 13 | rs117540226 | G | C | 0.020 | -0.433 | 0.090 | 0.000 | 23.268 |
| Vitamin B2(Riboflavin) | 15 | rs7182560 | T | C | 0.522 | 0.120 | 0.025 | 0.000 | 22.381 |
| Vitamin B2(Riboflavin) | 17 | rs74725931 | C | T | 0.044 | 0.305 | 0.065 | 0.000 | 21.913 |
| Vitamin B2(Riboflavin) | 19 | rs62143194 | G | C | 0.224 | 0.389 | 0.030 | 0.000 | 168.119 |
| Vitamin B3(Nicotinamide riboside) | 1 | rs115229866 | T | C | 0.021 | 0.196 | 0.041 | 0.000 | 22.761 |
| Vitamin B3(Nicotinamide riboside) | 1 | rs41264825 | T | C | 0.033 | 0.146 | 0.032 | 0.000 | 21.038 |
| Vitamin B3(Nicotinamide riboside) | 2 | rs12476719 | T | A | 0.187 | -0.066 | 0.013 | 0.000 | 24.904 |
| Vitamin B3(Nicotinamide riboside) | 3 | rs79806319 | A | C | 0.010 | -0.233 | 0.051 | 0.000 | 21.011 |
| Vitamin B3(Nicotinamide riboside) | 3 | rs6762494 | C | A | 0.067 | 0.101 | 0.021 | 0.000 | 22.166 |
| Vitamin B3(Nicotinamide riboside) | 5 | rs2560510 | T | C | 0.159 | 0.066 | 0.014 | 0.000 | 20.963 |
| Vitamin B3(Nicotinamide riboside) | 6 | rs181181745 | C | T | 0.021 | -0.202 | 0.041 | 0.000 | 24.177 |
| Vitamin B3(Nicotinamide riboside) | 6 | rs6918986 | C | T | 0.557 | 0.052 | 0.011 | 0.000 | 23.275 |
| Vitamin B3(Nicotinamide riboside) | 10 | rs7908279 | C | T | 0.104 | -0.079 | 0.016 | 0.000 | 23.103 |
| Vitamin B3(Nicotinamide riboside) | 11 | rs17851143 | G | C | 0.123 | -0.073 | 0.015 | 0.000 | 23.466 |
| Vitamin B3(Nicotinamide riboside) | 11 | rs470358 | C | T | 0.571 | 0.051 | 0.011 | 0.000 | 23.799 |
| Vitamin B3(Nicotinamide riboside) | 14 | rs74994396 | T | C | 0.033 | -0.136 | 0.029 | 0.000 | 22.211 |
| Vitamin B3(Nicotinamide riboside) | 15 | rs4775582 | T | G | 0.418 | -0.051 | 0.010 | 0.000 | 24.926 |
| Vitamin B3(Nicotinamide riboside) | 16 | rs1010852 | G | C | 0.803 | -0.060 | 0.013 | 0.000 | 20.804 |
| Vitamin B3(Nicotinamide riboside) | 18 | rs117657891 | A | G | 0.019 | -0.187 | 0.039 | 0.000 | 22.649 |
| Vitamin B6 | 1 | rs188211816 | A | G | 0.029 | -0.079 | 0.016 | 0.000 | 23.321 |
| Vitamin B6 | 2 | rs155599 | C | T | 0.705 | 0.034 | 0.006 | 0.000 | 32.758 |
| Vitamin B6 | 3 | rs3772928 | C | T | 0.575 | -0.029 | 0.006 | 0.000 | 27.925 |
| Vitamin B6 | 4 | rs141933624 | A | G | 0.021 | -0.090 | 0.019 | 0.000 | 21.659 |
| Vitamin B6 | 4 | rs183178622 | T | C | 0.018 | -0.099 | 0.021 | 0.000 | 22.884 |
| Vitamin B6 | 5 | rs77806858 | C | T | 0.070 | -0.051 | 0.011 | 0.000 | 22.663 |
| Vitamin B6 | 6 | rs12198456 | T | C | 0.019 | 0.092 | 0.020 | 0.000 | 21.735 |
| Vitamin B6 | 8 | rs74640671 | T | C | 0.011 | -0.127 | 0.027 | 0.000 | 21.574 |
| Vitamin B6 | 10 | rs12412051 | C | G | 0.034 | 0.071 | 0.015 | 0.000 | 22.387 |
| Vitamin B6 | 11 | rs361294 | C | A | 0.691 | -0.027 | 0.006 | 0.000 | 21.133 |
| Vitamin B6 | 11 | rs12226112 | T | G | 0.341 | 0.028 | 0.006 | 0.000 | 24.492 |
| Vitamin B6 | 13 | rs9560457 | T | C | 0.404 | 0.026 | 0.006 | 0.000 | 21.195 |
| Vitamin B6 | 14 | rs10138490 | C | T | 0.061 | -0.053 | 0.011 | 0.000 | 21.967 |
| Vitamin B6 | 14 | rs34938615 | G | A | 0.011 | -0.122 | 0.026 | 0.000 | 21.296 |
| Vitamin B6 | 16 | rs7205927 | C | A | 0.412 | -0.026 | 0.006 | 0.000 | 21.707 |
| Vitamin B6 | 19 | rs3745438 | C | T | 0.034 | -0.071 | 0.016 | 0.000 | 20.878 |
| Vitamin B6 | 22 | rs67450584 | T | C | 0.158 | 0.037 | 0.007 | 0.000 | 24.225 |
| Vitamin B6 | 22 | rs7292147 | C | G | 0.438 | -0.026 | 0.006 | 0.000 | 22.020 |
| Vitamin B12 | 1 | rs10924919 | T | C | 0.394 | -0.029 | 0.006 | 0.000 | 25.742 |
| Vitamin B12 | 1 | rs112961770 | C | G | 0.023 | -0.089 | 0.018 | 0.000 | 23.234 |
| Vitamin B12 | 5 | rs67568068 | C | T | 0.213 | -0.032 | 0.007 | 0.000 | 22.800 |
| Vitamin B12 | 7 | rs148901823 | G | A | 0.081 | -0.049 | 0.010 | 0.000 | 23.405 |
| Vitamin B12 | 10 | rs12776611 | A | G | 0.022 | -0.088 | 0.019 | 0.000 | 21.596 |
| Vitamin B12 | 10 | rs1419875 | G | T | 0.201 | -0.032 | 0.007 | 0.000 | 21.292 |
| Vitamin B12 | 14 | rs61994378 | C | T | 0.028 | 0.093 | 0.020 | 0.000 | 22.336 |
| Vitamin B12 | 20 | rs6088761 | G | A | 0.390 | 0.029 | 0.006 | 0.000 | 21.790 |
| Vitamin B12 | 22 | rs388561 | C | T | 0.881 | 0.040 | 0.009 | 0.000 | 22.187 |
| Vitamin C | 3 | rs7626478 | A | G | 0.720 | 0.028 | 0.006 | 0.000 | 21.029 |
| Vitamin C | 3 | rs114598078 | T | C | 0.042 | 0.066 | 0.014 | 0.000 | 22.701 |
| Vitamin C | 3 | rs4481190 | C | A | 0.351 | -0.031 | 0.006 | 0.000 | 28.451 |
| Vitamin C | 10 | rs61868302 | T | C | 0.061 | -0.057 | 0.012 | 0.000 | 23.262 |
| Vitamin C | 12 | rs17482258 | T | C | 0.099 | 0.043 | 0.009 | 0.000 | 21.389 |
| Vitamin C | 12 | rs2018201 | G | T | 0.027 | -0.081 | 0.017 | 0.000 | 22.163 |
| Vitamin C | 13 | rs9540734 | A | G | 0.478 | -0.026 | 0.005 | 0.000 | 22.348 |
| Vitamin C | 15 | rs4238567 | C | T | 0.522 | 0.025 | 0.006 | 0.000 | 21.108 |
| Vitamin C | 17 | rs11650824 | A | T | 0.035 | 0.079 | 0.016 | 0.000 | 25.042 |
| Vitamin C | 22 | rs1883993 | A | G | 0.095 | 0.045 | 0.009 | 0.000 | 23.104 |
| Vitamin D | 2 | rs4395237 | G | T | 0.050 | -0.060 | 0.013 | 0.000 | 22.182 |
| Vitamin D | 3 | rs2399949 | C | T | 0.185 | -0.034 | 0.007 | 0.000 | 23.521 |
| Vitamin D | 3 | rs75713989 | T | C | 0.130 | 0.039 | 0.008 | 0.000 | 22.976 |
| Vitamin D | 5 | rs57038272 | T | C | 0.177 | 0.033 | 0.007 | 0.000 | 21.213 |
| Vitamin D | 6 | rs582962 | A | G | 0.684 | -0.028 | 0.006 | 0.000 | 21.687 |
| Vitamin D | 6 | rs9328367 | T | A | 0.510 | 0.026 | 0.006 | 0.000 | 21.725 |
| Vitamin D | 7 | rs117693112 | A | G | 0.041 | 0.068 | 0.014 | 0.000 | 22.215 |
| Vitamin D | 7 | rs679830 | C | T | 0.949 | -0.060 | 0.013 | 0.000 | 22.262 |
| Vitamin D | 7 | rs80261862 | T | C | 0.098 | -0.045 | 0.009 | 0.000 | 23.447 |
| Vitamin D | 9 | rs17301981 | C | T | 0.088 | -0.044 | 0.010 | 0.000 | 20.868 |
| Vitamin D | 10 | rs74593039 | C | G | 0.158 | 0.035 | 0.008 | 0.000 | 21.557 |
| Vitamin D | 12 | rs61942184 | C | G | 0.028 | 0.082 | 0.018 | 0.000 | 21.437 |
| Vitamin D | 14 | rs35775421 | A | G | 0.053 | -0.056 | 0.012 | 0.000 | 20.983 |
| Vitamin D | 18 | rs10469075 | T | C | 0.189 | -0.032 | 0.007 | 0.000 | 21.426 |
| Vitamin E | 1 | rs536912 | A | C | 0.736 | 0.030 | 0.006 | 0.000 | 24.128 |
| Vitamin E | 1 | rs6033 | G | A | 0.072 | -0.052 | 0.011 | 0.000 | 23.945 |
| Vitamin E | 7 | rs2723979 | G | T | 0.584 | -0.027 | 0.006 | 0.000 | 23.183 |
| Vitamin E | 7 | rs979218 | C | A | 0.098 | -0.043 | 0.009 | 0.000 | 21.771 |
| Vitamin E | 9 | rs79966958 | T | C | 0.013 | -0.117 | 0.025 | 0.000 | 22.612 |
| Vitamin E | 11 | rs12421920 | G | A | 0.094 | -0.043 | 0.009 | 0.000 | 21.432 |
| Vitamin E | 13 | rs111306778 | A | G | 0.090 | -0.048 | 0.010 | 0.000 | 25.108 |
| Vitamin E | 14 | rs4903544 | T | C | 0.300 | -0.030 | 0.006 | 0.000 | 24.020 |
| Vitamin E | 15 | rs12899673 | A | G | 0.333 | 0.027 | 0.006 | 0.000 | 21.369 |
| Vitamin E | 17 | rs35218694 | G | A | 0.034 | -0.074 | 0.015 | 0.000 | 23.464 |
| Vitamin E | 17 | rs71385328 | G | A | 0.011 | 0.130 | 0.026 | 0.000 | 24.613 |
| Vitamin E | 22 | rs12165526 | A | T | 0.101 | 0.048 | 0.009 | 0.000 | 27.241 |
| Saturated fatty acid levels | 1 | rs1002687 | A | G | 0.645 | 0.082 | 0.004 | 0.000 | 376.581 |
| Saturated fatty acid levels | 1 | rs660240 | C | T | 0.785 | 0.032 | 0.005 | 0.000 | 40.657 |
| Saturated fatty acid levels | 1 | rs822928 | C | A | 0.530 | 0.020 | 0.004 | 0.000 | 23.610 |
| Saturated fatty acid levels | 1 | rs4846335 | A | C | 0.104 | 0.034 | 0.007 | 0.000 | 26.414 |
| Saturated fatty acid levels | 1 | rs7551124 | T | C | 0.875 | 0.038 | 0.006 | 0.000 | 38.737 |
| Saturated fatty acid levels | 2 | rs11895352 | T | C | 0.475 | -0.027 | 0.004 | 0.000 | 43.298 |
| Saturated fatty acid levels | 2 | rs60960031 | A | G | 0.403 | -0.025 | 0.004 | 0.000 | 37.053 |
| Saturated fatty acid levels | 2 | rs13389219 | T | C | 0.393 | -0.030 | 0.004 | 0.000 | 51.588 |
| Saturated fatty acid levels | 2 | rs10716631 | G | T | 0.536 | -0.021 | 0.004 | 0.000 | 24.030 |
| Saturated fatty acid levels | 2 | rs4564803 | T | G | 0.228 | -0.049 | 0.005 | 0.000 | 104.399 |
| Saturated fatty acid levels | 2 | rs1260326 | C | T | 0.604 | -0.106 | 0.004 | 0.000 | 654.997 |
| Saturated fatty acid levels | 2 | rs2041831 | A | G | 0.454 | 0.020 | 0.004 | 0.000 | 23.074 |
| Saturated fatty acid levels | 3 | rs900048 | T | C | 0.710 | 0.026 | 0.004 | 0.000 | 34.377 |
| Saturated fatty acid levels | 3 | rs143380194 | T | C | 0.016 | -0.078 | 0.016 | 0.000 | 23.403 |
| Saturated fatty acid levels | 4 | rs293435 | T | C | 0.289 | 0.022 | 0.005 | 0.000 | 22.850 |
| Saturated fatty acid levels | 4 | rs6854749 | T | A | 0.198 | 0.032 | 0.005 | 0.000 | 39.759 |
| Saturated fatty acid levels | 4 | rs13108218 | G | A | 0.615 | -0.028 | 0.004 | 0.000 | 43.448 |
| Saturated fatty acid levels | 4 | rs34670304 | A | G | 0.553 | 0.020 | 0.004 | 0.000 | 22.526 |
| Saturated fatty acid levels | 4 | rs2035816 | G | A | 0.084 | -0.039 | 0.007 | 0.000 | 28.052 |
| Saturated fatty acid levels | 4 | rs11940694 | G | A | 0.605 | 0.019 | 0.004 | 0.000 | 21.100 |
| Saturated fatty acid levels | 4 | rs370878539 | G | T | 0.193 | -0.031 | 0.006 | 0.000 | 27.777 |
| Saturated fatty acid levels | 5 | rs3846661 | G | A | 0.398 | 0.026 | 0.004 | 0.000 | 40.494 |
| Saturated fatty acid levels | 5 | rs3936511 | G | A | 0.192 | 0.034 | 0.005 | 0.000 | 42.564 |
| Saturated fatty acid levels | 5 | rs4704834 | G | A | 0.644 | 0.037 | 0.004 | 0.000 | 76.966 |
| Saturated fatty acid levels | 5 | rs10472290 | T | G | 0.199 | -0.025 | 0.005 | 0.000 | 23.093 |
| Saturated fatty acid levels | 5 | rs35764948 | A | T | 0.055 | 0.045 | 0.009 | 0.000 | 25.821 |
| Saturated fatty acid levels | 6 | rs28383314 | C | T | 0.623 | 0.039 | 0.004 | 0.000 | 84.595 |
| Saturated fatty acid levels | 6 | rs3869125 | G | A | 0.065 | 0.042 | 0.008 | 0.000 | 26.212 |
| Saturated fatty acid levels | 6 | rs12055389 | T | C | 0.054 | 0.048 | 0.009 | 0.000 | 27.445 |
| Saturated fatty acid levels | 6 | rs9388530 | T | A | 0.436 | -0.019 | 0.004 | 0.000 | 22.084 |
| Saturated fatty acid levels | 6 | rs6938550 | A | G | 0.914 | -0.037 | 0.007 | 0.000 | 26.629 |
| Saturated fatty acid levels | 6 | rs35603463 | C | T | 0.567 | 0.029 | 0.005 | 0.000 | 34.412 |
| Saturated fatty acid levels | 6 | rs12208357 | T | C | 0.070 | 0.051 | 0.008 | 0.000 | 40.319 |
| Saturated fatty acid levels | 6 | rs117733303 | G | A | 0.019 | -0.238 | 0.015 | 0.000 | 248.192 |
| Saturated fatty acid levels | 6 | rs72972355 | C | T | 0.098 | 0.034 | 0.007 | 0.000 | 23.472 |
| Saturated fatty acid levels | 6 | rs7745751 | A | G | 0.272 | 0.025 | 0.005 | 0.000 | 20.974 |
| Saturated fatty acid levels | 6 | rs540973884 | G | T | 0.599 | -0.028 | 0.004 | 0.000 | 46.011 |
| Saturated fatty acid levels | 6 | rs4475363 | C | T | 0.089 | -0.037 | 0.007 | 0.000 | 26.438 |
| Saturated fatty acid levels | 6 | rs10455872 | G | A | 0.079 | -0.135 | 0.008 | 0.000 | 322.853 |
| Saturated fatty acid levels | 7 | rs2070971 | T | G | 0.137 | 0.030 | 0.006 | 0.000 | 25.965 |
| Saturated fatty acid levels | 7 | rs55747707 | A | G | 0.204 | -0.070 | 0.005 | 0.000 | 189.397 |
| Saturated fatty acid levels | 7 | rs56001710 | T | A | 0.581 | -0.023 | 0.004 | 0.000 | 29.353 |
| Saturated fatty acid levels | 7 | rs41301394 | T | C | 0.281 | 0.022 | 0.005 | 0.000 | 24.333 |
| Saturated fatty acid levels | 8 | rs34120986 | A | G | 0.372 | -0.025 | 0.004 | 0.000 | 33.167 |
| Saturated fatty acid levels | 8 | rs2737245 | T | G | 0.279 | -0.025 | 0.005 | 0.000 | 29.161 |
| Saturated fatty acid levels | 8 | rs139315015 | G | A | 0.105 | -0.084 | 0.007 | 0.000 | 161.346 |
| Saturated fatty acid levels | 8 | rs28550053 | G | A | 0.174 | -0.027 | 0.005 | 0.000 | 24.410 |
| Saturated fatty acid levels | 8 | rs3860847 | A | G | 0.213 | 0.023 | 0.005 | 0.000 | 21.571 |
| Saturated fatty acid levels | 8 | rs10504255 | A | G | 0.663 | -0.028 | 0.004 | 0.000 | 42.932 |
| Saturated fatty acid levels | 8 | rs2126259 | C | T | 0.899 | 0.057 | 0.007 | 0.000 | 71.829 |
| Saturated fatty acid levels | 8 | rs7463479 | A | G | 0.946 | 0.043 | 0.009 | 0.000 | 21.741 |
| Saturated fatty acid levels | 8 | rs28601761 | G | C | 0.420 | -0.091 | 0.004 | 0.000 | 475.515 |
| Saturated fatty acid levels | 9 | rs11789603 | T | C | 0.109 | 0.043 | 0.007 | 0.000 | 43.247 |
| Saturated fatty acid levels | 9 | rs138634755 | A | G | 0.026 | -0.059 | 0.013 | 0.000 | 21.206 |
| Saturated fatty acid levels | 9 | rs2740488 | C | A | 0.265 | -0.036 | 0.005 | 0.000 | 61.941 |
| Saturated fatty acid levels | 9 | rs10810374 | C | A | 0.249 | 0.026 | 0.005 | 0.000 | 30.029 |
| Saturated fatty acid levels | 10 | rs1890896 | C | T | 0.527 | -0.021 | 0.004 | 0.000 | 25.950 |
| Saturated fatty acid levels | 10 | rs10884966 | A | G | 0.346 | -0.023 | 0.004 | 0.000 | 29.189 |
| Saturated fatty acid levels | 10 | rs2478236 | A | G | 0.404 | -0.025 | 0.004 | 0.000 | 36.573 |
| Saturated fatty acid levels | 10 | rs117488242 | G | A | 0.132 | -0.041 | 0.006 | 0.000 | 41.872 |
| Saturated fatty acid levels | 11 | rs79904681 | A | G | 0.011 | -0.089 | 0.019 | 0.000 | 21.563 |
| Saturated fatty acid levels | 11 | rs102275 | C | T | 0.350 | -0.033 | 0.004 | 0.000 | 60.350 |
| Saturated fatty acid levels | 11 | rs964184 | C | G | 0.867 | -0.145 | 0.006 | 0.000 | 588.628 |
| Saturated fatty acid levels | 11 | rs2915407 | T | C | 0.768 | 0.024 | 0.005 | 0.000 | 25.140 |
| Saturated fatty acid levels | 11 | rs7118569 | G | C | 0.090 | -0.046 | 0.007 | 0.000 | 41.332 |
| Saturated fatty acid levels | 11 | rs11039832 | G | A | 0.460 | -0.022 | 0.004 | 0.000 | 28.936 |
| Saturated fatty acid levels | 11 | rs187968762 | A | G | 0.011 | -0.096 | 0.021 | 0.000 | 21.302 |
| Saturated fatty acid levels | 11 | rs141469619 | G | A | 0.010 | 0.164 | 0.021 | 0.000 | 58.255 |
| Saturated fatty acid levels | 12 | rs7979473 | G | A | 0.613 | -0.028 | 0.004 | 0.000 | 44.143 |
| Saturated fatty acid levels | 12 | rs7973253 | G | A | 0.367 | 0.028 | 0.004 | 0.000 | 43.991 |
| Saturated fatty acid levels | 12 | rs111283292 | A | G | 0.070 | 0.040 | 0.008 | 0.000 | 24.704 |
| Saturated fatty acid levels | 12 | rs76895963 | G | T | 0.021 | -0.090 | 0.016 | 0.000 | 32.089 |
| Saturated fatty acid levels | 13 | rs6602911 | T | C | 0.360 | 0.026 | 0.004 | 0.000 | 38.452 |
| Saturated fatty acid levels | 13 | rs149014710 | T | C | 0.431 | -0.022 | 0.005 | 0.000 | 23.833 |
| Saturated fatty acid levels | 14 | rs12892739 | G | A | 0.360 | -0.020 | 0.004 | 0.000 | 22.183 |
| Saturated fatty acid levels | 14 | rs11620783 | T | C | 0.432 | 0.022 | 0.004 | 0.000 | 28.170 |
| Saturated fatty acid levels | 15 | rs7402939 | C | T | 0.624 | 0.021 | 0.004 | 0.000 | 23.781 |
| Saturated fatty acid levels | 15 | rs261290 | C | T | 0.655 | -0.101 | 0.004 | 0.000 | 551.819 |
| Saturated fatty acid levels | 15 | rs11247220 | A | C | 0.499 | -0.019 | 0.004 | 0.000 | 21.346 |
| Saturated fatty acid levels | 15 | rs11854318 | A | G | 0.275 | -0.036 | 0.005 | 0.000 | 62.460 |
| Saturated fatty acid levels | 15 | rs139974673 | C | T | 0.026 | 0.096 | 0.013 | 0.000 | 55.892 |
| Saturated fatty acid levels | 15 | rs633695 | G | A | 0.292 | 0.073 | 0.004 | 0.000 | 264.988 |
| Saturated fatty acid levels | 16 | rs2000999 | A | G | 0.189 | 0.028 | 0.005 | 0.000 | 29.895 |
| Saturated fatty acid levels | 16 | rs2869113 | G | T | 0.360 | 0.022 | 0.005 | 0.000 | 21.677 |
| Saturated fatty acid levels | 16 | rs11076175 | G | A | 0.179 | -0.039 | 0.005 | 0.000 | 53.145 |
| Saturated fatty acid levels | 17 | rs28733475 | G | C | 0.237 | -0.024 | 0.005 | 0.000 | 24.862 |
| Saturated fatty acid levels | 17 | rs4402606 | C | T | 0.716 | 0.025 | 0.005 | 0.000 | 27.485 |
| Saturated fatty acid levels | 17 | rs113366589 | G | T | 0.092 | 0.034 | 0.007 | 0.000 | 22.679 |
| Saturated fatty acid levels | 18 | rs2156552 | T | A | 0.822 | 0.051 | 0.005 | 0.000 | 92.538 |
| Saturated fatty acid levels | 18 | rs77960347 | G | A | 0.013 | 0.197 | 0.018 | 0.000 | 122.325 |
| Saturated fatty acid levels | 19 | rs142158911 | A | G | 0.117 | -0.060 | 0.006 | 0.000 | 88.121 |
| Saturated fatty acid levels | 19 | rs8107974 | T | A | 0.076 | -0.117 | 0.008 | 0.000 | 231.691 |
| Saturated fatty acid levels | 19 | rs142385484 | T | C | 0.147 | -0.030 | 0.006 | 0.000 | 26.011 |
| Saturated fatty acid levels | 19 | rs739320 | C | T | 0.607 | -0.030 | 0.004 | 0.000 | 49.678 |
| Saturated fatty acid levels | 19 | rs157592 | C | A | 0.185 | 0.052 | 0.005 | 0.000 | 94.608 |
| Saturated fatty acid levels | 19 | rs5112 | G | C | 0.534 | 0.062 | 0.004 | 0.000 | 197.746 |
| Saturated fatty acid levels | 20 | rs1883711 | C | G | 0.031 | 0.079 | 0.012 | 0.000 | 44.129 |
| Saturated fatty acid levels | 20 | rs224427 | G | A | 0.211 | -0.023 | 0.005 | 0.000 | 20.987 |
| Saturated fatty acid levels | 20 | rs11907714 | G | A | 0.086 | 0.036 | 0.008 | 0.000 | 22.289 |
| Saturated fatty acid levels | 21 | rs62222988 | C | T | 0.374 | 0.020 | 0.004 | 0.000 | 21.980 |
| Saturated fatty acid levels | 22 | rs5754104 | A | G | 0.189 | -0.026 | 0.005 | 0.000 | 24.422 |
| Saturated fatty acid levels | 22 | rs9616847 | T | A | 0.388 | 0.023 | 0.004 | 0.000 | 30.334 |
| Polyunsaturated fatty acid levels | 1 | rs199717562 | G | A | 0.194 | -0.037 | 0.005 | 0.000 | 46.007 |
| Polyunsaturated fatty acid levels | 1 | rs1002687 | A | G | 0.645 | 0.097 | 0.004 | 0.000 | 533.034 |
| Polyunsaturated fatty acid levels | 1 | rs660240 | C | T | 0.785 | 0.059 | 0.005 | 0.000 | 146.405 |
| Polyunsaturated fatty acid levels | 1 | rs534417 | G | A | 0.875 | 0.039 | 0.006 | 0.000 | 41.444 |
| Polyunsaturated fatty acid levels | 1 | rs2986164 | A | G | 0.536 | -0.022 | 0.004 | 0.000 | 25.024 |
| Polyunsaturated fatty acid levels | 1 | rs7540356 | G | A | 0.420 | -0.019 | 0.004 | 0.000 | 21.226 |
| Polyunsaturated fatty acid levels | 1 | rs553427 | T | C | 0.517 | 0.029 | 0.004 | 0.000 | 54.026 |
| Polyunsaturated fatty acid levels | 2 | rs870526 | T | C | 0.521 | -0.033 | 0.004 | 0.000 | 66.355 |
| Polyunsaturated fatty acid levels | 2 | rs11563251 | T | C | 0.111 | 0.030 | 0.006 | 0.000 | 22.329 |
| Polyunsaturated fatty acid levels | 2 | rs528683975 | T | C | 0.015 | -0.081 | 0.017 | 0.000 | 21.900 |
| Polyunsaturated fatty acid levels | 2 | rs114632083 | T | C | 0.032 | 0.053 | 0.012 | 0.000 | 21.077 |
| Polyunsaturated fatty acid levels | 2 | rs12472790 | A | G | 0.496 | 0.020 | 0.004 | 0.000 | 25.547 |
| Polyunsaturated fatty acid levels | 2 | rs3770586 | T | C | 0.484 | -0.024 | 0.004 | 0.000 | 35.483 |
| Polyunsaturated fatty acid levels | 2 | rs3754692 | T | C | 0.813 | -0.024 | 0.005 | 0.000 | 23.197 |
| Polyunsaturated fatty acid levels | 2 | rs6547409 | T | C | 0.051 | -0.084 | 0.009 | 0.000 | 83.598 |
| Polyunsaturated fatty acid levels | 2 | rs672889 | G | T | 0.860 | 0.074 | 0.006 | 0.000 | 165.264 |
| Polyunsaturated fatty acid levels | 2 | rs1260326 | C | T | 0.604 | -0.077 | 0.004 | 0.000 | 352.778 |
| Polyunsaturated fatty acid levels | 2 | rs1877712 | A | G | 0.561 | -0.020 | 0.004 | 0.000 | 24.673 |
| Polyunsaturated fatty acid levels | 2 | rs6750775 | C | G | 0.082 | -0.039 | 0.007 | 0.000 | 27.039 |
| Polyunsaturated fatty acid levels | 2 | rs4299376 | T | G | 0.676 | -0.032 | 0.004 | 0.000 | 54.429 |
| Polyunsaturated fatty acid levels | 3 | rs10513688 | A | G | 0.098 | 0.031 | 0.007 | 0.000 | 21.797 |
| Polyunsaturated fatty acid levels | 3 | rs6762369 | T | C | 0.648 | 0.020 | 0.004 | 0.000 | 23.575 |
| Polyunsaturated fatty acid levels | 3 | rs34101748 | A | G | 0.046 | -0.048 | 0.010 | 0.000 | 23.135 |
| Polyunsaturated fatty acid levels | 3 | rs7610260 | T | A | 0.678 | -0.020 | 0.004 | 0.000 | 21.507 |
| Polyunsaturated fatty acid levels | 3 | rs73130305 | T | C | 0.219 | -0.023 | 0.005 | 0.000 | 23.138 |
| Polyunsaturated fatty acid levels | 3 | rs6805251 | C | T | 0.615 | -0.020 | 0.004 | 0.000 | 22.950 |
| Polyunsaturated fatty acid levels | 4 | rs335321 | A | G | 0.733 | -0.022 | 0.005 | 0.000 | 23.297 |
| Polyunsaturated fatty acid levels | 4 | rs13108218 | G | A | 0.615 | -0.035 | 0.004 | 0.000 | 69.387 |
| Polyunsaturated fatty acid levels | 4 | rs4860948 | A | T | 0.244 | 0.034 | 0.005 | 0.000 | 52.745 |
| Polyunsaturated fatty acid levels | 4 | rs6854749 | T | A | 0.198 | 0.024 | 0.005 | 0.000 | 22.479 |
| Polyunsaturated fatty acid levels | 5 | rs6882345 | A | G | 0.633 | 0.045 | 0.004 | 0.000 | 115.894 |
| Polyunsaturated fatty acid levels | 5 | rs3843482 | G | T | 0.374 | 0.042 | 0.004 | 0.000 | 101.523 |
| Polyunsaturated fatty acid levels | 6 | rs9295128 | T | G | 0.017 | -0.204 | 0.016 | 0.000 | 165.301 |
| Polyunsaturated fatty acid levels | 6 | rs12212348 | C | T | 0.076 | 0.035 | 0.008 | 0.000 | 21.246 |
| Polyunsaturated fatty acid levels | 6 | rs3822855 | T | G | 0.401 | 0.024 | 0.004 | 0.000 | 35.479 |
| Polyunsaturated fatty acid levels | 6 | rs3179865 | A | G | 0.398 | 0.043 | 0.004 | 0.000 | 95.543 |
| Polyunsaturated fatty acid levels | 6 | rs9270074 | C | T | 0.844 | 0.030 | 0.006 | 0.000 | 29.742 |
| Polyunsaturated fatty acid levels | 6 | rs72848251 | A | G | 0.195 | 0.045 | 0.005 | 0.000 | 73.926 |
| Polyunsaturated fatty acid levels | 6 | rs6938647 | C | A | 0.782 | -0.047 | 0.005 | 0.000 | 90.396 |
| Polyunsaturated fatty acid levels | 6 | rs79834165 | C | T | 0.034 | -0.067 | 0.011 | 0.000 | 36.840 |
| Polyunsaturated fatty acid levels | 6 | rs9343614 | G | A | 0.222 | 0.024 | 0.005 | 0.000 | 25.512 |
| Polyunsaturated fatty acid levels | 6 | rs80254170 | G | A | 0.076 | 0.039 | 0.008 | 0.000 | 26.767 |
| Polyunsaturated fatty acid levels | 7 | rs869412 | C | T | 0.227 | -0.022 | 0.005 | 0.000 | 21.747 |
| Polyunsaturated fatty acid levels | 7 | rs34121855 | G | T | 0.204 | -0.062 | 0.005 | 0.000 | 151.725 |
| Polyunsaturated fatty acid levels | 8 | rs34120986 | A | G | 0.372 | -0.020 | 0.004 | 0.000 | 22.737 |
| Polyunsaturated fatty acid levels | 8 | rs10096633 | T | C | 0.124 | -0.035 | 0.006 | 0.000 | 33.862 |
| Polyunsaturated fatty acid levels | 8 | rs7831074 | G | C | 0.759 | 0.029 | 0.005 | 0.000 | 33.335 |
| Polyunsaturated fatty acid levels | 8 | rs2126259 | C | T | 0.899 | 0.085 | 0.007 | 0.000 | 162.193 |
| Polyunsaturated fatty acid levels | 8 | rs112875651 | A | G | 0.392 | -0.077 | 0.004 | 0.000 | 343.879 |
| Polyunsaturated fatty acid levels | 8 | rs2326077 | T | C | 0.663 | -0.029 | 0.004 | 0.000 | 46.583 |
| Polyunsaturated fatty acid levels | 8 | rs2721961 | G | T | 0.281 | -0.029 | 0.004 | 0.000 | 41.680 |
| Polyunsaturated fatty acid levels | 9 | rs115478735 | T | A | 0.183 | 0.038 | 0.005 | 0.000 | 52.933 |
| Polyunsaturated fatty acid levels | 9 | rs2066714 | C | T | 0.129 | 0.028 | 0.006 | 0.000 | 21.451 |
| Polyunsaturated fatty acid levels | 9 | rs2740488 | C | A | 0.265 | -0.049 | 0.005 | 0.000 | 114.685 |
| Polyunsaturated fatty acid levels | 9 | rs4008004 | A | C | 0.222 | 0.032 | 0.005 | 0.000 | 44.330 |
| Polyunsaturated fatty acid levels | 9 | rs11789603 | T | C | 0.109 | 0.049 | 0.006 | 0.000 | 56.615 |
| Polyunsaturated fatty acid levels | 9 | rs820503 | A | C | 0.138 | 0.028 | 0.006 | 0.000 | 23.252 |
| Polyunsaturated fatty acid levels | 10 | rs4917590 | C | T | 0.078 | -0.039 | 0.007 | 0.000 | 26.963 |
| Polyunsaturated fatty acid levels | 10 | rs1408579 | T | C | 0.495 | 0.018 | 0.004 | 0.000 | 21.346 |
| Polyunsaturated fatty acid levels | 10 | rs11195128 | T | C | 0.346 | -0.021 | 0.004 | 0.000 | 25.646 |
| Polyunsaturated fatty acid levels | 10 | rs112866833 | T | C | 0.290 | 0.028 | 0.004 | 0.000 | 38.272 |
| Polyunsaturated fatty acid levels | 10 | rs117488242 | G | A | 0.132 | -0.040 | 0.006 | 0.000 | 40.538 |
| Polyunsaturated fatty acid levels | 10 | rs11258382 | T | C | 0.262 | -0.023 | 0.005 | 0.000 | 24.754 |
| Polyunsaturated fatty acid levels | 10 | rs11595515 | C | T | 0.297 | -0.020 | 0.004 | 0.000 | 20.897 |
| Polyunsaturated fatty acid levels | 11 | rs964184 | C | G | 0.867 | -0.150 | 0.006 | 0.000 | 648.593 |
| Polyunsaturated fatty acid levels | 11 | rs102275 | C | T | 0.350 | -0.097 | 0.004 | 0.000 | 540.797 |
| Polyunsaturated fatty acid levels | 11 | rs72997616 | A | C | 0.094 | -0.066 | 0.007 | 0.000 | 91.960 |
| Polyunsaturated fatty acid levels | 11 | rs141469619 | G | A | 0.010 | 0.129 | 0.021 | 0.000 | 37.313 |
| Polyunsaturated fatty acid levels | 11 | rs368424410 | T | C | 0.270 | 0.032 | 0.005 | 0.000 | 38.358 |
| Polyunsaturated fatty acid levels | 11 | rs2229738 | T | C | 0.066 | -0.040 | 0.008 | 0.000 | 25.168 |
| Polyunsaturated fatty acid levels | 11 | rs12970 | A | G | 0.061 | -0.044 | 0.008 | 0.000 | 26.887 |
| Polyunsaturated fatty acid levels | 12 | rs76895963 | G | T | 0.021 | -0.073 | 0.016 | 0.000 | 21.722 |
| Polyunsaturated fatty acid levels | 12 | rs12369145 | G | A | 0.090 | -0.034 | 0.007 | 0.000 | 23.352 |
| Polyunsaturated fatty acid levels | 12 | rs7970695 | A | G | 0.621 | -0.031 | 0.004 | 0.000 | 57.893 |
| Polyunsaturated fatty acid levels | 12 | rs11057602 | C | T | 0.611 | -0.020 | 0.004 | 0.000 | 23.276 |
| Polyunsaturated fatty acid levels | 12 | rs4766578 | A | T | 0.503 | 0.027 | 0.004 | 0.000 | 45.235 |
| Polyunsaturated fatty acid levels | 13 | rs41291219 | A | G | 0.016 | -0.079 | 0.017 | 0.000 | 21.727 |
| Polyunsaturated fatty acid levels | 13 | rs6602911 | T | C | 0.360 | 0.028 | 0.004 | 0.000 | 45.801 |
| Polyunsaturated fatty acid levels | 13 | rs7337784 | C | G | 0.214 | -0.024 | 0.005 | 0.000 | 23.386 |
| Polyunsaturated fatty acid levels | 14 | rs7144265 | T | C | 0.121 | 0.029 | 0.006 | 0.000 | 21.571 |
| Polyunsaturated fatty acid levels | 14 | rs72694391 | C | T | 0.482 | 0.019 | 0.004 | 0.000 | 22.491 |
| Polyunsaturated fatty acid levels | 14 | rs148140473 | T | C | 0.028 | -0.063 | 0.013 | 0.000 | 25.537 |
| Polyunsaturated fatty acid levels | 15 | rs261290 | C | T | 0.655 | -0.112 | 0.004 | 0.000 | 701.731 |
| Polyunsaturated fatty acid levels | 15 | rs633695 | G | A | 0.292 | 0.085 | 0.004 | 0.000 | 365.151 |
| Polyunsaturated fatty acid levels | 15 | rs11854242 | T | C | 0.275 | -0.043 | 0.005 | 0.000 | 92.124 |
| Polyunsaturated fatty acid levels | 15 | rs139974673 | C | T | 0.026 | 0.066 | 0.013 | 0.000 | 27.003 |
| Polyunsaturated fatty acid levels | 16 | rs76116020 | G | A | 0.044 | -0.059 | 0.010 | 0.000 | 36.283 |
| Polyunsaturated fatty acid levels | 16 | rs1345849 | G | A | 0.859 | -0.027 | 0.006 | 0.000 | 22.668 |
| Polyunsaturated fatty acid levels | 16 | rs34955778 | C | T | 0.420 | -0.030 | 0.004 | 0.000 | 53.810 |
| Polyunsaturated fatty acid levels | 16 | rs3764261 | A | C | 0.324 | 0.056 | 0.004 | 0.000 | 170.793 |
| Polyunsaturated fatty acid levels | 17 | rs56325564 | A | G | 0.483 | 0.024 | 0.004 | 0.000 | 36.587 |
| Polyunsaturated fatty acid levels | 17 | rs11650776 | A | G | 0.750 | 0.021 | 0.005 | 0.000 | 21.133 |
| Polyunsaturated fatty acid levels | 17 | rs1050541 | G | T | 0.555 | -0.021 | 0.004 | 0.000 | 24.527 |
| Polyunsaturated fatty acid levels | 17 | rs12948283 | C | G | 0.297 | 0.022 | 0.005 | 0.000 | 23.209 |
| Polyunsaturated fatty acid levels | 17 | rs740516 | G | C | 0.151 | -0.029 | 0.006 | 0.000 | 26.246 |
| Polyunsaturated fatty acid levels | 18 | rs9304381 | T | C | 0.818 | 0.073 | 0.005 | 0.000 | 198.722 |
| Polyunsaturated fatty acid levels | 18 | rs78277979 | A | C | 0.142 | 0.030 | 0.006 | 0.000 | 26.959 |
| Polyunsaturated fatty acid levels | 18 | rs77960347 | G | A | 0.013 | 0.273 | 0.018 | 0.000 | 243.345 |
| Polyunsaturated fatty acid levels | 19 | rs56322906 | A | G | 0.035 | -0.104 | 0.011 | 0.000 | 91.888 |
| Polyunsaturated fatty acid levels | 19 | rs58542926 | T | C | 0.074 | -0.153 | 0.008 | 0.000 | 400.876 |
| Polyunsaturated fatty acid levels | 19 | rs74747585 | C | T | 0.025 | -0.070 | 0.013 | 0.000 | 28.253 |
| Polyunsaturated fatty acid levels | 19 | rs1081105 | C | A | 0.028 | 0.119 | 0.012 | 0.000 | 94.177 |
| Polyunsaturated fatty acid levels | 19 | rs1065853 | T | G | 0.081 | -0.169 | 0.007 | 0.000 | 523.615 |
| Polyunsaturated fatty acid levels | 19 | rs79429216 | A | G | 0.013 | 0.162 | 0.018 | 0.000 | 82.364 |
| Polyunsaturated fatty acid levels | 19 | rs73013176 | C | T | 0.011 | -0.103 | 0.019 | 0.000 | 28.290 |
| Polyunsaturated fatty acid levels | 19 | rs142158911 | A | G | 0.117 | -0.092 | 0.006 | 0.000 | 213.922 |
| Polyunsaturated fatty acid levels | 19 | rs10419198 | T | C | 0.253 | 0.025 | 0.005 | 0.000 | 30.587 |
| Polyunsaturated fatty acid levels | 19 | rs17569 | A | G | 0.157 | 0.025 | 0.005 | 0.000 | 21.416 |
| Polyunsaturated fatty acid levels | 19 | rs56233644 | A | C | 0.366 | -0.020 | 0.004 | 0.000 | 23.443 |
| Polyunsaturated fatty acid levels | 19 | rs11667604 | A | T | 0.399 | -0.019 | 0.004 | 0.000 | 21.234 |
| Polyunsaturated fatty acid levels | 20 | rs1883711 | C | G | 0.031 | 0.092 | 0.012 | 0.000 | 61.569 |
| Polyunsaturated fatty acid levels | 20 | rs1800961 | T | C | 0.030 | -0.073 | 0.012 | 0.000 | 38.342 |
| Polyunsaturated fatty acid levels | 20 | rs2378390 | A | G | 0.141 | -0.032 | 0.006 | 0.000 | 31.170 |
| Polyunsaturated fatty acid levels | 22 | rs5754102 | A | C | 0.183 | -0.030 | 0.005 | 0.000 | 32.564 |
| Polyunsaturated fatty acid levels | 22 | rs9616847 | T | A | 0.388 | 0.023 | 0.004 | 0.000 | 30.262 |
| Monounsaturated fatty acid levels | 1 | rs71640850 | A | T | 0.160 | 0.026 | 0.006 | 0.000 | 21.952 |
| Monounsaturated fatty acid levels | 1 | rs641154 | T | C | 0.493 | 0.021 | 0.004 | 0.000 | 25.494 |
| Monounsaturated fatty acid levels | 1 | rs4846335 | A | C | 0.104 | 0.031 | 0.007 | 0.000 | 21.637 |
| Monounsaturated fatty acid levels | 1 | rs182050989 | T | C | 0.028 | 0.069 | 0.012 | 0.000 | 31.848 |
| Monounsaturated fatty acid levels | 1 | rs857152 | T | C | 0.115 | 0.030 | 0.006 | 0.000 | 21.377 |
| Monounsaturated fatty acid levels | 1 | rs1002687 | A | G | 0.645 | 0.091 | 0.004 | 0.000 | 463.411 |
| Monounsaturated fatty acid levels | 1 | rs602633 | G | T | 0.783 | 0.027 | 0.005 | 0.000 | 29.910 |
| Monounsaturated fatty acid levels | 1 | rs4846914 | A | G | 0.605 | -0.031 | 0.004 | 0.000 | 53.786 |
| Monounsaturated fatty acid levels | 1 | rs534417 | G | A | 0.875 | 0.039 | 0.006 | 0.000 | 40.114 |
| Monounsaturated fatty acid levels | 2 | rs78877131 | C | G | 0.048 | 0.044 | 0.010 | 0.000 | 21.176 |
| Monounsaturated fatty acid levels | 2 | rs1260326 | C | T | 0.604 | -0.111 | 0.004 | 0.000 | 713.942 |
| Monounsaturated fatty acid levels | 2 | rs2943635 | T | C | 0.680 | 0.026 | 0.004 | 0.000 | 36.491 |
| Monounsaturated fatty acid levels | 2 | rs4564803 | T | G | 0.228 | -0.072 | 0.005 | 0.000 | 219.696 |
| Monounsaturated fatty acid levels | 2 | rs35627330 | A | G | 0.013 | 0.088 | 0.019 | 0.000 | 21.246 |
| Monounsaturated fatty acid levels | 2 | rs78096412 | G | T | 0.110 | -0.032 | 0.007 | 0.000 | 23.114 |
| Monounsaturated fatty acid levels | 2 | rs60960031 | A | G | 0.403 | -0.026 | 0.004 | 0.000 | 39.735 |
| Monounsaturated fatty acid levels | 2 | rs62183700 | C | G | 0.329 | 0.020 | 0.004 | 0.000 | 21.089 |
| Monounsaturated fatty acid levels | 2 | rs35757519 | C | T | 0.447 | 0.019 | 0.004 | 0.000 | 21.191 |
| Monounsaturated fatty acid levels | 2 | rs11895352 | T | C | 0.475 | -0.026 | 0.004 | 0.000 | 40.613 |
| Monounsaturated fatty acid levels | 2 | rs1128249 | T | G | 0.392 | -0.036 | 0.004 | 0.000 | 73.024 |
| Monounsaturated fatty acid levels | 3 | rs79287178 | A | G | 0.031 | 0.057 | 0.012 | 0.000 | 21.353 |
| Monounsaturated fatty acid levels | 3 | rs1394092 | T | C | 0.727 | 0.031 | 0.005 | 0.000 | 45.564 |
| Monounsaturated fatty acid levels | 3 | rs4683708 | T | C | 0.533 | -0.019 | 0.004 | 0.000 | 22.591 |
| Monounsaturated fatty acid levels | 3 | rs12637730 | T | C | 0.145 | 0.027 | 0.006 | 0.000 | 21.794 |
| Monounsaturated fatty acid levels | 3 | rs9968117 | T | C | 0.121 | -0.031 | 0.006 | 0.000 | 24.636 |
| Monounsaturated fatty acid levels | 4 | rs5020545 | T | C | 0.447 | 0.020 | 0.004 | 0.000 | 23.935 |
| Monounsaturated fatty acid levels | 4 | rs2035816 | G | A | 0.084 | -0.041 | 0.007 | 0.000 | 31.613 |
| Monounsaturated fatty acid levels | 4 | rs4541525 | C | T | 0.306 | -0.023 | 0.005 | 0.000 | 22.794 |
| Monounsaturated fatty acid levels | 4 | rs11940694 | G | A | 0.605 | 0.025 | 0.004 | 0.000 | 36.386 |
| Monounsaturated fatty acid levels | 4 | rs16895971 | C | T | 0.137 | 0.029 | 0.006 | 0.000 | 23.992 |
| Monounsaturated fatty acid levels | 4 | rs13108218 | G | A | 0.615 | -0.038 | 0.004 | 0.000 | 81.055 |
| Monounsaturated fatty acid levels | 4 | rs1471251 | T | A | 0.397 | 0.030 | 0.004 | 0.000 | 51.972 |
| Monounsaturated fatty acid levels | 4 | rs4491981 | A | C | 0.241 | -0.024 | 0.005 | 0.000 | 26.204 |
| Monounsaturated fatty acid levels | 5 | rs10472290 | T | G | 0.199 | -0.027 | 0.005 | 0.000 | 26.949 |
| Monounsaturated fatty acid levels | 5 | rs35764948 | A | T | 0.055 | 0.045 | 0.009 | 0.000 | 24.863 |
| Monounsaturated fatty acid levels | 5 | rs3936511 | G | A | 0.192 | 0.043 | 0.005 | 0.000 | 68.743 |
| Monounsaturated fatty acid levels | 5 | rs12916 | C | T | 0.400 | 0.023 | 0.004 | 0.000 | 30.381 |
| Monounsaturated fatty acid levels | 5 | rs4704834 | G | A | 0.644 | 0.044 | 0.004 | 0.000 | 105.120 |
| Monounsaturated fatty acid levels | 5 | rs4976647 | C | A | 0.334 | 0.020 | 0.004 | 0.000 | 21.456 |
| Monounsaturated fatty acid levels | 5 | rs72784652 | T | G | 0.061 | -0.041 | 0.009 | 0.000 | 22.775 |
| Monounsaturated fatty acid levels | 5 | rs13188623 | C | T | 0.183 | -0.026 | 0.005 | 0.000 | 24.356 |
| Monounsaturated fatty acid levels | 5 | rs4704555 | C | T | 0.569 | 0.021 | 0.004 | 0.000 | 25.961 |
| Monounsaturated fatty acid levels | 6 | rs632057 | G | T | 0.628 | -0.031 | 0.004 | 0.000 | 55.735 |
| Monounsaturated fatty acid levels | 6 | rs539981616 | G | C | 0.292 | -0.028 | 0.005 | 0.000 | 33.128 |
| Monounsaturated fatty acid levels | 6 | rs6938550 | A | G | 0.914 | -0.042 | 0.007 | 0.000 | 32.917 |
| Monounsaturated fatty acid levels | 6 | rs2800709 | G | T | 0.520 | 0.020 | 0.004 | 0.000 | 23.082 |
| Monounsaturated fatty acid levels | 6 | rs10455872 | G | A | 0.079 | -0.148 | 0.008 | 0.000 | 387.818 |
| Monounsaturated fatty acid levels | 6 | rs12208357 | T | C | 0.070 | 0.053 | 0.008 | 0.000 | 43.893 |
| Monounsaturated fatty acid levels | 6 | rs1052248 | A | T | 0.258 | 0.031 | 0.005 | 0.000 | 45.143 |
| Monounsaturated fatty acid levels | 6 | rs6905288 | A | G | 0.569 | 0.023 | 0.004 | 0.000 | 30.371 |
| Monounsaturated fatty acid levels | 6 | rs73022312 | G | A | 0.011 | 0.096 | 0.020 | 0.000 | 23.154 |
| Monounsaturated fatty acid levels | 6 | rs67201184 | G | T | 0.491 | 0.019 | 0.004 | 0.000 | 21.146 |
| Monounsaturated fatty acid levels | 6 | rs184089815 | A | G | 0.016 | -0.077 | 0.016 | 0.000 | 22.775 |
| Monounsaturated fatty acid levels | 6 | rs28752924 | G | T | 0.244 | 0.029 | 0.006 | 0.000 | 26.195 |
| Monounsaturated fatty acid levels | 6 | rs9271573 | C | A | 0.594 | 0.043 | 0.004 | 0.000 | 107.410 |
| Monounsaturated fatty acid levels | 6 | rs138345261 | A | G | 0.024 | 0.063 | 0.014 | 0.000 | 21.171 |
| Monounsaturated fatty acid levels | 6 | rs12212146 | C | T | 0.074 | 0.049 | 0.008 | 0.000 | 37.297 |
| Monounsaturated fatty acid levels | 6 | rs117733303 | G | A | 0.019 | -0.258 | 0.015 | 0.000 | 292.360 |
| Monounsaturated fatty acid levels | 7 | rs56001710 | T | A | 0.581 | -0.027 | 0.004 | 0.000 | 39.949 |
| Monounsaturated fatty acid levels | 7 | rs10954732 | A | G | 0.673 | -0.020 | 0.004 | 0.000 | 21.399 |
| Monounsaturated fatty acid levels | 7 | rs3812316 | G | C | 0.129 | -0.113 | 0.006 | 0.000 | 350.469 |
| Monounsaturated fatty acid levels | 7 | rs2070971 | T | G | 0.137 | 0.032 | 0.006 | 0.000 | 29.844 |
| Monounsaturated fatty acid levels | 7 | rs72555385 | G | A | 0.049 | 0.061 | 0.009 | 0.000 | 41.588 |
| Monounsaturated fatty acid levels | 8 | rs150564454 | A | G | 0.011 | -0.092 | 0.020 | 0.000 | 21.347 |
| Monounsaturated fatty acid levels | 8 | rs10102524 | G | A | 0.549 | -0.034 | 0.004 | 0.000 | 67.811 |
| Monounsaturated fatty acid levels | 8 | rs1018070 | A | T | 0.054 | 0.046 | 0.009 | 0.000 | 25.564 |
| Monounsaturated fatty acid levels | 8 | rs3860847 | A | G | 0.213 | 0.025 | 0.005 | 0.000 | 25.512 |
| Monounsaturated fatty acid levels | 8 | rs11776943 | T | C | 0.075 | 0.037 | 0.008 | 0.000 | 23.234 |
| Monounsaturated fatty acid levels | 8 | rs2889 | G | A | 0.315 | -0.020 | 0.004 | 0.000 | 21.360 |
| Monounsaturated fatty acid levels | 8 | rs1993453 | G | A | 0.663 | -0.026 | 0.004 | 0.000 | 36.244 |
| Monounsaturated fatty acid levels | 8 | rs8175519 | A | G | 0.154 | -0.034 | 0.007 | 0.000 | 22.532 |
| Monounsaturated fatty acid levels | 8 | rs1464093 | G | A | 0.193 | 0.024 | 0.005 | 0.000 | 21.661 |
| Monounsaturated fatty acid levels | 8 | rs28601761 | G | C | 0.420 | -0.089 | 0.004 | 0.000 | 459.575 |
| Monounsaturated fatty acid levels | 8 | rs2721961 | G | T | 0.281 | -0.026 | 0.005 | 0.000 | 32.655 |
| Monounsaturated fatty acid levels | 8 | rs59347135 | G | C | 0.046 | 0.055 | 0.010 | 0.000 | 30.183 |
| Monounsaturated fatty acid levels | 8 | rs328 | G | C | 0.100 | -0.145 | 0.007 | 0.000 | 458.710 |
| Monounsaturated fatty acid levels | 9 | rs820503 | A | C | 0.138 | 0.027 | 0.006 | 0.000 | 21.001 |
| Monounsaturated fatty acid levels | 9 | rs2849049 | A | C | 0.327 | 0.022 | 0.004 | 0.000 | 24.504 |
| Monounsaturated fatty acid levels | 9 | rs7038652 | A | G | 0.065 | -0.041 | 0.009 | 0.000 | 22.972 |
| Monounsaturated fatty acid levels | 9 | rs2740488 | C | A | 0.265 | -0.028 | 0.005 | 0.000 | 36.633 |
| Monounsaturated fatty acid levels | 10 | rs10884966 | A | G | 0.346 | -0.020 | 0.004 | 0.000 | 21.156 |
| Monounsaturated fatty acid levels | 10 | rs117488242 | G | A | 0.132 | -0.031 | 0.006 | 0.000 | 24.616 |
| Monounsaturated fatty acid levels | 10 | rs2497337 | T | C | 0.513 | 0.022 | 0.004 | 0.000 | 29.544 |
| Monounsaturated fatty acid levels | 10 | rs7894497 | A | T | 0.480 | 0.019 | 0.004 | 0.000 | 22.268 |
| Monounsaturated fatty acid levels | 10 | rs526748 | T | G | 0.554 | 0.025 | 0.004 | 0.000 | 38.113 |
| Monounsaturated fatty acid levels | 10 | rs2311738 | C | T | 0.291 | 0.021 | 0.004 | 0.000 | 22.512 |
| Monounsaturated fatty acid levels | 10 | rs10761716 | G | C | 0.441 | -0.031 | 0.004 | 0.000 | 56.646 |
| Monounsaturated fatty acid levels | 11 | rs6486121 | T | C | 0.639 | 0.019 | 0.004 | 0.000 | 20.980 |
| Monounsaturated fatty acid levels | 11 | rs11231694 | C | G | 0.056 | 0.042 | 0.009 | 0.000 | 21.615 |
| Monounsaturated fatty acid levels | 11 | rs141469619 | G | A | 0.010 | 0.246 | 0.021 | 0.000 | 130.680 |
| Monounsaturated fatty acid levels | 11 | rs964184 | C | G | 0.867 | -0.205 | 0.006 | 0.000 | 1172.223 |
| Monounsaturated fatty acid levels | 11 | rs72997616 | A | C | 0.094 | -0.032 | 0.007 | 0.000 | 21.515 |
| Monounsaturated fatty acid levels | 11 | rs11039832 | G | A | 0.460 | -0.020 | 0.004 | 0.000 | 22.532 |
| Monounsaturated fatty acid levels | 11 | rs174564 | G | A | 0.347 | 0.064 | 0.004 | 0.000 | 225.890 |
| Monounsaturated fatty acid levels | 11 | rs2513048 | C | T | 0.712 | -0.025 | 0.005 | 0.000 | 29.089 |
| Monounsaturated fatty acid levels | 11 | rs2915400 | C | T | 0.768 | 0.024 | 0.005 | 0.000 | 24.684 |
| Monounsaturated fatty acid levels | 11 | rs883863 | A | G | 0.308 | 0.022 | 0.004 | 0.000 | 24.275 |
| Monounsaturated fatty acid levels | 12 | rs7973253 | G | A | 0.367 | 0.026 | 0.004 | 0.000 | 38.395 |
| Monounsaturated fatty acid levels | 12 | rs76895963 | G | T | 0.021 | -0.091 | 0.016 | 0.000 | 32.764 |
| Monounsaturated fatty acid levels | 12 | rs67981690 | G | A | 0.129 | 0.035 | 0.006 | 0.000 | 33.446 |
| Monounsaturated fatty acid levels | 12 | rs7979473 | G | A | 0.613 | -0.023 | 0.004 | 0.000 | 29.678 |
| Monounsaturated fatty acid levels | 12 | rs11172134 | A | T | 0.201 | -0.027 | 0.005 | 0.000 | 29.020 |
| Monounsaturated fatty acid levels | 12 | rs111283292 | A | G | 0.070 | 0.037 | 0.008 | 0.000 | 21.269 |
| Monounsaturated fatty acid levels | 13 | rs149014710 | T | C | 0.431 | -0.025 | 0.005 | 0.000 | 30.303 |
| Monounsaturated fatty acid levels | 13 | rs7140110 | C | T | 0.299 | 0.031 | 0.004 | 0.000 | 48.185 |
| Monounsaturated fatty acid levels | 13 | rs7323466 | C | T | 0.651 | -0.022 | 0.004 | 0.000 | 25.745 |
| Monounsaturated fatty acid levels | 14 | rs12892739 | G | A | 0.360 | -0.020 | 0.004 | 0.000 | 21.829 |
| Monounsaturated fatty acid levels | 15 | rs261290 | C | T | 0.655 | -0.073 | 0.004 | 0.000 | 291.903 |
| Monounsaturated fatty acid levels | 15 | rs11854318 | A | G | 0.275 | -0.028 | 0.005 | 0.000 | 38.067 |
| Monounsaturated fatty acid levels | 15 | rs150844304 | C | A | 0.026 | 0.123 | 0.013 | 0.000 | 91.994 |
| Monounsaturated fatty acid levels | 15 | rs633695 | G | A | 0.292 | 0.056 | 0.004 | 0.000 | 156.329 |
| Monounsaturated fatty acid levels | 15 | rs7402939 | C | T | 0.624 | 0.023 | 0.004 | 0.000 | 28.275 |
| Monounsaturated fatty acid levels | 15 | rs2680888 | A | C | 0.872 | 0.032 | 0.006 | 0.000 | 26.790 |
| Monounsaturated fatty acid levels | 16 | rs3198697 | T | C | 0.407 | -0.027 | 0.004 | 0.000 | 41.309 |
| Monounsaturated fatty acid levels | 16 | rs12918075 | A | G | 0.015 | 0.086 | 0.019 | 0.000 | 20.902 |
| Monounsaturated fatty acid levels | 16 | rs2000999 | A | G | 0.189 | 0.030 | 0.005 | 0.000 | 33.005 |
| Monounsaturated fatty acid levels | 17 | rs62090276 | T | C | 0.147 | -0.027 | 0.006 | 0.000 | 22.267 |
| Monounsaturated fatty acid levels | 17 | rs117851947 | T | A | 0.014 | 0.088 | 0.019 | 0.000 | 20.928 |
| Monounsaturated fatty acid levels | 17 | rs112259268 | A | C | 0.029 | 0.068 | 0.012 | 0.000 | 30.818 |
| Monounsaturated fatty acid levels | 17 | rs9910747 | C | A | 0.062 | 0.047 | 0.008 | 0.000 | 30.527 |
| Monounsaturated fatty acid levels | 17 | rs12601919 | G | A | 0.189 | 0.030 | 0.005 | 0.000 | 32.001 |
| Monounsaturated fatty acid levels | 18 | rs149615216 | T | C | 0.011 | 0.166 | 0.020 | 0.000 | 70.560 |
| Monounsaturated fatty acid levels | 18 | rs1540037 | G | A | 0.778 | 0.033 | 0.005 | 0.000 | 44.533 |
| Monounsaturated fatty acid levels | 19 | rs182611493 | G | A | 0.013 | -0.147 | 0.020 | 0.000 | 56.613 |
| Monounsaturated fatty acid levels | 19 | rs142385484 | T | C | 0.147 | -0.035 | 0.006 | 0.000 | 37.284 |
| Monounsaturated fatty acid levels | 19 | rs116843064 | A | G | 0.020 | -0.162 | 0.015 | 0.000 | 123.298 |
| Monounsaturated fatty acid levels | 19 | rs157592 | C | A | 0.185 | 0.053 | 0.005 | 0.000 | 98.871 |
| Monounsaturated fatty acid levels | 19 | rs117188729 | A | T | 0.031 | -0.067 | 0.012 | 0.000 | 32.388 |
| Monounsaturated fatty acid levels | 19 | rs5112 | G | C | 0.534 | 0.081 | 0.004 | 0.000 | 340.264 |
| Monounsaturated fatty acid levels | 19 | rs8107974 | T | A | 0.076 | -0.118 | 0.008 | 0.000 | 234.129 |
| Monounsaturated fatty acid levels | 19 | rs739320 | C | T | 0.607 | -0.028 | 0.004 | 0.000 | 42.763 |
| Monounsaturated fatty acid levels | 20 | rs7679 | C | T | 0.186 | 0.037 | 0.005 | 0.000 | 49.195 |
| Monounsaturated fatty acid levels | 20 | rs11907714 | G | A | 0.086 | 0.036 | 0.008 | 0.000 | 22.144 |
| Monounsaturated fatty acid levels | 20 | rs2902941 | G | A | 0.355 | -0.020 | 0.004 | 0.000 | 23.035 |
| Monounsaturated fatty acid levels | 21 | rs62222988 | C | T | 0.374 | 0.022 | 0.004 | 0.000 | 28.363 |
| Monounsaturated fatty acid levels | 22 | rs9616847 | T | A | 0.388 | 0.022 | 0.004 | 0.000 | 26.322 |
| Englyst dietary fibre | 1 | rs28447224 | A | T | 0.238 | -0.032 | 0.006 | 0.000 | 24.702 |
| Englyst dietary fibre | 1 | rs7542974 | A | G | 0.253 | 0.032 | 0.006 | 0.000 | 26.012 |
| Englyst dietary fibre | 1 | rs17119113 | G | A | 0.083 | 0.050 | 0.010 | 0.000 | 25.321 |
| Englyst dietary fibre | 2 | rs1595396 | T | G | 0.366 | 0.026 | 0.006 | 0.000 | 21.406 |
| Englyst dietary fibre | 2 | rs4668107 | A | T | 0.576 | 0.025 | 0.006 | 0.000 | 21.136 |
| Englyst dietary fibre | 2 | rs6752846 | T | C | 0.581 | 0.027 | 0.006 | 0.000 | 23.686 |
| Englyst dietary fibre | 3 | rs13078307 | A | C | 0.241 | 0.032 | 0.006 | 0.000 | 24.149 |
| Englyst dietary fibre | 4 | rs149073968 | C | G | 0.032 | 0.076 | 0.016 | 0.000 | 22.540 |
| Englyst dietary fibre | 6 | rs80026531 | G | A | 0.033 | -0.075 | 0.015 | 0.000 | 23.751 |
| Englyst dietary fibre | 7 | rs2269903 | C | A | 0.128 | 0.038 | 0.008 | 0.000 | 21.689 |
| Englyst dietary fibre | 7 | rs10952057 | C | T | 0.887 | -0.040 | 0.009 | 0.000 | 21.632 |
| Englyst dietary fibre | 8 | rs2543067 | C | T | 0.721 | 0.028 | 0.006 | 0.000 | 20.944 |
| Englyst dietary fibre | 9 | rs7023856 | A | G | 0.265 | -0.028 | 0.006 | 0.000 | 21.021 |
| Englyst dietary fibre | 10 | rs79219143 | A | G | 0.046 | 0.067 | 0.014 | 0.000 | 21.935 |
| Englyst dietary fibre | 11 | rs633683 | C | T | 0.599 | 0.026 | 0.006 | 0.000 | 22.270 |
| Englyst dietary fibre | 11 | rs12226112 | T | G | 0.341 | 0.026 | 0.006 | 0.000 | 20.987 |
| Englyst dietary fibre | 13 | rs146445319 | T | C | 0.020 | -0.099 | 0.021 | 0.000 | 21.607 |
| Englyst dietary fibre | 15 | rs8039213 | C | T | 0.342 | 0.028 | 0.006 | 0.000 | 23.135 |
| Englyst dietary fibre | 18 | rs1433928 | C | T | 0.640 | -0.026 | 0.006 | 0.000 | 21.407 |
| Englyst dietary fibre | 22 | rs5746795 | G | A | 0.309 | -0.030 | 0.006 | 0.000 | 25.874 |
| Englyst dietary fibre | 22 | rs12165526 | A | T | 0.101 | 0.042 | 0.009 | 0.000 | 21.440 |
| Englyst dietary fibre | 22 | rs112995058 | A | G | 0.103 | 0.046 | 0.009 | 0.000 | 25.508 |
| Total cholesterol levels | 1 | rs11591147 | T | G | 0.018 | -0.327 | 0.008 | 0.000 | 1713.967 |
| Total cholesterol levels | 1 | rs3738621 | G | A | 0.213 | -0.019 | 0.002 | 0.000 | 56.605 |
| Total cholesterol levels | 1 | rs4971066 | G | T | 0.152 | 0.014 | 0.003 | 0.000 | 24.383 |
| Total cholesterol levels | 1 | rs11205362 | A | G | 0.120 | 0.015 | 0.003 | 0.000 | 23.239 |
| Total cholesterol levels | 1 | rs1795240 | G | A | 0.532 | -0.014 | 0.002 | 0.000 | 43.614 |
| Total cholesterol levels | 1 | rs7528419 | G | A | 0.222 | -0.102 | 0.002 | 0.000 | 1734.871 |
| Total cholesterol levels | 1 | rs6603979 | G | A | 0.794 | 0.021 | 0.003 | 0.000 | 66.580 |
| Total cholesterol levels | 1 | rs76272805 | A | G | 0.051 | -0.043 | 0.005 | 0.000 | 83.349 |
| Total cholesterol levels | 1 | rs526936 | A | G | 0.518 | 0.037 | 0.002 | 0.000 | 332.386 |
| Total cholesterol levels | 1 | rs10753556 | G | A | 0.875 | 0.021 | 0.003 | 0.000 | 48.197 |
| Total cholesterol levels | 1 | rs10888613 | G | C | 0.395 | -0.010 | 0.002 | 0.000 | 21.095 |
| Total cholesterol levels | 1 | rs17883304 | C | A | 0.166 | 0.013 | 0.003 | 0.000 | 21.220 |
| Total cholesterol levels | 1 | rs114165349 | C | G | 0.024 | 0.036 | 0.007 | 0.000 | 28.041 |
| Total cholesterol levels | 1 | rs1497406 | G | A | 0.579 | 0.012 | 0.002 | 0.000 | 35.180 |
| Total cholesterol levels | 1 | rs10735234 | A | G | 0.579 | -0.009 | 0.002 | 0.000 | 17.916 |
| Total cholesterol levels | 1 | rs12354278 | T | A | 0.130 | 0.015 | 0.003 | 0.000 | 23.831 |
| Total cholesterol levels | 1 | rs505151 | A | G | 0.958 | -0.068 | 0.006 | 0.000 | 135.712 |
| Total cholesterol levels | 1 | rs10919615 | T | C | 0.756 | 0.013 | 0.002 | 0.000 | 27.951 |
| Total cholesterol levels | 1 | rs12046278 | C | T | 0.345 | -0.016 | 0.002 | 0.000 | 53.833 |
| Total cholesterol levels | 1 | rs20563 | G | A | 0.567 | 0.011 | 0.002 | 0.000 | 27.835 |
| Total cholesterol levels | 1 | rs471705 | G | T | 0.639 | 0.040 | 0.002 | 0.000 | 344.750 |
| Total cholesterol levels | 1 | rs10903129 | G | A | 0.557 | 0.027 | 0.002 | 0.000 | 172.930 |
| Total cholesterol levels | 1 | rs140970775 | G | A | 0.073 | 0.023 | 0.004 | 0.000 | 33.119 |
| Total cholesterol levels | 1 | rs2642438 | G | A | 0.701 | 0.032 | 0.002 | 0.000 | 196.816 |
| Total cholesterol levels | 1 | rs17381154 | C | T | 0.027 | -0.053 | 0.006 | 0.000 | 68.897 |
| Total cholesterol levels | 1 | rs17036085 | G | A | 0.012 | -0.050 | 0.009 | 0.000 | 29.269 |
| Total cholesterol levels | 2 | rs780094 | C | T | 0.616 | -0.048 | 0.002 | 0.000 | 513.555 |
| Total cholesterol levels | 2 | rs964392 | G | A | 0.393 | 0.010 | 0.002 | 0.000 | 21.264 |
| Total cholesterol levels | 2 | rs114245489 | T | G | 0.064 | 0.027 | 0.004 | 0.000 | 38.615 |
| Total cholesterol levels | 2 | rs17050272 | A | G | 0.410 | -0.018 | 0.002 | 0.000 | 76.368 |
| Total cholesterol levels | 2 | rs17512204 | A | G | 0.083 | -0.034 | 0.004 | 0.000 | 84.586 |
| Total cholesterol levels | 2 | rs1250259 | A | T | 0.734 | 0.014 | 0.002 | 0.000 | 36.030 |
| Total cholesterol levels | 2 | rs887829 | T | C | 0.316 | -0.016 | 0.002 | 0.000 | 52.854 |
| Total cholesterol levels | 2 | rs2241340 | G | A | 0.594 | -0.022 | 0.002 | 0.000 | 103.334 |
| Total cholesterol levels | 2 | rs114813367 | T | C | 0.018 | -0.038 | 0.008 | 0.000 | 24.151 |
| Total cholesterol levels | 2 | rs11685443 | C | T | 0.241 | -0.010 | 0.002 | 0.000 | 18.670 |
| Total cholesterol levels | 2 | rs9306897 | C | T | 0.660 | -0.032 | 0.002 | 0.000 | 224.414 |
| Total cholesterol levels | 2 | rs562338 | G | A | 0.820 | 0.096 | 0.003 | 0.000 | 1316.041 |
| Total cholesterol levels | 2 | rs6734238 | G | A | 0.403 | -0.013 | 0.002 | 0.000 | 41.645 |
| Total cholesterol levels | 2 | rs4245791 | T | C | 0.676 | -0.054 | 0.002 | 0.000 | 605.359 |
| Total cholesterol levels | 2 | rs4850047 | C | T | 0.868 | -0.022 | 0.003 | 0.000 | 52.976 |
| Total cholesterol levels | 2 | rs3770710 | A | G | 0.162 | -0.014 | 0.003 | 0.000 | 24.321 |
| Total cholesterol levels | 2 | rs10189685 | A | G | 0.296 | -0.023 | 0.002 | 0.000 | 109.868 |
| Total cholesterol levels | 2 | rs7559334 | T | C | 0.119 | -0.016 | 0.003 | 0.000 | 22.919 |
| Total cholesterol levels | 2 | rs2539980 | C | T | 0.683 | 0.018 | 0.002 | 0.000 | 68.720 |
| Total cholesterol levels | 2 | rs17406264 | T | C | 0.019 | -0.033 | 0.007 | 0.000 | 20.267 |
| Total cholesterol levels | 2 | rs75865302 | T | C | 0.052 | 0.025 | 0.005 | 0.000 | 30.760 |
| Total cholesterol levels | 2 | rs2465520 | A | G | 0.113 | -0.021 | 0.003 | 0.000 | 39.976 |
| Total cholesterol levels | 2 | rs56383182 | G | A | 0.370 | 0.017 | 0.002 | 0.000 | 62.711 |
| Total cholesterol levels | 3 | rs9832727 | G | C | 0.340 | -0.016 | 0.002 | 0.000 | 54.728 |
| Total cholesterol levels | 3 | rs2305407 | A | G | 0.519 | -0.013 | 0.002 | 0.000 | 38.078 |
| Total cholesterol levels | 3 | rs2272471 | A | G | 0.478 | 0.009 | 0.002 | 0.000 | 17.264 |
| Total cholesterol levels | 3 | rs6785233 | G | T | 0.079 | 0.022 | 0.004 | 0.000 | 34.246 |
| Total cholesterol levels | 3 | rs2526385 | G | T | 0.804 | 0.016 | 0.003 | 0.000 | 40.460 |
| Total cholesterol levels | 3 | rs3732359 | A | G | 0.779 | -0.021 | 0.002 | 0.000 | 71.639 |
| Total cholesterol levels | 3 | rs6776900 | T | C | 0.143 | -0.013 | 0.003 | 0.000 | 20.207 |
| Total cholesterol levels | 3 | rs7640978 | T | C | 0.089 | -0.032 | 0.004 | 0.000 | 79.892 |
| Total cholesterol levels | 3 | rs9825431 | C | G | 0.083 | -0.032 | 0.004 | 0.000 | 73.804 |
| Total cholesterol levels | 3 | rs66513933 | C | T | 0.280 | -0.010 | 0.002 | 0.000 | 18.551 |
| Total cholesterol levels | 3 | rs78946096 | G | A | 0.057 | -0.041 | 0.004 | 0.000 | 86.531 |
| Total cholesterol levels | 3 | rs35939242 | C | A | 0.341 | -0.020 | 0.002 | 0.000 | 87.886 |
| Total cholesterol levels | 3 | rs830621 | T | C | 0.584 | 0.010 | 0.002 | 0.000 | 23.680 |
| Total cholesterol levels | 3 | rs28403550 | A | C | 0.159 | 0.019 | 0.003 | 0.000 | 46.177 |
| Total cholesterol levels | 4 | rs34664906 | T | C | 0.378 | 0.028 | 0.002 | 0.000 | 156.206 |
| Total cholesterol levels | 4 | rs13107325 | T | C | 0.075 | -0.041 | 0.004 | 0.000 | 105.303 |
| Total cholesterol levels | 4 | rs1062835 | G | C | 0.203 | 0.013 | 0.003 | 0.000 | 23.497 |
| Total cholesterol levels | 4 | rs172629 | G | C | 0.126 | -0.014 | 0.003 | 0.000 | 21.537 |
| Total cholesterol levels | 4 | rs1458038 | T | C | 0.292 | -0.017 | 0.002 | 0.000 | 56.748 |
| Total cholesterol levels | 4 | rs278981 | C | T | 0.758 | 0.012 | 0.002 | 0.000 | 23.937 |
| Total cholesterol levels | 4 | rs2705455 | G | A | 0.521 | -0.013 | 0.002 | 0.000 | 37.142 |
| Total cholesterol levels | 4 | rs6831256 | G | A | 0.423 | 0.017 | 0.002 | 0.000 | 68.119 |
| Total cholesterol levels | 4 | rs17879565 | G | A | 0.375 | 0.010 | 0.002 | 0.000 | 22.768 |
| Total cholesterol levels | 4 | rs1678299 | C | G | 0.730 | 0.014 | 0.002 | 0.000 | 33.149 |
| Total cholesterol levels | 4 | rs11737560 | T | C | 0.124 | -0.015 | 0.003 | 0.000 | 24.161 |
| Total cholesterol levels | 4 | rs11732093 | A | G | 0.357 | -0.011 | 0.002 | 0.000 | 24.384 |
| Total cholesterol levels | 4 | rs58148580 | T | C | 0.110 | 0.020 | 0.003 | 0.000 | 36.113 |
| Total cholesterol levels | 4 | rs28925904 | T | C | 0.025 | -0.035 | 0.007 | 0.000 | 27.459 |
| Total cholesterol levels | 5 | rs2074613 | T | C | 0.560 | 0.009 | 0.002 | 0.000 | 20.264 |
| Total cholesterol levels | 5 | rs13340362 | G | A | 0.370 | 0.009 | 0.002 | 0.000 | 19.446 |
| Total cholesterol levels | 5 | rs2706379 | T | C | 0.188 | -0.016 | 0.003 | 0.000 | 36.247 |
| Total cholesterol levels | 5 | rs35462600 | A | G | 0.030 | -0.027 | 0.006 | 0.000 | 20.510 |
| Total cholesterol levels | 5 | rs4530754 | A | G | 0.545 | 0.017 | 0.002 | 0.000 | 68.404 |
| Total cholesterol levels | 5 | rs4797 | A | G | 0.516 | -0.009 | 0.002 | 0.000 | 20.847 |
| Total cholesterol levels | 5 | rs77704739 | C | T | 0.041 | -0.036 | 0.005 | 0.000 | 47.040 |
| Total cholesterol levels | 5 | rs11950916 | G | C | 0.273 | 0.009 | 0.002 | 0.000 | 16.728 |
| Total cholesterol levels | 5 | rs1501908 | C | G | 0.635 | 0.040 | 0.002 | 0.000 | 352.668 |
| Total cholesterol levels | 5 | rs12916 | C | T | 0.400 | 0.062 | 0.002 | 0.000 | 865.513 |
| Total cholesterol levels | 6 | rs75962856 | C | T | 0.047 | -0.022 | 0.005 | 0.000 | 20.549 |
| Total cholesterol levels | 6 | rs74617384 | T | A | 0.079 | 0.078 | 0.004 | 0.000 | 415.536 |
| Total cholesterol levels | 6 | rs17412833 | T | A | 0.268 | 0.019 | 0.003 | 0.000 | 41.280 |
| Total cholesterol levels | 6 | rs11755266 | T | C | 0.094 | -0.035 | 0.004 | 0.000 | 100.092 |
| Total cholesterol levels | 6 | rs7752556 | A | G | 0.265 | 0.012 | 0.002 | 0.000 | 27.597 |
| Total cholesterol levels | 6 | rs2297367 | T | C | 0.044 | -0.026 | 0.005 | 0.000 | 26.074 |
| Total cholesterol levels | 6 | rs117733303 | G | A | 0.018 | 0.073 | 0.008 | 0.000 | 92.677 |
| Total cholesterol levels | 6 | rs12208357 | T | C | 0.070 | 0.053 | 0.004 | 0.000 | 172.217 |
| Total cholesterol levels | 6 | rs9352675 | A | G | 0.481 | -0.011 | 0.002 | 0.000 | 25.863 |
| Total cholesterol levels | 6 | rs1490384 | T | C | 0.500 | -0.009 | 0.002 | 0.000 | 21.180 |
| Total cholesterol levels | 6 | rs9405533 | G | A | 0.344 | -0.010 | 0.002 | 0.000 | 20.445 |
| Total cholesterol levels | 6 | rs9370867 | G | A | 0.462 | -0.020 | 0.002 | 0.000 | 89.678 |
| Total cholesterol levels | 6 | rs4712999 | G | A | 0.133 | -0.018 | 0.003 | 0.000 | 32.509 |
| Total cholesterol levels | 6 | rs7748291 | T | C | 0.501 | -0.014 | 0.002 | 0.000 | 48.813 |
| Total cholesterol levels | 6 | rs35211576 | G | A | 0.494 | -0.012 | 0.002 | 0.000 | 35.254 |
| Total cholesterol levels | 6 | rs17789218 | C | T | 0.245 | -0.016 | 0.002 | 0.000 | 47.081 |
| Total cholesterol levels | 6 | rs9376090 | C | T | 0.259 | -0.019 | 0.002 | 0.000 | 67.618 |
| Total cholesterol levels | 6 | rs3756772 | T | C | 0.400 | 0.023 | 0.002 | 0.000 | 117.760 |
| Total cholesterol levels | 6 | rs146534110 | T | G | 0.013 | 0.066 | 0.009 | 0.000 | 54.604 |
| Total cholesterol levels | 6 | rs113977268 | A | G | 0.086 | -0.016 | 0.004 | 0.000 | 17.944 |
| Total cholesterol levels | 6 | rs1800562 | A | G | 0.077 | -0.049 | 0.004 | 0.000 | 136.384 |
| Total cholesterol levels | 7 | rs55837101 | T | C | 0.229 | 0.012 | 0.002 | 0.000 | 24.415 |
| Total cholesterol levels | 7 | rs138491865 | A | G | 0.024 | 0.034 | 0.007 | 0.000 | 25.864 |
| Total cholesterol levels | 7 | rs66476925 | C | G | 0.216 | 0.033 | 0.003 | 0.000 | 172.029 |
| Total cholesterol levels | 7 | rs149795204 | A | G | 0.119 | -0.017 | 0.003 | 0.000 | 22.703 |
| Total cholesterol levels | 7 | rs2278130 | A | G | 0.263 | -0.011 | 0.002 | 0.000 | 22.262 |
| Total cholesterol levels | 7 | rs4374942 | C | T | 0.074 | 0.019 | 0.004 | 0.000 | 22.680 |
| Total cholesterol levels | 7 | rs799157 | C | T | 0.952 | -0.028 | 0.005 | 0.000 | 31.027 |
| Total cholesterol levels | 7 | rs2109505 | A | T | 0.179 | -0.015 | 0.003 | 0.000 | 31.928 |
| Total cholesterol levels | 7 | rs2073547 | G | A | 0.184 | 0.033 | 0.003 | 0.000 | 156.811 |
| Total cholesterol levels | 7 | rs1708299 | G | A | 0.702 | 0.011 | 0.002 | 0.000 | 25.028 |
| Total cholesterol levels | 7 | rs1057868 | T | C | 0.283 | 0.013 | 0.002 | 0.000 | 33.497 |
| Total cholesterol levels | 7 | rs12666989 | C | G | 0.182 | 0.022 | 0.003 | 0.000 | 65.879 |
| Total cholesterol levels | 7 | rs3808348 | T | C | 0.205 | -0.017 | 0.003 | 0.000 | 45.750 |
| Total cholesterol levels | 7 | rs1524776 | A | C | 0.506 | -0.012 | 0.002 | 0.000 | 33.014 |
| Total cholesterol levels | 7 | rs2288153 | T | C | 0.238 | 0.021 | 0.002 | 0.000 | 72.041 |
| Total cholesterol levels | 8 | rs179442 | T | C | 0.338 | -0.019 | 0.002 | 0.000 | 74.739 |
| Total cholesterol levels | 8 | rs11136343 | G | A | 0.376 | 0.014 | 0.002 | 0.000 | 39.457 |
| Total cholesterol levels | 8 | rs4841132 | G | A | 0.909 | 0.081 | 0.004 | 0.000 | 460.297 |
| Total cholesterol levels | 8 | rs2928619 | T | C | 0.654 | 0.014 | 0.002 | 0.000 | 39.859 |
| Total cholesterol levels | 8 | rs6474359 | C | T | 0.038 | -0.031 | 0.005 | 0.000 | 33.617 |
| Total cholesterol levels | 8 | rs2081687 | C | T | 0.663 | -0.032 | 0.002 | 0.000 | 224.019 |
| Total cholesterol levels | 8 | rs2306486 | T | C | 0.317 | -0.011 | 0.002 | 0.000 | 24.514 |
| Total cholesterol levels | 8 | rs2875973 | T | C | 0.371 | 0.014 | 0.002 | 0.000 | 44.891 |
| Total cholesterol levels | 8 | rs17526980 | T | C | 0.039 | 0.027 | 0.005 | 0.000 | 25.747 |
| Total cholesterol levels | 8 | rs2375549 | A | G | 0.393 | -0.009 | 0.002 | 0.000 | 19.906 |
| Total cholesterol levels | 8 | rs1041983 | T | C | 0.318 | -0.012 | 0.002 | 0.000 | 30.042 |
| Total cholesterol levels | 8 | rs10104187 | C | T | 0.557 | 0.012 | 0.002 | 0.000 | 31.890 |
| Total cholesterol levels | 8 | rs2001945 | C | G | 0.520 | -0.053 | 0.002 | 0.000 | 667.795 |
| Total cholesterol levels | 8 | rs330089 | C | T | 0.098 | 0.022 | 0.003 | 0.000 | 39.847 |
| Total cholesterol levels | 8 | rs117139027 | A | G | 0.017 | -0.059 | 0.008 | 0.000 | 54.322 |
| Total cholesterol levels | 9 | rs3748176 | A | G | 0.534 | 0.011 | 0.002 | 0.000 | 28.308 |
| Total cholesterol levels | 9 | rs2297409 | A | G | 0.194 | -0.019 | 0.003 | 0.000 | 52.201 |
| Total cholesterol levels | 9 | rs1883025 | T | C | 0.254 | -0.050 | 0.002 | 0.000 | 448.790 |
| Total cholesterol levels | 9 | rs581080 | C | G | 0.819 | 0.025 | 0.003 | 0.000 | 88.754 |
| Total cholesterol levels | 9 | rs10870160 | T | C | 0.279 | -0.012 | 0.002 | 0.000 | 24.833 |
| Total cholesterol levels | 9 | rs6475606 | T | C | 0.484 | -0.020 | 0.002 | 0.000 | 96.205 |
| Total cholesterol levels | 9 | rs7035578 | A | G | 0.156 | -0.013 | 0.003 | 0.000 | 22.495 |
| Total cholesterol levels | 9 | rs507666 | A | G | 0.185 | 0.056 | 0.003 | 0.000 | 441.757 |
| Total cholesterol levels | 9 | rs12336893 | G | T | 0.054 | -0.025 | 0.005 | 0.000 | 31.432 |
| Total cholesterol levels | 9 | rs2066714 | C | T | 0.127 | 0.035 | 0.003 | 0.000 | 124.369 |
| Total cholesterol levels | 9 | rs72760251 | G | T | 0.095 | 0.017 | 0.004 | 0.000 | 22.794 |
| Total cholesterol levels | 10 | rs2862954 | C | T | 0.496 | 0.019 | 0.002 | 0.000 | 83.362 |
| Total cholesterol levels | 10 | rs7921838 | A | G | 0.153 | -0.019 | 0.003 | 0.000 | 45.307 |
| Total cholesterol levels | 10 | rs2068888 | A | G | 0.451 | -0.016 | 0.002 | 0.000 | 63.670 |
| Total cholesterol levels | 10 | rs10794579 | C | T | 0.575 | 0.017 | 0.002 | 0.000 | 63.659 |
| Total cholesterol levels | 10 | rs7920112 | C | T | 0.418 | 0.017 | 0.002 | 0.000 | 67.825 |
| Total cholesterol levels | 10 | rs2883280 | T | C | 0.297 | -0.011 | 0.002 | 0.000 | 25.150 |
| Total cholesterol levels | 10 | rs970548 | C | A | 0.247 | 0.018 | 0.002 | 0.000 | 57.820 |
| Total cholesterol levels | 10 | rs2250781 | A | C | 0.503 | -0.009 | 0.002 | 0.000 | 17.044 |
| Total cholesterol levels | 10 | rs11006229 | T | C | 0.199 | 0.013 | 0.003 | 0.000 | 26.450 |
| Total cholesterol levels | 10 | rs2792751 | C | T | 0.721 | -0.025 | 0.002 | 0.000 | 116.630 |
| Total cholesterol levels | 10 | rs7476477 | G | A | 0.484 | 0.011 | 0.002 | 0.000 | 30.534 |
| Total cholesterol levels | 10 | rs16926246 | T | C | 0.130 | -0.024 | 0.003 | 0.000 | 62.913 |
| Total cholesterol levels | 10 | rs41314509 | A | G | 0.172 | -0.016 | 0.003 | 0.000 | 32.563 |
| Total cholesterol levels | 11 | rs10892881 | A | C | 0.246 | 0.014 | 0.002 | 0.000 | 35.636 |
| Total cholesterol levels | 11 | rs7124487 | T | C | 0.204 | -0.017 | 0.003 | 0.000 | 42.640 |
| Total cholesterol levels | 11 | rs4752805 | G | A | 0.253 | 0.021 | 0.002 | 0.000 | 77.640 |
| Total cholesterol levels | 11 | rs11226108 | C | G | 0.189 | -0.020 | 0.003 | 0.000 | 56.079 |
| Total cholesterol levels | 11 | rs11821808 | T | C | 0.094 | -0.025 | 0.004 | 0.000 | 49.174 |
| Total cholesterol levels | 11 | rs11601507 | A | C | 0.068 | 0.026 | 0.004 | 0.000 | 39.089 |
| Total cholesterol levels | 11 | rs12970 | A | G | 0.060 | -0.033 | 0.004 | 0.000 | 58.059 |
| Total cholesterol levels | 11 | rs10128711 | C | T | 0.733 | 0.018 | 0.002 | 0.000 | 57.671 |
| Total cholesterol levels | 11 | rs12575519 | G | T | 0.170 | -0.016 | 0.003 | 0.000 | 32.483 |
| Total cholesterol levels | 11 | rs10896018 | A | G | 0.287 | 0.013 | 0.002 | 0.000 | 30.180 |
| Total cholesterol levels | 11 | rs964184 | C | G | 0.867 | -0.072 | 0.003 | 0.000 | 567.650 |
| Total cholesterol levels | 11 | rs174550 | C | T | 0.344 | -0.047 | 0.002 | 0.000 | 470.211 |
| Total cholesterol levels | 11 | rs11220462 | A | G | 0.133 | 0.032 | 0.003 | 0.000 | 115.891 |
| Total cholesterol levels | 12 | rs1521516 | T | C | 0.345 | -0.020 | 0.002 | 0.000 | 86.348 |
| Total cholesterol levels | 12 | rs11045247 | A | G | 0.073 | -0.019 | 0.004 | 0.000 | 23.897 |
| Total cholesterol levels | 12 | rs9668810 | C | T | 0.736 | -0.013 | 0.002 | 0.000 | 29.806 |
| Total cholesterol levels | 12 | rs10876171 | A | G | 0.638 | -0.011 | 0.002 | 0.000 | 25.995 |
| Total cholesterol levels | 12 | rs61754230 | T | C | 0.019 | 0.043 | 0.008 | 0.000 | 30.623 |
| Total cholesterol levels | 12 | rs1169288 | C | A | 0.315 | 0.029 | 0.002 | 0.000 | 165.892 |
| Total cholesterol levels | 12 | rs11057273 | C | T | 0.907 | -0.029 | 0.004 | 0.000 | 68.050 |
| Total cholesterol levels | 12 | rs56244055 | A | G | 0.180 | 0.017 | 0.003 | 0.000 | 40.778 |
| Total cholesterol levels | 12 | rs4565995 | C | T | 0.909 | 0.021 | 0.004 | 0.000 | 35.194 |
| Total cholesterol levels | 12 | rs804667 | T | C | 0.297 | 0.011 | 0.002 | 0.000 | 21.651 |
| Total cholesterol levels | 12 | rs653178 | T | C | 0.516 | 0.034 | 0.002 | 0.000 | 268.534 |
| Total cholesterol levels | 12 | rs76191003 | T | C | 0.068 | 0.020 | 0.004 | 0.000 | 25.170 |
| Total cholesterol levels | 12 | rs7980026 | C | T | 0.147 | 0.016 | 0.003 | 0.000 | 28.561 |
| Total cholesterol levels | 12 | rs4765611 | G | A | 0.469 | -0.017 | 0.002 | 0.000 | 70.806 |
| Total cholesterol levels | 12 | rs11614702 | A | G | 0.512 | 0.010 | 0.002 | 0.000 | 21.492 |
| Total cholesterol levels | 12 | rs35882350 | G | A | 0.261 | 0.013 | 0.002 | 0.000 | 32.436 |
| Total cholesterol levels | 12 | rs11568544 | G | A | 0.366 | -0.011 | 0.002 | 0.000 | 28.392 |
| Total cholesterol levels | 13 | rs9534262 | C | T | 0.516 | -0.015 | 0.002 | 0.000 | 51.107 |
| Total cholesterol levels | 13 | rs9577924 | G | A | 0.262 | 0.025 | 0.002 | 0.000 | 115.958 |
| Total cholesterol levels | 13 | rs4885161 | G | A | 0.907 | 0.018 | 0.004 | 0.000 | 25.043 |
| Total cholesterol levels | 13 | rs1329520 | G | A | 0.301 | -0.010 | 0.002 | 0.000 | 21.921 |
| Total cholesterol levels | 13 | rs9506722 | G | A | 0.390 | 0.009 | 0.002 | 0.000 | 18.300 |
| Total cholesterol levels | 13 | rs7991128 | G | T | 0.244 | 0.012 | 0.002 | 0.000 | 25.868 |
| Total cholesterol levels | 13 | rs9604529 | G | A | 0.190 | -0.013 | 0.003 | 0.000 | 23.038 |
| Total cholesterol levels | 14 | rs7157785 | T | G | 0.161 | 0.018 | 0.003 | 0.000 | 40.763 |
| Total cholesterol levels | 14 | rs2877660 | G | A | 0.861 | 0.014 | 0.003 | 0.000 | 23.379 |
| Total cholesterol levels | 14 | rs3087686 | G | A | 0.198 | -0.013 | 0.003 | 0.000 | 25.409 |
| Total cholesterol levels | 14 | rs2494748 | T | C | 0.610 | -0.011 | 0.002 | 0.000 | 24.195 |
| Total cholesterol levels | 14 | rs34752362 | A | G | 0.446 | -0.014 | 0.002 | 0.000 | 46.734 |
| Total cholesterol levels | 14 | rs17580 | A | T | 0.048 | 0.033 | 0.005 | 0.000 | 44.964 |
| Total cholesterol levels | 14 | rs8017377 | A | G | 0.470 | 0.017 | 0.002 | 0.000 | 64.200 |
| Total cholesterol levels | 15 | rs1532085 | G | A | 0.614 | -0.052 | 0.002 | 0.000 | 601.135 |
| Total cholesterol levels | 15 | rs34862454 | T | C | 0.669 | 0.016 | 0.002 | 0.000 | 51.530 |
| Total cholesterol levels | 15 | rs6416553 | T | C | 0.849 | -0.016 | 0.003 | 0.000 | 31.346 |
| Total cholesterol levels | 15 | rs12903114 | T | C | 0.393 | 0.011 | 0.002 | 0.000 | 28.321 |
| Total cholesterol levels | 15 | rs1800588 | T | C | 0.216 | 0.063 | 0.002 | 0.000 | 638.628 |
| Total cholesterol levels | 15 | rs116863821 | G | T | 0.026 | 0.035 | 0.007 | 0.000 | 28.726 |
| Total cholesterol levels | 16 | rs4328458 | A | G | 0.444 | 0.010 | 0.002 | 0.000 | 24.725 |
| Total cholesterol levels | 16 | rs34830321 | T | C | 0.015 | -0.039 | 0.009 | 0.000 | 20.298 |
| Total cholesterol levels | 16 | rs3887592 | C | T | 0.434 | -0.010 | 0.002 | 0.000 | 21.298 |
| Total cholesterol levels | 16 | rs7186852 | G | A | 0.359 | 0.012 | 0.002 | 0.000 | 31.397 |
| Total cholesterol levels | 16 | rs3764261 | A | C | 0.324 | 0.044 | 0.002 | 0.000 | 399.643 |
| Total cholesterol levels | 16 | rs12931135 | G | A | 0.313 | -0.012 | 0.002 | 0.000 | 28.526 |
| Total cholesterol levels | 16 | rs8050136 | A | C | 0.395 | -0.012 | 0.002 | 0.000 | 33.504 |
| Total cholesterol levels | 16 | rs2255437 | G | A | 0.527 | -0.014 | 0.002 | 0.000 | 48.974 |
| Total cholesterol levels | 16 | rs56313425 | G | A | 0.026 | -0.032 | 0.007 | 0.000 | 24.560 |
| Total cholesterol levels | 16 | rs4788815 | T | A | 0.657 | 0.020 | 0.002 | 0.000 | 85.661 |
| Total cholesterol levels | 16 | rs2000999 | A | G | 0.172 | 0.050 | 0.003 | 0.000 | 315.992 |
| Total cholesterol levels | 16 | rs12444979 | T | C | 0.143 | 0.016 | 0.003 | 0.000 | 30.824 |
| Total cholesterol levels | 17 | rs55714927 | T | C | 0.190 | -0.025 | 0.003 | 0.000 | 89.936 |
| Total cholesterol levels | 17 | rs72631343 | G | C | 0.129 | -0.028 | 0.003 | 0.000 | 81.593 |
| Total cholesterol levels | 17 | rs2290771 | G | A | 0.311 | -0.012 | 0.002 | 0.000 | 28.503 |
| Total cholesterol levels | 17 | rs17880847 | A | T | 0.014 | 0.038 | 0.009 | 0.000 | 18.127 |
| Total cholesterol levels | 17 | rs9916193 | G | C | 0.196 | 0.015 | 0.003 | 0.000 | 32.535 |
| Total cholesterol levels | 17 | rs9907571 | A | G | 0.306 | 0.012 | 0.002 | 0.000 | 30.187 |
| Total cholesterol levels | 17 | rs12452315 | C | A | 0.481 | 0.024 | 0.002 | 0.000 | 133.594 |
| Total cholesterol levels | 17 | rs2905880 | C | T | 0.696 | 0.017 | 0.002 | 0.000 | 55.532 |
| Total cholesterol levels | 17 | rs72836561 | T | C | 0.031 | -0.048 | 0.006 | 0.000 | 65.451 |
| Total cholesterol levels | 17 | rs1801689 | C | A | 0.031 | 0.050 | 0.006 | 0.000 | 69.180 |
| Total cholesterol levels | 17 | rs8065251 | A | G | 0.217 | 0.015 | 0.003 | 0.000 | 32.287 |
| Total cholesterol levels | 17 | rs9890198 | C | A | 0.157 | -0.013 | 0.003 | 0.000 | 21.767 |
| Total cholesterol levels | 17 | rs7216657 | A | G | 0.066 | -0.025 | 0.004 | 0.000 | 33.494 |
| Total cholesterol levels | 17 | rs55784804 | T | G | 0.099 | 0.019 | 0.004 | 0.000 | 28.925 |
| Total cholesterol levels | 17 | rs77542162 | G | A | 0.023 | 0.109 | 0.007 | 0.000 | 241.566 |
| Total cholesterol levels | 17 | rs2292641 | T | C | 0.147 | -0.025 | 0.003 | 0.000 | 74.226 |
| Total cholesterol levels | 18 | rs118057319 | C | T | 0.055 | -0.029 | 0.005 | 0.000 | 40.437 |
| Total cholesterol levels | 18 | rs2298624 | T | C | 0.132 | 0.019 | 0.003 | 0.000 | 38.024 |
| Total cholesterol levels | 18 | rs17773430 | C | T | 0.312 | -0.010 | 0.002 | 0.000 | 19.189 |
| Total cholesterol levels | 18 | rs402348 | G | T | 0.191 | -0.012 | 0.003 | 0.000 | 21.589 |
| Total cholesterol levels | 18 | rs4939883 | C | T | 0.820 | 0.046 | 0.003 | 0.000 | 295.332 |
| Total cholesterol levels | 18 | rs77960347 | G | A | 0.013 | 0.176 | 0.009 | 0.000 | 374.747 |
| Total cholesterol levels | 18 | rs4468717 | T | C | 0.077 | 0.021 | 0.004 | 0.000 | 26.580 |
| Total cholesterol levels | 18 | rs45450798 | G | C | 0.167 | -0.012 | 0.003 | 0.000 | 19.141 |
| Total cholesterol levels | 19 | rs2738447 | C | A | 0.589 | 0.037 | 0.002 | 0.000 | 312.131 |
| Total cholesterol levels | 19 | rs34529039 | A | C | 0.137 | -0.017 | 0.003 | 0.000 | 32.875 |
| Total cholesterol levels | 19 | rs117326714 | G | A | 0.026 | -0.031 | 0.006 | 0.000 | 23.091 |
| Total cholesterol levels | 19 | rs676388 | C | T | 0.533 | 0.031 | 0.002 | 0.000 | 225.272 |
| Total cholesterol levels | 19 | rs2278426 | T | C | 0.035 | -0.078 | 0.006 | 0.000 | 191.191 |
| Total cholesterol levels | 19 | rs79429216 | A | G | 0.012 | 0.133 | 0.010 | 0.000 | 192.945 |
| Total cholesterol levels | 19 | rs74607435 | C | T | 0.051 | -0.049 | 0.005 | 0.000 | 113.324 |
| Total cholesterol levels | 19 | rs7254892 | A | G | 0.032 | -0.321 | 0.006 | 0.000 | 3046.815 |
| Total cholesterol levels | 19 | rs2302491 | C | A | 0.209 | 0.013 | 0.003 | 0.000 | 28.292 |
| Total cholesterol levels | 19 | rs139425456 | A | T | 0.018 | 0.066 | 0.008 | 0.000 | 65.188 |
| Total cholesterol levels | 19 | rs76738189 | C | T | 0.030 | 0.035 | 0.006 | 0.000 | 34.655 |
| Total cholesterol levels | 19 | rs117142879 | T | C | 0.020 | 0.034 | 0.007 | 0.000 | 21.327 |
| Total cholesterol levels | 19 | rs55791371 | C | A | 0.118 | -0.160 | 0.003 | 0.000 | 2567.814 |
| Total cholesterol levels | 19 | rs2062251 | T | G | 0.275 | -0.014 | 0.002 | 0.000 | 37.164 |
| Total cholesterol levels | 19 | rs58542926 | T | C | 0.074 | -0.125 | 0.004 | 0.000 | 1002.193 |
| Total cholesterol levels | 19 | rs62117160 | A | G | 0.045 | -0.255 | 0.005 | 0.000 | 2727.380 |
| Total cholesterol levels | 20 | rs2207132 | A | G | 0.033 | 0.094 | 0.006 | 0.000 | 271.030 |
| Total cholesterol levels | 20 | rs1800961 | T | C | 0.031 | -0.098 | 0.006 | 0.000 | 261.745 |
| Total cholesterol levels | 20 | rs1885163 | C | T | 0.299 | 0.013 | 0.002 | 0.000 | 33.630 |
| Total cholesterol levels | 20 | rs2236510 | T | C | 0.243 | 0.017 | 0.002 | 0.000 | 48.102 |
| Total cholesterol levels | 20 | rs6062359 | G | T | 0.442 | 0.010 | 0.002 | 0.000 | 23.244 |
| Total cholesterol levels | 20 | rs2618566 | T | G | 0.660 | -0.023 | 0.002 | 0.000 | 116.060 |
| Total cholesterol levels | 20 | rs432266 | C | T | 0.671 | -0.011 | 0.002 | 0.000 | 25.120 |
| Total cholesterol levels | 20 | rs6124298 | A | G | 0.294 | 0.018 | 0.002 | 0.000 | 64.297 |
| Total cholesterol levels | 20 | rs6013844 | C | T | 0.514 | 0.011 | 0.002 | 0.000 | 27.620 |
| Total cholesterol levels | 20 | rs6115094 | G | A | 0.493 | 0.013 | 0.002 | 0.000 | 38.665 |
| Total cholesterol levels | 20 | rs6060535 | T | C | 0.101 | -0.025 | 0.003 | 0.000 | 51.640 |
| Total cholesterol levels | 20 | rs364585 | G | A | 0.609 | 0.013 | 0.002 | 0.000 | 39.041 |
| Total cholesterol levels | 21 | rs116986306 | T | G | 0.026 | -0.029 | 0.006 | 0.000 | 20.774 |
| Total cholesterol levels | 21 | rs2835314 | G | A | 0.579 | -0.010 | 0.002 | 0.000 | 25.142 |
| Total cholesterol levels | 21 | rs11088472 | C | A | 0.571 | 0.012 | 0.002 | 0.000 | 31.862 |
| Total cholesterol levels | 22 | rs5754217 | T | G | 0.192 | -0.024 | 0.003 | 0.000 | 87.427 |
| Total cholesterol levels | 22 | rs138354 | C | T | 0.534 | -0.012 | 0.002 | 0.000 | 35.989 |
| Total cholesterol levels | 22 | rs4253778 | C | G | 0.180 | 0.013 | 0.003 | 0.000 | 25.341 |
| Total cholesterol levels | 22 | rs17657174 | G | C | 0.235 | -0.015 | 0.002 | 0.000 | 34.848 |
| Total cholesterol levels | 22 | rs12162782 | G | T | 0.344 | 0.013 | 0.002 | 0.000 | 36.735 |
| Caffeine levels | 1 | rs1495238 | A | C | 0.033 | -0.698 | 0.142 | 0.000 | 23.909 |
| Caffeine levels | 1 | rs4073576 | A | G | 0.473 | -0.245 | 0.049 | 0.000 | 24.328 |
| Caffeine levels | 2 | rs72782813 | A | G | 0.214 | 0.318 | 0.059 | 0.000 | 29.201 |
| Caffeine levels | 3 | rs1013382 | A | G | 0.511 | -0.257 | 0.054 | 0.000 | 22.832 |
| Caffeine levels | 5 | rs4409101 | A | G | 0.524 | -0.226 | 0.049 | 0.000 | 21.269 |
| Caffeine levels | 6 | rs1742932 | A | G | 0.683 | -0.252 | 0.054 | 0.000 | 21.577 |
| Caffeine levels | 7 | rs74790667 | T | C | 0.050 | -0.532 | 0.114 | 0.000 | 21.453 |
| Caffeine levels | 7 | rs2699454 | T | C | 0.726 | 0.275 | 0.058 | 0.000 | 22.173 |
| Caffeine levels | 7 | rs7779578 | A | C | 0.093 | -0.429 | 0.093 | 0.000 | 20.997 |
| Caffeine levels | 8 | rs3863243 | A | G | 0.334 | 0.238 | 0.049 | 0.000 | 23.236 |
| Caffeine levels | 8 | rs7011559 | G | A | 0.156 | -0.343 | 0.071 | 0.000 | 22.981 |
| Caffeine levels | 9 | rs10991955 | A | G | 0.026 | -0.736 | 0.160 | 0.000 | 21.052 |
| Caffeine levels | 10 | rs2393949 | C | T | 0.684 | 0.276 | 0.052 | 0.000 | 27.911 |
| Caffeine levels | 12 | rs7485945 | A | G | 0.983 | 1.068 | 0.223 | 0.000 | 22.874 |
| Caffeine levels | 13 | rs3783079 | C | T | 0.075 | -0.485 | 0.093 | 0.000 | 26.812 |
| Caffeine levels | 17 | rs4793951 | C | T | 0.185 | -0.309 | 0.067 | 0.000 | 21.250 |
| Caffeine levels | 18 | rs74537464 | A | G | 0.060 | -0.539 | 0.116 | 0.000 | 21.474 |
| Energy | 1 | rs111893801 | T | C | 0.062 | -0.053 | 0.011 | 0.000 | 22.063 |
| Energy | 2 | rs546217 | C | A | 0.430 | 0.025 | 0.005 | 0.000 | 21.618 |
| Energy | 4 | rs11132733 | C | T | 0.816 | -0.032 | 0.007 | 0.000 | 22.725 |
| Energy | 5 | rs35237101 | G | A | 0.042 | 0.066 | 0.014 | 0.000 | 23.683 |
| Energy | 5 | rs62347998 | T | C | 0.042 | 0.069 | 0.013 | 0.000 | 26.125 |
| Energy | 6 | rs149006866 | G | C | 0.025 | 0.091 | 0.018 | 0.000 | 26.758 |
| Energy | 6 | rs12528608 | T | G | 0.214 | 0.033 | 0.006 | 0.000 | 25.891 |
| Energy | 7 | rs10271291 | C | G | 0.240 | 0.028 | 0.006 | 0.000 | 20.860 |
| Energy | 8 | rs4532636 | C | A | 0.557 | 0.026 | 0.005 | 0.000 | 23.284 |
| Energy | 10 | rs7911565 | A | T | 0.023 | 0.082 | 0.018 | 0.000 | 21.296 |
| Energy | 11 | rs752690 | G | C | 0.661 | -0.026 | 0.006 | 0.000 | 21.160 |
| Energy | 11 | rs11224098 | G | A | 0.144 | -0.039 | 0.008 | 0.000 | 27.096 |
| Energy | 21 | rs13048538 | G | A | 0.239 | -0.030 | 0.006 | 0.000 | 23.349 |
| Alcoholic drinks | 1 | rs823152 | A | G | 0.416 | 0.010 | 0.002 | 0.000 | 24.700 |
| Alcoholic drinks | 1 | rs61836129 | C | T | 0.012 | -0.044 | 0.009 | 0.000 | 23.600 |
| Alcoholic drinks | 1 | rs7547690 | C | T | 0.702 | -0.011 | 0.002 | 0.000 | 29.500 |
| Alcoholic drinks | 1 | rs28680958 | A | G | 0.230 | -0.014 | 0.002 | 0.000 | 32.900 |
| Alcoholic drinks | 1 | rs34305371 | A | G | 0.086 | -0.017 | 0.003 | 0.000 | 25.800 |
| Alcoholic drinks | 1 | rs79072804 | G | A | 0.013 | 0.037 | 0.008 | 0.000 | 21.600 |
| Alcoholic drinks | 1 | rs58107686 | A | C | 0.344 | -0.011 | 0.002 | 0.000 | 28.300 |
| Alcoholic drinks | 1 | rs2854459 | C | T | 0.795 | 0.011 | 0.002 | 0.000 | 21.300 |
| Alcoholic drinks | 1 | rs1416706 | A | G | 0.672 | 0.010 | 0.002 | 0.000 | 23.400 |
| Alcoholic drinks | 2 | rs2972140 | C | T | 0.654 | -0.009 | 0.002 | 0.000 | 22.100 |
| Alcoholic drinks | 2 | rs1004787 | A | G | 0.581 | 0.015 | 0.002 | 0.000 | 62.100 |
| Alcoholic drinks | 2 | rs13024996 | A | C | 0.352 | -0.012 | 0.002 | 0.000 | 35.900 |
| Alcoholic drinks | 2 | rs75750180 | T | G | 0.093 | -0.015 | 0.003 | 0.000 | 21.800 |
| Alcoholic drinks | 2 | rs62135521 | T | G | 0.038 | -0.026 | 0.005 | 0.000 | 32.900 |
| Alcoholic drinks | 2 | rs1377491 | T | A | 0.807 | 0.012 | 0.002 | 0.000 | 23.900 |
| Alcoholic drinks | 2 | rs72857537 | C | T | 0.039 | 0.023 | 0.005 | 0.000 | 20.900 |
| Alcoholic drinks | 2 | rs4664531 | C | T | 0.278 | -0.010 | 0.002 | 0.000 | 21.800 |
| Alcoholic drinks | 2 | rs10184268 | T | C | 0.239 | 0.011 | 0.002 | 0.000 | 25.100 |
| Alcoholic drinks | 2 | rs6739804 | C | T | 0.660 | -0.013 | 0.002 | 0.000 | 38.800 |
| Alcoholic drinks | 2 | rs1260326 | C | T | 0.595 | 0.024 | 0.002 | 0.000 | 144.000 |
| Alcoholic drinks | 2 | rs56337305 | C | T | 0.375 | -0.010 | 0.002 | 0.000 | 25.800 |
| Alcoholic drinks | 2 | rs78876991 | C | T | 0.059 | -0.021 | 0.004 | 0.000 | 26.100 |
| Alcoholic drinks | 3 | rs9809760 | C | T | 0.470 | 0.009 | 0.002 | 0.000 | 22.600 |
| Alcoholic drinks | 3 | rs3732869 | A | T | 0.069 | -0.020 | 0.004 | 0.000 | 25.400 |
| Alcoholic drinks | 3 | rs7618629 | G | A | 0.772 | 0.011 | 0.002 | 0.000 | 22.700 |
| Alcoholic drinks | 3 | rs28732378 | G | A | 0.729 | -0.017 | 0.002 | 0.000 | 58.300 |
| Alcoholic drinks | 3 | rs6787172 | G | T | 0.546 | -0.011 | 0.002 | 0.000 | 29.500 |
| Alcoholic drinks | 3 | rs60026303 | G | A | 0.187 | 0.011 | 0.002 | 0.000 | 21.500 |
| Alcoholic drinks | 3 | rs79373562 | A | G | 0.024 | -0.029 | 0.006 | 0.000 | 24.900 |
| Alcoholic drinks | 3 | rs6809836 | A | G | 0.294 | -0.010 | 0.002 | 0.000 | 24.200 |
| Alcoholic drinks | 4 | rs331939 | A | G | 0.339 | -0.012 | 0.002 | 0.000 | 34.400 |
| Alcoholic drinks | 4 | rs1229984 | C | T | 0.953 | 0.188 | 0.006 | 0.000 | 926.993 |
| Alcoholic drinks | 4 | rs78234152 | A | G | 0.099 | 0.028 | 0.003 | 0.000 | 81.100 |
| Alcoholic drinks | 4 | rs12646808 | C | T | 0.338 | -0.011 | 0.002 | 0.000 | 26.800 |
| Alcoholic drinks | 4 | rs28712821 | A | G | 0.594 | 0.028 | 0.002 | 0.000 | 206.001 |
| Alcoholic drinks | 4 | rs16854020 | A | G | 0.127 | 0.018 | 0.003 | 0.000 | 38.700 |
| Alcoholic drinks | 4 | rs28694391 | C | T | 0.804 | -0.013 | 0.003 | 0.000 | 28.100 |
| Alcoholic drinks | 4 | rs13124335 | C | T | 0.174 | 0.012 | 0.003 | 0.000 | 22.200 |
| Alcoholic drinks | 4 | rs2074378 | T | C | 0.341 | -0.010 | 0.002 | 0.000 | 23.700 |
| Alcoholic drinks | 4 | rs7661710 | G | A | 0.487 | 0.009 | 0.002 | 0.000 | 22.700 |
| Alcoholic drinks | 4 | rs13107325 | T | C | 0.065 | -0.036 | 0.004 | 0.000 | 86.800 |
| Alcoholic drinks | 4 | rs1383346 | A | G | 0.462 | -0.009 | 0.002 | 0.000 | 21.700 |
| Alcoholic drinks | 5 | rs12513758 | G | C | 0.161 | -0.014 | 0.003 | 0.000 | 26.600 |
| Alcoholic drinks | 5 | rs2545799 | G | T | 0.508 | 0.009 | 0.002 | 0.000 | 21.400 |
| Alcoholic drinks | 5 | rs13179781 | G | A | 0.573 | -0.009 | 0.002 | 0.000 | 20.900 |
| Alcoholic drinks | 5 | rs2194027 | A | T | 0.453 | -0.010 | 0.002 | 0.000 | 29.200 |
| Alcoholic drinks | 5 | rs4501371 | A | T | 0.521 | -0.010 | 0.002 | 0.000 | 28.000 |
| Alcoholic drinks | 5 | rs10513154 | C | T | 0.267 | 0.012 | 0.002 | 0.000 | 28.300 |
| Alcoholic drinks | 5 | rs55786063 | T | G | 0.469 | -0.009 | 0.002 | 0.000 | 23.400 |
| Alcoholic drinks | 5 | rs4481304 | A | G | 0.437 | -0.009 | 0.002 | 0.000 | 22.300 |
| Alcoholic drinks | 5 | rs55872084 | T | G | 0.218 | 0.013 | 0.002 | 0.000 | 31.500 |
| Alcoholic drinks | 6 | rs1906252 | A | C | 0.466 | 0.009 | 0.002 | 0.000 | 21.200 |
| Alcoholic drinks | 6 | rs911475 | T | C | 0.153 | -0.013 | 0.003 | 0.000 | 23.200 |
| Alcoholic drinks | 6 | rs9349379 | G | A | 0.399 | 0.009 | 0.002 | 0.000 | 20.900 |
| Alcoholic drinks | 7 | rs10085696 | G | A | 0.201 | -0.016 | 0.002 | 0.000 | 41.400 |
| Alcoholic drinks | 7 | rs11238438 | C | G | 0.408 | 0.009 | 0.002 | 0.000 | 22.400 |
| Alcoholic drinks | 7 | rs78697684 | A | G | 0.017 | -0.033 | 0.007 | 0.000 | 23.300 |
| Alcoholic drinks | 7 | rs6962879 | G | C | 0.599 | 0.010 | 0.002 | 0.000 | 23.600 |
| Alcoholic drinks | 7 | rs2299409 | A | G | 0.493 | -0.011 | 0.002 | 0.000 | 29.800 |
| Alcoholic drinks | 7 | rs10236149 | G | A | 0.170 | -0.016 | 0.003 | 0.000 | 28.700 |
| Alcoholic drinks | 7 | rs6969458 | A | G | 0.459 | 0.013 | 0.002 | 0.000 | 43.100 |
| Alcoholic drinks | 8 | rs800578 | C | T | 0.789 | 0.012 | 0.002 | 0.000 | 25.300 |
| Alcoholic drinks | 8 | rs28601761 | G | C | 0.405 | 0.011 | 0.002 | 0.000 | 33.400 |
| Alcoholic drinks | 8 | rs9297173 | T | A | 0.500 | 0.009 | 0.002 | 0.000 | 20.900 |
| Alcoholic drinks | 8 | rs13254315 | A | G | 0.146 | 0.012 | 0.003 | 0.000 | 21.000 |
| Alcoholic drinks | 8 | rs10105127 | C | T | 0.666 | 0.009 | 0.002 | 0.000 | 21.500 |
| Alcoholic drinks | 8 | rs4380889 | A | G | 0.422 | -0.010 | 0.002 | 0.000 | 26.600 |
| Alcoholic drinks | 8 | rs10097299 | G | T | 0.194 | -0.012 | 0.002 | 0.000 | 25.800 |
| Alcoholic drinks | 8 | rs2724988 | T | G | 0.598 | 0.009 | 0.002 | 0.000 | 22.200 |
| Alcoholic drinks | 9 | rs55932213 | G | A | 0.701 | 0.012 | 0.002 | 0.000 | 31.700 |
| Alcoholic drinks | 9 | rs13288470 | T | A | 0.119 | -0.016 | 0.003 | 0.000 | 28.600 |
| Alcoholic drinks | 9 | rs138068171 | T | C | 0.040 | -0.028 | 0.006 | 0.000 | 26.200 |
| Alcoholic drinks | 9 | rs4743005 | A | G | 0.184 | -0.013 | 0.003 | 0.000 | 27.100 |
| Alcoholic drinks | 9 | rs10512093 | G | A | 0.484 | 0.009 | 0.002 | 0.000 | 24.100 |
| Alcoholic drinks | 9 | rs7043551 | C | T | 0.490 | 0.010 | 0.002 | 0.000 | 27.500 |
| Alcoholic drinks | 10 | rs10996513 | A | C | 0.047 | 0.021 | 0.004 | 0.000 | 22.400 |
| Alcoholic drinks | 10 | rs72825968 | C | T | 0.061 | -0.020 | 0.004 | 0.000 | 28.800 |
| Alcoholic drinks | 10 | rs61873510 | T | G | 0.314 | -0.011 | 0.002 | 0.000 | 28.600 |
| Alcoholic drinks | 11 | rs77232328 | T | C | 0.053 | 0.022 | 0.004 | 0.000 | 29.000 |
| Alcoholic drinks | 11 | rs2049045 | C | G | 0.189 | -0.014 | 0.003 | 0.000 | 30.200 |
| Alcoholic drinks | 11 | rs4309187 | C | A | 0.697 | 0.015 | 0.002 | 0.000 | 50.200 |
| Alcoholic drinks | 11 | rs4752999 | T | C | 0.321 | -0.015 | 0.002 | 0.000 | 49.500 |
| Alcoholic drinks | 11 | rs11231299 | A | G | 0.193 | 0.012 | 0.002 | 0.000 | 23.400 |
| Alcoholic drinks | 11 | rs17542254 | G | A | 0.251 | 0.013 | 0.002 | 0.000 | 37.500 |
| Alcoholic drinks | 12 | rs35247189 | A | C | 0.220 | -0.011 | 0.002 | 0.000 | 21.000 |
| Alcoholic drinks | 12 | rs1609028 | C | G | 0.597 | 0.010 | 0.002 | 0.000 | 24.700 |
| Alcoholic drinks | 12 | rs55863153 | C | A | 0.020 | 0.028 | 0.006 | 0.000 | 23.500 |
| Alcoholic drinks | 12 | rs2336448 | T | C | 0.418 | -0.010 | 0.002 | 0.000 | 27.400 |
| Alcoholic drinks | 12 | rs1387766 | A | G | 0.622 | -0.011 | 0.002 | 0.000 | 29.800 |
| Alcoholic drinks | 12 | rs11108953 | G | A | 0.248 | -0.011 | 0.002 | 0.000 | 23.400 |
| Alcoholic drinks | 12 | rs10876168 | T | C | 0.399 | -0.011 | 0.002 | 0.000 | 28.700 |
| Alcoholic drinks | 12 | rs61934664 | A | G | 0.476 | -0.010 | 0.002 | 0.000 | 29.100 |
| Alcoholic drinks | 12 | rs61931384 | C | T | 0.275 | -0.010 | 0.002 | 0.000 | 23.000 |
| Alcoholic drinks | 13 | rs4772260 | C | T | 0.428 | 0.009 | 0.002 | 0.000 | 21.700 |
| Alcoholic drinks | 13 | rs1927847 | C | T | 0.378 | -0.009 | 0.002 | 0.000 | 21.300 |
| Alcoholic drinks | 13 | rs943724 | A | G | 0.751 | 0.011 | 0.002 | 0.000 | 22.300 |
| Alcoholic drinks | 13 | rs4600355 | T | C | 0.562 | -0.010 | 0.002 | 0.000 | 26.300 |
| Alcoholic drinks | 14 | rs149591881 | A | G | 0.334 | 0.010 | 0.002 | 0.000 | 22.800 |
| Alcoholic drinks | 14 | rs962961 | T | C | 0.329 | -0.012 | 0.002 | 0.000 | 35.300 |
| Alcoholic drinks | 14 | rs1190979 | C | A | 0.140 | -0.015 | 0.003 | 0.000 | 27.800 |
| Alcoholic drinks | 14 | rs28929474 | T | C | 0.015 | -0.048 | 0.007 | 0.000 | 44.600 |
| Alcoholic drinks | 15 | rs35807116 | T | C | 0.562 | 0.010 | 0.002 | 0.000 | 27.200 |
| Alcoholic drinks | 15 | rs28616142 | T | C | 0.402 | 0.010 | 0.002 | 0.000 | 27.800 |
| Alcoholic drinks | 15 | rs2414133 | G | A | 0.309 | 0.011 | 0.002 | 0.000 | 24.800 |
| Alcoholic drinks | 16 | rs11860773 | C | T | 0.176 | -0.015 | 0.002 | 0.000 | 37.700 |
| Alcoholic drinks | 16 | rs13332432 | G | C | 0.296 | 0.014 | 0.002 | 0.000 | 42.800 |
| Alcoholic drinks | 16 | rs62044525 | G | C | 0.172 | -0.013 | 0.002 | 0.000 | 25.300 |
| Alcoholic drinks | 16 | rs1558902 | A | T | 0.400 | -0.010 | 0.002 | 0.000 | 26.100 |
| Alcoholic drinks | 16 | rs78815775 | G | A | 0.040 | -0.021 | 0.004 | 0.000 | 22.800 |
| Alcoholic drinks | 16 | rs79616692 | C | G | 0.110 | 0.019 | 0.003 | 0.000 | 35.600 |
| Alcoholic drinks | 16 | rs72770409 | T | C | 0.058 | -0.022 | 0.004 | 0.000 | 25.500 |
| Alcoholic drinks | 16 | rs153106 | C | T | 0.409 | -0.014 | 0.002 | 0.000 | 48.300 |
| Alcoholic drinks | 17 | rs1971157 | C | G | 0.384 | 0.010 | 0.002 | 0.000 | 24.300 |
| Alcoholic drinks | 17 | rs10438820 | T | C | 0.685 | 0.011 | 0.002 | 0.000 | 28.800 |
| Alcoholic drinks | 17 | rs2411759 | T | C | 0.429 | 0.009 | 0.002 | 0.000 | 21.100 |
| Alcoholic drinks | 17 | rs11656013 | C | T | 0.213 | -0.012 | 0.002 | 0.000 | 25.700 |
| Alcoholic drinks | 17 | rs34121753 | G | A | 0.532 | 0.011 | 0.002 | 0.000 | 32.200 |
| Alcoholic drinks | 17 | rs7502556 | C | T | 0.609 | 0.010 | 0.002 | 0.000 | 25.300 |
| Alcoholic drinks | 17 | rs79415988 | T | C | 0.261 | 0.010 | 0.002 | 0.000 | 21.200 |
| Alcoholic drinks | 17 | rs76640332 | A | G | 0.204 | -0.021 | 0.002 | 0.000 | 77.300 |
| Alcoholic drinks | 18 | rs1011392 | G | A | 0.346 | -0.011 | 0.002 | 0.000 | 29.300 |
| Alcoholic drinks | 18 | rs28597806 | G | A | 0.380 | 0.010 | 0.002 | 0.000 | 25.700 |
| Alcoholic drinks | 18 | rs1942964 | G | T | 0.477 | -0.010 | 0.002 | 0.000 | 26.800 |
| Alcoholic drinks | 19 | rs676388 | C | T | 0.494 | 0.015 | 0.002 | 0.000 | 61.100 |
| Alcoholic drinks | 20 | rs6106989 | A | G | 0.628 | 0.011 | 0.002 | 0.000 | 30.200 |
| Alcoholic drinks | 20 | rs6136466 | T | C | 0.397 | -0.009 | 0.002 | 0.000 | 23.100 |
| Alcoholic drinks | 22 | rs17884691 | A | G | 0.221 | -0.011 | 0.002 | 0.000 | 22.400 |
| Alcoholic drinks | 22 | rs9607814 | A | C | 0.212 | -0.013 | 0.003 | 0.000 | 26.100 |

**Table S4** Heterogeneity analysis of DII on sleep disorder.

| Exposure | Outcome | Method | Q | Q_df | Q_pval |
| --- | --- | --- | --- | --- | --- |
| Total cholesterol levels | Sleep apnoea | MR Egger | 306.382 | 277 | 0.108 |
| Total cholesterol levels | Sleep apnoea | Inverse variance weighted | 306.575 | 278 | 0.115 |
| Protein | Sleep apnoea | MR Egger | 516.811 | 403 | 0.001 |
| Protein | Sleep apnoea | Inverse variance weighted | 518.372 | 404 | 0.001 |
| Caffeine levels | Sleep apnoea | MR Egger | 16.941 | 15 | 0.322 |
| Caffeine levels | Sleep apnoea | Inverse variance weighted | 17.679 | 16 | 0.343 |
| Nicotinamide riboside | Sleep apnoea | MR Egger | 13.852 | 13 | 0.384 |
| Nicotinamide riboside | Sleep apnoea | Inverse variance weighted | 14.124 | 14 | 0.441 |
| Monounsaturated fatty acid levels | Sleep apnoea | MR Egger | 135.074 | 123 | 0.215 |
| Monounsaturated fatty acid levels | Sleep apnoea | Inverse variance weighted | 135.214 | 124 | 0.232 |
| Polyunsaturated fatty acid levels | Sleep apnoea | MR Egger | 128.9 | 101 | 0.032 |
| Polyunsaturated fatty acid levels | Sleep apnoea | Inverse variance weighted | 129.306 | 102 | 0.035 |
| Saturated fatty acid levels | Sleep apnoea | MR Egger | 96.602 | 89 | 0.273 |
| Saturated fatty acid levels | Sleep apnoea | Inverse variance weighted | 99.129 | 90 | 0.239 |
| Total fat | Sleep apnoea | MR Egger | 115.87 | 106 | 0.241 |
| Total fat | Sleep apnoea | Inverse variance weighted | 116.199 | 107 | 0.256 |
| Selenium | Sleep apnoea | MR Egger | 5.139 | 5 | 0.399 |
| Selenium | Sleep apnoea | Inverse variance weighted | 5.288 | 6 | 0.507 |
| Zinc | Sleep apnoea | MR Egger | 0.943 | 6 | 0.988 |
| Zinc | Sleep apnoea | Inverse variance weighted | 0.969 | 7 | 0.995 |
| Alcoholic drinks | Sleep apnoea | MR Egger | 244.005 | 120 | 0 |
| Alcoholic drinks | Sleep apnoea | Inverse variance weighted | 251.313 | 121 | 0 |
| Vitamin B2(Riboflavin) | Sleep apnoea | MR Egger | 6.863 | 5 | 0.231 |
| Vitamin B2(Riboflavin) | Sleep apnoea | Inverse variance weighted | 6.927 | 6 | 0.328 |
| Vitamin B1 | Sleep apnoea | MR Egger | 4.953 | 8 | 0.763 |
| Vitamin B1 | Sleep apnoea | Inverse variance weighted | 8.707 | 9 | 0.465 |
| Folate | Sleep apnoea | MR Egger | 10.626 | 10 | 0.387 |
| Folate | Sleep apnoea | Inverse variance weighted | 10.827 | 11 | 0.458 |
| Carotene | Sleep apnoea | MR Egger | 9.72 | 13 | 0.717 |
| Carotene | Sleep apnoea | Inverse variance weighted | 9.721 | 14 | 0.782 |
| Vitamin D | Sleep apnoea | MR Egger | 12.268 | 11 | 0.344 |
| Vitamin D | Sleep apnoea | Inverse variance weighted | 12.536 | 12 | 0.404 |
| Englyst dietary fibre | Sleep apnoea | MR Egger | 10.931 | 18 | 0.897 |
| Englyst dietary fibre | Sleep apnoea | Inverse variance weighted | 10.94 | 19 | 0.926 |
| Vitamin C | Sleep apnoea | MR Egger | 9.152 | 8 | 0.33 |
| Vitamin C | Sleep apnoea | Inverse variance weighted | 9.919 | 9 | 0.357 |
| Vitamin B12 | Sleep apnoea | MR Egger | 4.222 | 6 | 0.647 |
| Vitamin B12 | Sleep apnoea | Inverse variance weighted | 6.207 | 7 | 0.516 |
| Iron | Sleep apnoea | MR Egger | 22.915 | 9 | 0.006 |
| Iron | Sleep apnoea | Inverse variance weighted | 23.051 | 10 | 0.011 |
| Vitamin E | Sleep apnoea | MR Egger | 9.801 | 9 | 0.367 |
| Vitamin E | Sleep apnoea | Inverse variance weighted | 9.845 | 10 | 0.454 |
| Carbohydrate | Sleep apnoea | MR Egger | 11.38 | 17 | 0.836 |
| Carbohydrate | Sleep apnoea | Inverse variance weighted | 13.33 | 18 | 0.772 |
| Magnesium | Sleep apnoea | MR Egger | 10.81 | 11 | 0.459 |
| Magnesium | Sleep apnoea | Inverse variance weighted | 11.721 | 12 | 0.468 |
| Magnesium | Sleep apnoea | MR Egger | 14.829 | 15 | 0.464 |
| Magnesium | Sleep apnoea | Inverse variance weighted | 14.829 | 16 | 0.537 |
| Vitamin B6 | Sleep apnoea | MR Egger | 11.848 | 14 | 0.619 |
| Vitamin B6 | Sleep apnoea | Inverse variance weighted | 11.941 | 15 | 0.683 |
| Vitamin A | Sleep apnoea | MR Egger | 5.234 | 10 | 0.875 |
| Vitamin A | Sleep apnoea | Inverse variance weighted | 6.142 | 11 | 0.864 |
| Total cholesterol levels | Sleep-wake schedule | MR Egger | 302.181 | 278 | 0.153 |
| Total cholesterol levels | Sleep-wake schedule | Inverse variance weighted | 302.762 | 279 | 0.157 |
| Protein | Sleep-wake schedule | MR Egger | 404.344 | 403 | 0.472 |
| Protein | Sleep-wake schedule | Inverse variance weighted | 404.816 | 404 | 0.479 |
| Caffeine levels | Sleep-wake schedule | MR Egger | 19.701 | 15 | 0.184 |
| Caffeine levels | Sleep-wake schedule | Inverse variance weighted | 19.731 | 16 | 0.233 |
| Nicotinamide riboside | Sleep-wake schedule | MR Egger | 16.393 | 13 | 0.229 |
| Nicotinamide riboside | Sleep-wake schedule | Inverse variance weighted | 17.693 | 14 | 0.221 |
| Monounsaturated fatty acid levels | Sleep-wake schedule | MR Egger | 105.309 | 123 | 0.874 |
| Monounsaturated fatty acid levels | Sleep-wake schedule | Inverse variance weighted | 105.347 | 124 | 0.886 |
| Polyunsaturated fatty acid levels | Sleep-wake schedule | MR Egger | 88.628 | 101 | 0.805 |
| Polyunsaturated fatty acid levels | Sleep-wake schedule | Inverse variance weighted | 90.537 | 102 | 0.785 |
| Saturated fatty acid levels | Sleep-wake schedule | MR Egger | 101.421 | 89 | 0.174 |
| Saturated fatty acid levels | Sleep-wake schedule | Inverse variance weighted | 105.206 | 90 | 0.13 |
| Total fat | Sleep-wake schedule | MR Egger | 115.823 | 106 | 0.242 |
| Total fat | Sleep-wake schedule | Inverse variance weighted | 118.224 | 107 | 0.216 |
| Selenium | Sleep-wake schedule | MR Egger | 3.877 | 5 | 0.567 |
| Selenium | Sleep-wake schedule | Inverse variance weighted | 3.903 | 6 | 0.69 |
| Zinc | Sleep-wake schedule | MR Egger | 4.997 | 6 | 0.544 |
| Zinc | Sleep-wake schedule | Inverse variance weighted | 4.998 | 7 | 0.66 |
| Alcoholic drinks | Sleep-wake schedule | MR Egger | 102.381 | 121 | 0.889 |
| Alcoholic drinks | Sleep-wake schedule | Inverse variance weighted | 103.502 | 122 | 0.886 |
| Vitamin B2(Riboflavin) | Sleep-wake schedule | MR Egger | 4.104 | 5 | 0.535 |
| Vitamin B2(Riboflavin) | Sleep-wake schedule | Inverse variance weighted | 4.762 | 6 | 0.575 |
| Vitamin B1 | Sleep-wake schedule | MR Egger | 4.371 | 8 | 0.822 |
| Vitamin B1 | Sleep-wake schedule | Inverse variance weighted | 6.434 | 9 | 0.696 |
| Folate | Sleep-wake schedule | MR Egger | 5.399 | 10 | 0.863 |
| Folate | Sleep-wake schedule | Inverse variance weighted | 5.47 | 11 | 0.906 |
| Carotene | Sleep-wake schedule | MR Egger | 19.395 | 13 | 0.111 |
| Carotene | Sleep-wake schedule | Inverse variance weighted | 19.504 | 14 | 0.147 |
| Vitamin D | Sleep-wake schedule | MR Egger | 13.061 | 11 | 0.289 |
| Vitamin D | Sleep-wake schedule | Inverse variance weighted | 15.055 | 12 | 0.238 |
| Englyst dietary fibre | Sleep-wake schedule | MR Egger | 9.066 | 18 | 0.958 |
| Englyst dietary fibre | Sleep-wake schedule | Inverse variance weighted | 10.743 | 19 | 0.932 |
| Vitamin C | Sleep-wake schedule | MR Egger | 7.683 | 8 | 0.465 |
| Vitamin C | Sleep-wake schedule | Inverse variance weighted | 7.69 | 9 | 0.566 |
| Vitamin B12 | Sleep-wake schedule | MR Egger | 14.141 | 6 | 0.028 |
| Vitamin B12 | Sleep-wake schedule | Inverse variance weighted | 14.178 | 7 | 0.048 |
| Iron | Sleep-wake schedule | MR Egger | 5.706 | 9 | 0.769 |
| Iron | Sleep-wake schedule | Inverse variance weighted | 7.542 | 10 | 0.674 |
| Vitamin E | Sleep-wake schedule | MR Egger | 9.938 | 9 | 0.356 |
| Vitamin E | Sleep-wake schedule | Inverse variance weighted | 10.037 | 10 | 0.437 |
| Carbohydrate | Sleep-wake schedule | MR Egger | 17.037 | 17 | 0.452 |
| Carbohydrate | Sleep-wake schedule | Inverse variance weighted | 17.998 | 18 | 0.456 |
| Magnesium | Sleep-wake schedule | MR Egger | 6.328 | 11 | 0.851 |
| Magnesium | Sleep-wake schedule | Inverse variance weighted | 6.366 | 12 | 0.897 |
| Magnesium | Sleep-wake schedule | MR Egger | 15.503 | 15 | 0.416 |
| Magnesium | Sleep-wake schedule | Inverse variance weighted | 17.014 | 16 | 0.385 |
| Vitamin B6 | Sleep-wake schedule | MR Egger | 13.113 | 14 | 0.518 |
| Vitamin B6 | Sleep-wake schedule | Inverse variance weighted | 13.138 | 15 | 0.592 |
| Vitamin A | Sleep-wake schedule | MR Egger | 13.558 | 10 | 0.194 |
| Vitamin A | Sleep-wake schedule | Inverse variance weighted | 15.851 | 11 | 0.147 |
| Total cholesterol levels | Insomnia | MR Egger | 285.974 | 278 | 0.358 |
| Total cholesterol levels | Insomnia | Inverse variance weighted | 288.145 | 279 | 0.340 |
| Protein | Insomnia | MR Egger | 429.350 | 403 | 0.176 |
| Protein | Insomnia | Inverse variance weighted | 429.759 | 404 | 0.181 |
| Caffeine levels | Insomnia | MR Egger | 11.443 | 15 | 0.721 |
| Caffeine levels | Insomnia | Inverse variance weighted | 11.497 | 16 | 0.778 |
| Nicotinamide riboside | Insomnia | MR Egger | 13.111 | 13 | 0.439 |
| Nicotinamide riboside | Insomnia | Inverse variance weighted | 13.754 | 14 | 0.468 |
| Monounsaturated fatty acid levels | Insomnia | MR Egger | 87.969 | 123 | 0.993 |
| Monounsaturated fatty acid levels | Insomnia | Inverse variance weighted | 87.969 | 124 | 0.994 |
| Polyunsaturated fatty acid levels | Insomnia | MR Egger | 76.108 | 101 | 0.969 |
| Polyunsaturated fatty acid levels | Insomnia | Inverse variance weighted | 76.739 | 102 | 0.971 |
| Saturated fatty acid levels | Insomnia | MR Egger | 83.252 | 89 | 0.652 |
| Saturated fatty acid levels | Insomnia | Inverse variance weighted | 83.574 | 90 | 0.670 |
| Total fat | Insomnia | MR Egger | 102.876 | 106 | 0.568 |
| Total fat | Insomnia | Inverse variance weighted | 102.999 | 107 | 0.591 |
| Selenium | Insomnia | MR Egger | 6.861 | 5 | 0.231 |
| Selenium | Insomnia | Inverse variance weighted | 6.861 | 6 | 0.334 |
| Zinc | Insomnia | MR Egger | 17.945 | 6 | 0.006 |
| Zinc | Insomnia | Inverse variance weighted | 17.945 | 7 | 0.012 |
| Alcoholic drinks | Insomnia | MR Egger | 113.896 | 121 | 0.664 |
| Alcoholic drinks | Insomnia | Inverse variance weighted | 114.783 | 122 | 0.666 |
| Vitamin B2(Riboflavin) | Insomnia | MR Egger | 0.957 | 5 | 0.966 |
| Vitamin B2(Riboflavin) | Insomnia | Inverse variance weighted | 3.698 | 6 | 0.717 |
| Vitamin B1 | Insomnia | MR Egger | 6.328 | 8 | 0.611 |
| Vitamin B1 | Insomnia | Inverse variance weighted | 6.779 | 9 | 0.660 |
| Folate | Insomnia | MR Egger | 6.454 | 10 | 0.776 |
| Folate | Insomnia | Inverse variance weighted | 6.466 | 11 | 0.841 |
| Carotene | Insomnia | MR Egger | 10.243 | 13 | 0.674 |
| Carotene | Insomnia | Inverse variance weighted | 10.862 | 14 | 0.697 |
| Vitamin D | Insomnia | MR Egger | 8.199 | 11 | 0.695 |
| Vitamin D | Insomnia | Inverse variance weighted | 9.001 | 12 | 0.703 |
| Englyst dietary fibre | Insomnia | MR Egger | 15.876 | 18 | 0.601 |
| Englyst dietary fibre | Insomnia | Inverse variance weighted | 16.150 | 19 | 0.647 |
| Vitamin C | Insomnia | MR Egger | 7.164 | 8 | 0.519 |
| Vitamin C | Insomnia | Inverse variance weighted | 9.349 | 9 | 0.406 |
| Vitamin B12 | Insomnia | MR Egger | 9.191 | 6 | 0.163 |
| Vitamin B12 | Insomnia | Inverse variance weighted | 9.424 | 7 | 0.224 |
| Iron | Insomnia | MR Egger | 6.440 | 9 | 0.695 |
| Iron | Insomnia | Inverse variance weighted | 6.463 | 10 | 0.775 |
| Vitamin E | Insomnia | MR Egger | 12.143 | 9 | 0.205 |
| Vitamin E | Insomnia | Inverse variance weighted | 12.669 | 10 | 0.243 |
| Carbohydrate | Insomnia | MR Egger | 19.219 | 17 | 0.316 |
| Carbohydrate | Insomnia | Inverse variance weighted | 19.221 | 18 | 0.378 |
| Magnesium | Insomnia | MR Egger | 8.491 | 11 | 0.669 |
| Magnesium | Insomnia | Inverse variance weighted | 8.751 | 12 | 0.724 |
| Magnesium | Insomnia | MR Egger | 22.230 | 15 | 0.102 |
| Magnesium | Insomnia | Inverse variance weighted | 24.628 | 16 | 0.077 |
| Vitamin B6 | Insomnia | MR Egger | 14.426 | 14 | 0.418 |
| Vitamin B6 | Insomnia | Inverse variance weighted | 14.529 | 15 | 0.486 |
| Vitamin A | Insomnia | MR Egger | 17.438 | 10 | 0.065 |
| Vitamin A | Insomnia | Inverse variance weighted | 17.604 | 11 | 0.091 |
| Total cholesterol levels | Hypersomnia | MR Egger | 286.751 | 278 | 0.346 |
| Total cholesterol levels | Hypersomnia | Inverse variance weighted | 287.753 | 279 | 0.346 |
| Protein | Hypersomnia | MR Egger | 447.648 | 403 | 0.062 |
| Protein | Hypersomnia | Inverse variance weighted | 447.661 | 404 | 0.066 |
| Caffeine levels | Hypersomnia | MR Egger | 12.569 | 15 | 0.636 |
| Caffeine levels | Hypersomnia | Inverse variance weighted | 12.588 | 16 | 0.703 |
| Nicotinamide riboside | Hypersomnia | MR Egger | 7.347 | 13 | 0.883 |
| Nicotinamide riboside | Hypersomnia | Inverse variance weighted | 7.454 | 14 | 0.916 |
| Monounsaturated fatty acid levels | Hypersomnia | MR Egger | 138.740 | 123 | 0.157 |
| Monounsaturated fatty acid levels | Hypersomnia | Inverse variance weighted | 139.566 | 124 | 0.161 |
| Polyunsaturated fatty acid levels | Hypersomnia | MR Egger | 92.304 | 101 | 0.720 |
| Polyunsaturated fatty acid levels | Hypersomnia | Inverse variance weighted | 92.747 | 102 | 0.733 |
| Saturated fatty acid levels | Hypersomnia | MR Egger | 96.381 | 89 | 0.278 |
| Saturated fatty acid levels | Hypersomnia | Inverse variance weighted | 97.552 | 90 | 0.275 |
| Total fat | Hypersomnia | MR Egger | 110.452 | 106 | 0.364 |
| Total fat | Hypersomnia | Inverse variance weighted | 111.596 | 107 | 0.361 |
| Selenium | Hypersomnia | MR Egger | 3.919 | 5 | 0.561 |
| Selenium | Hypersomnia | Inverse variance weighted | 3.968 | 6 | 0.681 |
| Zinc | Hypersomnia | MR Egger | 8.631 | 6 | 0.195 |
| Zinc | Hypersomnia | Inverse variance weighted | 15.235 | 7 | 0.033 |
| Alcoholic drinks | Hypersomnia | MR Egger | 114.559 | 121 | 0.647 |
| Alcoholic drinks | Hypersomnia | Inverse variance weighted | 114.718 | 122 | 0.667 |
| Vitamin B2(Riboflavin) | Hypersomnia | MR Egger | 6.260 | 5 | 0.282 |
| Vitamin B2(Riboflavin) | Hypersomnia | Inverse variance weighted | 10.058 | 6 | 0.122 |
| Vitamin B1 | Hypersomnia | MR Egger | 5.424 | 8 | 0.711 |
| Vitamin B1 | Hypersomnia | Inverse variance weighted | 5.990 | 9 | 0.741 |
| Folate | Hypersomnia | MR Egger | 10.710 | 10 | 0.381 |
| Folate | Hypersomnia | Inverse variance weighted | 10.805 | 11 | 0.460 |
| Carotene | Hypersomnia | MR Egger | 13.556 | 13 | 0.406 |
| Carotene | Hypersomnia | Inverse variance weighted | 13.583 | 14 | 0.481 |
| Vitamin D | Hypersomnia | MR Egger | 10.515 | 11 | 0.485 |
| Vitamin D | Hypersomnia | Inverse variance weighted | 11.591 | 12 | 0.479 |
| Englyst dietary fibre | Hypersomnia | MR Egger | 16.382 | 18 | 0.566 |
| Englyst dietary fibre | Hypersomnia | Inverse variance weighted | 16.492 | 19 | 0.624 |
| Vitamin C | Hypersomnia | MR Egger | 23.878 | 8 | 0.002 |
| Vitamin C | Hypersomnia | Inverse variance weighted | 24.254 | 9 | 0.004 |
| Vitamin B12 | Hypersomnia | MR Egger | 4.332 | 6 | 0.632 |
| Vitamin B12 | Hypersomnia | Inverse variance weighted | 5.946 | 7 | 0.546 |
| Iron | Hypersomnia | MR Egger | 14.658 | 9 | 0.101 |
| Iron | Hypersomnia | Inverse variance weighted | 16.067 | 10 | 0.098 |
| Vitamin E | Hypersomnia | MR Egger | 6.451 | 9 | 0.694 |
| Vitamin E | Hypersomnia | Inverse variance weighted | 7.577 | 10 | 0.670 |
| Carbohydrate | Hypersomnia | MR Egger | 28.093 | 17 | 0.044 |
| Carbohydrate | Hypersomnia | Inverse variance weighted | 28.108 | 18 | 0.060 |
| Magnesium | Hypersomnia | MR Egger | 15.046 | 11 | 0.180 |
| Magnesium | Hypersomnia | Inverse variance weighted | 15.430 | 12 | 0.219 |
| Magnesium | Hypersomnia | MR Egger | 24.171 | 15 | 0.062 |
| Magnesium | Hypersomnia | Inverse variance weighted | 24.851 | 16 | 0.072 |
| Vitamin B6 | Hypersomnia | MR Egger | 16.062 | 14 | 0.310 |
| Vitamin B6 | Hypersomnia | Inverse variance weighted | 16.065 | 15 | 0.378 |
| Vitamin A | Hypersomnia | MR Egger | 4.832 | 10 | 0.902 |
| Vitamin A | Hypersomnia | Inverse variance weighted | 5.590 | 11 | 0.899 |

**Table S5** Pleiotropy analysis of DII on sleep disorder.

| Exposure | Outcome | Egger_intercept | Se | Pval |
| --- | --- | --- | --- | --- |
| Total cholesterol levels | Sleep apnoea | -0.001 | 0.001 | 0.677 |
| Protein | Sleep apnoea | -0.002 | 0.002 | 0.271 |
| Caffeine levels | Sleep apnoea | 0.010 | 0.012 | 0.431 |
| Nicotinamide riboside | Sleep apnoea | -0.006 | 0.012 | 0.622 |
| Monounsaturated fatty acid levels | Sleep apnoea | 0.001 | 0.002 | 0.721 |
| Polyunsaturated fatty acid levels | Sleep apnoea | 0.002 | 0.003 | 0.574 |
| Saturated fatty acid levels | Sleep apnoea | 0.005 | 0.003 | 0.131 |
| Total fat | Sleep apnoea | 0.002 | 0.003 | 0.584 |
| Selenium | Sleep apnoea | 0.008 | 0.020 | 0.719 |
| Zinc | Sleep apnoea | -0.003 | 0.020 | 0.878 |
| Alcoholic drinks | Sleep apnoea | -0.010 | 0.005 | 0.060 |
| Vitamin B2(Riboflavin) | Sleep apnoea | -0.003 | 0.015 | 0.838 |
| Vitamin B1 | Sleep apnoea | 0.034 | 0.017 | 0.089 |
| Folate | Sleep apnoea | 0.005 | 0.012 | 0.673 |
| Carotene | Sleep apnoea | 0.000 | 0.011 | 0.967 |
| Vitamin D | Sleep apnoea | 0.010 | 0.021 | 0.634 |
| Englyst dietary fibre | Sleep apnoea | -0.001 | 0.010 | 0.923 |
| Vitamin C | Sleep apnoea | 0.012 | 0.015 | 0.437 |
| Vitamin B12 | Sleep apnoea | 0.022 | 0.015 | 0.209 |
| Iron | Sleep apnoea | 0.005 | 0.023 | 0.823 |
| Vitamin E | Sleep apnoea | -0.002 | 0.011 | 0.845 |
| Carbohydrate | Sleep apnoea | 0.014 | 0.010 | 0.181 |
| Magnesium | Sleep apnoea | 0.013 | 0.014 | 0.360 |
| Magnesium | Sleep apnoea | 0.000 | 0.010 | 0.979 |
| Vitamin B6 | Sleep apnoea | 0.003 | 0.011 | 0.765 |
| Vitamin A | Sleep apnoea | 0.018 | 0.019 | 0.363 |
| Total cholesterol levels | Sleep-wake schedule | -0.009 | 0.012 | 0.465 |
| Protein | Sleep-wake schedule | -0.008 | 0.012 | 0.493 |
| Caffeine levels | Sleep-wake schedule | -0.016 | 0.105 | 0.883 |
| Nicotinamide riboside | Sleep-wake schedule | 0.112 | 0.110 | 0.328 |
| Monounsaturated fatty acid levels | Sleep-wake schedule | -0.004 | 0.020 | 0.845 |
| Polyunsaturated fatty acid levels | Sleep-wake schedule | 0.033 | 0.024 | 0.170 |
| Saturated fatty acid levels | Sleep-wake schedule | 0.048 | 0.026 | 0.072 |
| Total fat | Sleep-wake schedule | 0.034 | 0.023 | 0.141 |
| Selenium | Sleep-wake schedule | -0.026 | 0.162 | 0.877 |
| Zinc | Sleep-wake schedule | -0.004 | 0.165 | 0.984 |
| Alcoholic drinks | Sleep-wake schedule | -0.031 | 0.030 | 0.292 |
| Vitamin B2(Riboflavin) | Sleep-wake schedule | -0.083 | 0.103 | 0.454 |
| Vitamin B1 | Sleep-wake schedule | -0.206 | 0.143 | 0.189 |
| Folate | Sleep-wake schedule | -0.025 | 0.096 | 0.796 |
| Carotene | Sleep-wake schedule | -0.029 | 0.108 | 0.791 |
| Vitamin D | Sleep-wake schedule | -0.228 | 0.176 | 0.222 |
| Englyst dietary fibre | Sleep-wake schedule | 0.112 | 0.086 | 0.212 |
| Vitamin C | Sleep-wake schedule | -0.009 | 0.114 | 0.939 |
| Vitamin B12 | Sleep-wake schedule | -0.025 | 0.196 | 0.903 |
| Iron | Sleep-wake schedule | 0.158 | 0.117 | 0.208 |
| Vitamin E | Sleep-wake schedule | -0.028 | 0.092 | 0.771 |
| Carbohydrate | Sleep-wake schedule | 0.084 | 0.086 | 0.341 |
| Magnesium | Sleep-wake schedule | 0.022 | 0.114 | 0.849 |
| Magnesium | Sleep-wake schedule | 0.100 | 0.083 | 0.245 |
| Vitamin B6 | Sleep-wake schedule | 0.015 | 0.093 | 0.877 |
| Vitamin A | Sleep-wake schedule | -0.238 | 0.183 | 0.223 |
| Total cholesterol levels | Insomnia | -0.006 | 0.004 | 0.147 |
| Protein | Insomnia | -0.003 | 0.004 | 0.536 |
| Caffeine levels | Insomnia | 0.007 | 0.031 | 0.820 |
| Nicotinamide riboside | Insomnia | 0.027 | 0.033 | 0.439 |
| Monounsaturated fatty acid levels | Insomnia | 0.000 | 0.007 | 0.991 |
| Polyunsaturated fatty acid levels | Insomnia | -0.006 | 0.008 | 0.429 |
| Saturated fatty acid levels | Insomnia | -0.005 | 0.008 | 0.572 |
| Total fat | Insomnia | -0.003 | 0.007 | 0.727 |
| Selenium | Insomnia | 0.000 | 0.064 | 0.998 |
| Zinc | Insomnia | 0.000 | 0.097 | 0.997 |
| Alcoholic drinks | Insomnia | -0.010 | 0.010 | 0.348 |
| Vitamin B2(Riboflavin) | Insomnia | 0.058 | 0.035 | 0.159 |
| Vitamin B1 | Insomnia | 0.033 | 0.049 | 0.521 |
| Folate | Insomnia | -0.004 | 0.033 | 0.916 |
| Carotene | Insomnia | -0.024 | 0.030 | 0.445 |
| Vitamin D | Insomnia | -0.049 | 0.055 | 0.390 |
| Englyst dietary fibre | Insomnia | 0.015 | 0.029 | 0.607 |
| Vitamin C | Insomnia | 0.057 | 0.039 | 0.178 |
| Vitamin B12 | Insomnia | -0.021 | 0.054 | 0.710 |
| Iron | Insomnia | 0.006 | 0.039 | 0.882 |
| Vitamin E | Insomnia | -0.022 | 0.034 | 0.548 |
| Carbohydrate | Insomnia | -0.001 | 0.031 | 0.967 |
| Magnesium | Insomnia | -0.020 | 0.039 | 0.620 |
| Magnesium | Insomnia | -0.043 | 0.034 | 0.223 |
| Vitamin B6 | Insomnia | -0.010 | 0.032 | 0.757 |
| Vitamin A | Insomnia | -0.022 | 0.071 | 0.764 |
| Total cholesterol levels | Hypersomnia | -0.012 | 0.012 | 0.325 |
| Protein | Hypersomnia | -0.001 | 0.013 | 0.914 |
| Caffeine levels | Hypersomnia | -0.013 | 0.094 | 0.893 |
| Nicotinamide riboside | Hypersomnia | 0.033 | 0.101 | 0.748 |
| Monounsaturated fatty acid levels | Hypersomnia | 0.018 | 0.021 | 0.394 |
| Polyunsaturated fatty acid levels | Hypersomnia | -0.016 | 0.024 | 0.507 |
| Saturated fatty acid levels | Hypersomnia | 0.027 | 0.026 | 0.301 |
| Total fat | Hypersomnia | 0.024 | 0.023 | 0.297 |
| Selenium | Hypersomnia | 0.037 | 0.166 | 0.833 |
| Zinc | Hypersomnia | -0.435 | 0.203 | 0.076 |
| Alcoholic drinks | Hypersomnia | 0.012 | 0.031 | 0.690 |
| Vitamin B2(Riboflavin) | Hypersomnia | 0.206 | 0.118 | 0.142 |
| Vitamin B1 | Hypersomnia | 0.110 | 0.147 | 0.473 |
| Folate | Hypersomnia | -0.030 | 0.102 | 0.772 |
| Carotene | Hypersomnia | -0.015 | 0.093 | 0.876 |
| Vitamin D | Hypersomnia | 0.173 | 0.166 | 0.322 |
| Englyst dietary fibre | Hypersomnia | 0.029 | 0.089 | 0.743 |
| Vitamin C | Hypersomnia | -0.071 | 0.201 | 0.732 |
| Vitamin B12 | Hypersomnia | 0.167 | 0.131 | 0.251 |
| Iron | Hypersomnia | 0.142 | 0.152 | 0.377 |
| Vitamin E | Hypersomnia | 0.095 | 0.090 | 0.316 |
| Carbohydrate | Hypersomnia | -0.011 | 0.113 | 0.927 |
| Magnesium | Hypersomnia | 0.073 | 0.137 | 0.607 |
| Magnesium | Hypersomnia | -0.069 | 0.106 | 0.526 |
| Vitamin B6 | Hypersomnia | -0.005 | 0.103 | 0.959 |
| Vitamin A | Hypersomnia | 0.141 | 0.161 | 0.404 |
